# Unsupported Mg–Alkene Bonding

**DOI:** 10.1002/chem.202004716

**Published:** 2020-12-23

**Authors:** Katharina Thum, Alexander Friedrich, Jürgen Pahl, Holger Elsen, Jens Langer, Sjoerd Harder

**Affiliations:** ^1^ Chair of Inorganic and Organometallic Chemistry Universität Erlangen-Nürnberg Egerlandstrasse 1 91058 Erlangen Germany

**Keywords:** alkene ligands, density functional calculations, Lewis acids, magnesium, noncovalent interactions

## Abstract

The first intermolecular early main group metal–alkene complexes were isolated. This was enabled by using highly Lewis acidic Mg centers in the Lewis base‐free cations (^Me^BDI)Mg^+^ and (^*t*Bu^BDI)Mg^+^ with B(C_6_F_5_)_4_^−^ counterions (^Me^BDI=CH[C(CH_3_)N(DIPP)]_2_, ^*t*Bu^BDI=CH[C(*t*Bu)N(DIPP)]_2_, DIPP=2,6‐diisopropylphenyl). Coordination complexes with various mono‐ and *bis*‐alkene ligands, typically used in transition metal chemistry, were structurally characterized for 1,3‐divinyltetramethyldisiloxane, 1,5‐cyclooctadiene, cyclooctene, 1,3,5‐cycloheptatriene, 2,3‐dimethylbuta‐1,3‐diene, and 2‐ethyl‐1‐butene. In all cases, asymmetric Mg–alkene bonding with a short and a long Mg−C bond is observed. This asymmetry is most extreme for Mg–(H_2_C=CEt_2_) bonding. In bromobenzene solution, the Mg–alkene complexes are either dissociated or in a dissociation equilibrium. A DFT study and AIM analysis showed that the C=C bonds hardly change on coordination and there is very little alkene→Mg electron transfer. The Mg–alkene bonds are mainly electrostatic and should be described as Mg^2+^ ion‐induced dipole interactions.

## Graphical abstract

**Weak but important**: The first unsupported alkene complexes of an s‐block metal have been realized. Alkene–magnesium coordination is in all cases asymmetric and leads to polarization of π‐electron density in the C=C bond. There is no alkene→Mg charge transfer and only minimal C=C bond lengthening. Although weak, these interactions are important in early main group metal catalysis.

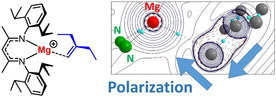

## Introduction

**Scheme 1:**
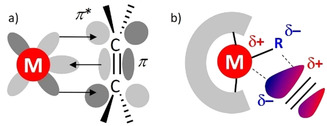
C=C bond activation by transition metals (a) and group 2 metals (b).

Neutral π ligands are common in transition metal chemistry.^[1]^ In fact, the first organometallic compound isolated in pure form was a platinum ethylene complex. Commonly referred to as Zeise's salt, K_2_[PtCl_3_(C_2_H_4_)]**⋅**H_2_O was isolated already as early as 1827.[^2^, ^3^] Although composition and bonding mode were at that time not fully understood, this early pioneering work marks the beginning of metal–alkene chemistry.^[4]^ Detailed knowledge of its structure led to formulation of the Dewar–Chatt–Duncanson bonding model in which σ bonding between the ethylene HOMO and an empty metal orbital depletes electron density in a π‐bonding molecular orbital, while d→π* backbonding fills the antibonding molecular orbital (Scheme 1 a). This combined action results in activation and elongation of the ethylene C=C bond from 1.336(1) Å (free ethylene)^[5]^ to 1.375(4) Å.^[6]^ Comprehensive Raman studies on Zeise's salt suggest strong Pt−C bonding and considerable weakening of the C=C bond, indicated by a shift of the C=C stretching frequency from 1653 cm^−1^ in free ethylene to 1243 cm^−1^ in the Pt complex.^[7]^ Bond activation by metal–alkene coordination is therefore a crucial first step in catalytic alkene conversion.[^8^, ^9^, ^10^, ^11^, ^12^]

**Scheme 2:**
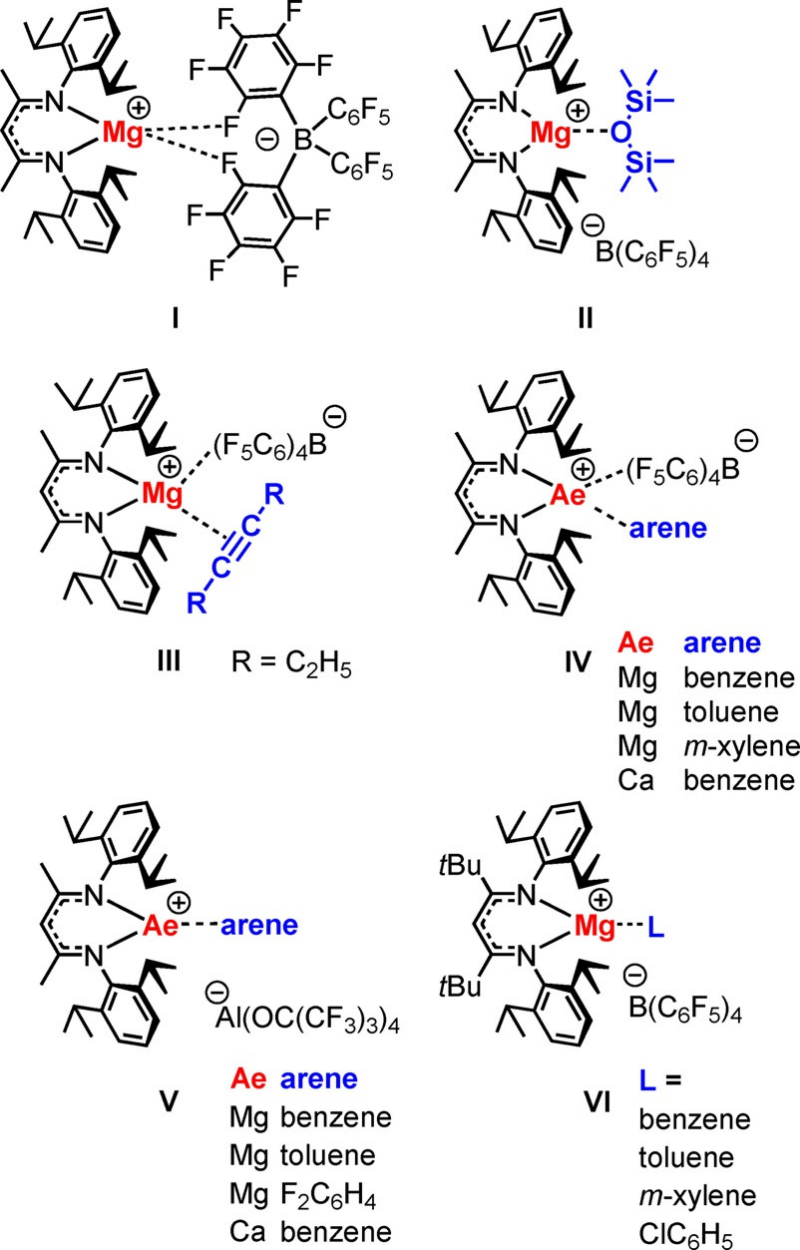
Examples of cationic (^Me^BDI)Ae^+^ and (^*t*Bu^BDI)Ae^+^ complexes (^Me^BDI=CH[C(CH_3_)N(DIPP)]_2_, ^*t*Bu^BDI=CH[C(*t*Bu)N(DIPP)]_2_, DIPP=2,6‐diisopropylphenyl).

The most striking difference between the transition and main group metals is the inability of the latter to strongly bind π ligands. This has its origin in their lack of low‐lying, partially filled, d orbitals. Despite this deficiency, metal–alkene coordination is often proposed as a first elementary step in alkaline earth (Ae) metal catalyzed alkene conversion.[^13^, ^14^, ^15^] Although this type of bonding is very weak and not well understood, it is in some cases crucial for catalytic activity.^[16]^ Early main group metals activate substrates only by metal Lewis acid⋅⋅⋅alkene interaction. Especially an asymmetric metal alkene coordination mode leads to polarization of the C=C bond, which induces the *δ*+/*δ*− charge separation necessary for nucleophilic attack (Scheme 1 b).^[16]^ Proof for such weak interactions was mainly found by switching off the detrimental effect of entropy loss, and therefore the vast majority of all main group metal–alkene interactions are of the intramolecular type.[^17^, ^18^, ^19^, ^20^, ^21^, ^22^, ^23^, ^24^, ^25^] The first unsupported metal–alkene bonding between the p‐block metal Ga and 1,5‐cyclooctadiene (cod) has been realized very recently in the homoleptic complex cation Ga(cod)_2_^+^.^[26]^ Like in transition metal chemistry, the chelating coordination mode of this bis‐alkene certainly contributes to its stability. Despite the importance of s‐block metal–alkene bonding in catalysis, unsupported metal–alkene bonding has so far not been described. We recently reported a cationic β‐diketiminate (BDI) Mg complex in which the metal center is unsolvated, only showing Mg⋅⋅⋅F interactions with the weakly coordinating anion (WCA) B(C_6_F_5_)_4_^−^ (**I** in Scheme 2).^[27]^ We demonstrated its considerable Lewis acidity by isolation of the first unsupported complexes Mg⋅⋅⋅O(SiMe_3_)_2_ (**II**)^[28]^ and Mg⋅⋅⋅(3‐hexyne) (**III**)[^27^, ^29^]. We also reported a series of cationic Mg and Ca π‐arene complexes **IV**.[^27^, ^30^] Hill et al. simultaneously published similar complexes **V** ^[31]^ that contain the less coordinating Krossing anion Al[OC(CF_3_)_3_]_4_^−^ and are devoid of metal⋅⋅⋅anion interactions. Cation–anion interactions can also be avoided by increasing the bulk of the BDI ligand. Replacing the Me groups in the BDI ligand backbone with *t*Bu substituents led to complete cleavage of cation–anion contacts (**VI**).^[32]^ Krossing et al. recently described Ae^2+^(hexamethylbenzene) ions (Ae=Ca, Sr, Ba) stabilized by weakly coordinating aluminate anions^[33]^ and most recently introduced a catalytically active dicationic *ansa*‐arene Sr^2+^ complex.^[34]^

Although the interaction between Ae metal cations and electron‐rich π‐arene or π‐alkyne ligands has been comprehensively investigated, there is currently no information on unsupported Ae metal–alkene complexes. Considering their important role as intermediates in catalysis, we now fill this gap by reporting a variety of Mg complexes with intermolecular bis‐ and mono‐alkene ligands, discuss metal–alkene bonding, and provide a DFT study that gives insight into their relative bond energies.

## Results and Discussion

### Synthesis

Synthesis of Mg complexes with weakly bound alkene ligands is challenging, and certain requirements should be taken into consideration. The solvent will compete with a weak Mg⋅⋅⋅alkene interaction. Therefore, not only ethereal solvents but also aromatic solvents such as benzene or toluene should be avoided, and instead halogenated solvents must be used. During the course of our investigations we found that chlorobenzene is preferable to the more polar and more strongly coordinating fluorobenzene. As has been shown by Hill and co‐workers, fluoroarenes can indeed tightly bind to (^Me^BDI)Ae^+^.^[31]^ Due to competition between Mg⋅⋅⋅solvent and Mg⋅⋅⋅alkene interactions, it is also advisable to use a large excess of the alkene. Another factor to be considered is competition between Mg⋅⋅⋅alkene bonding and cation–anion interactions. Although the B(C_6_F_5_)_4_^−^ anion is only weakly coordinating, short Mg⋅⋅⋅F interactions may interrupt Mg⋅⋅⋅alkene bonding. We recently reported that increased steric bulk in the backbone of the BDI ligand fully impedes the Mg⋅⋅⋅B(C_6_F_5_)_4_^−^ interaction, which even allowed for isolation of a complex with weakly coordinating chlorobenzene (**VI**).^[32]^ Therefore, our first efforts to isolate Mg alkene complexes concentrated on using the strong Lewis acid (^*t*Bu^BDI)Mg^+^.

**Scheme 3:**
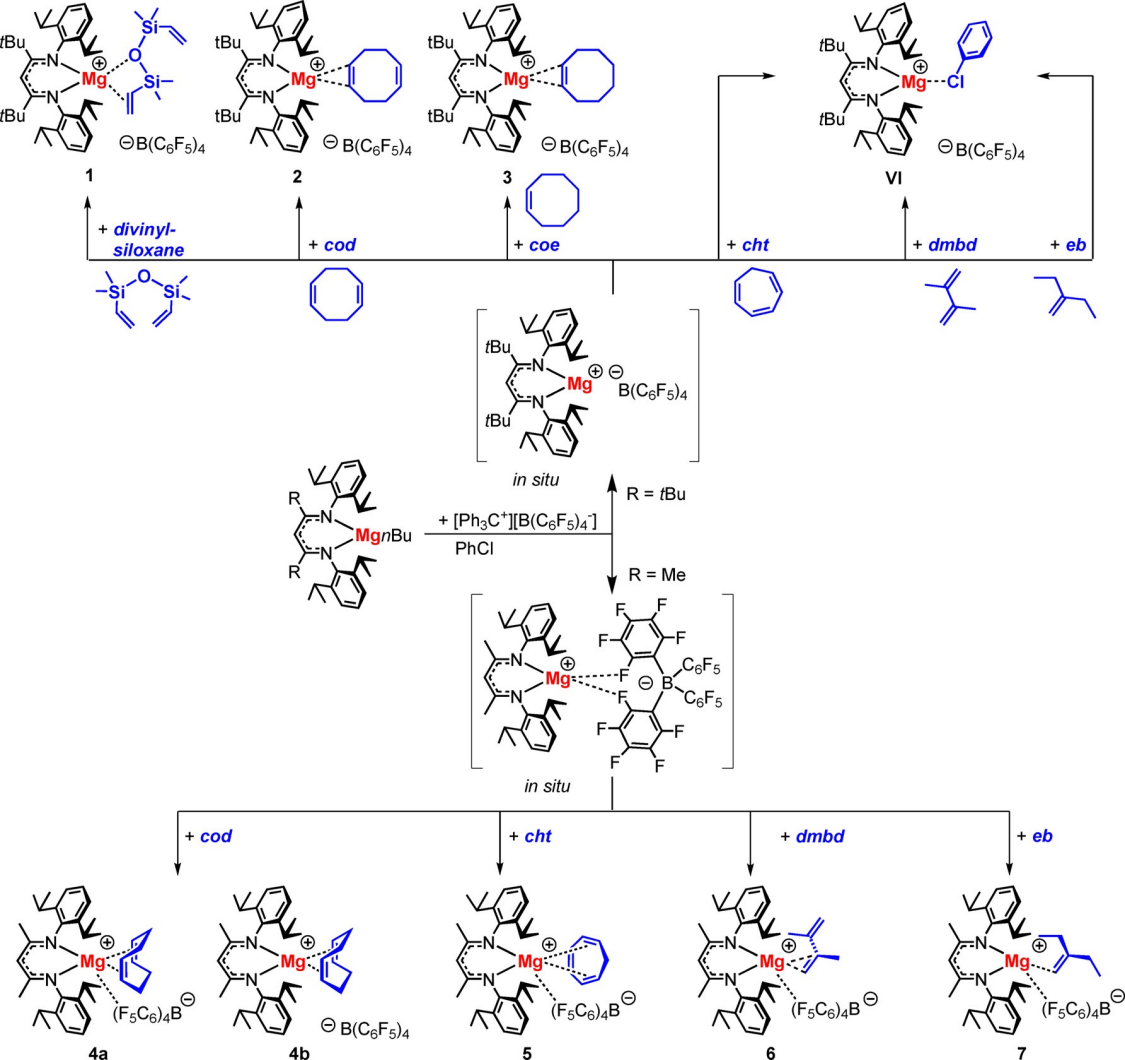
Synthesis of [(^R^BDI)Mg^+^(alkene)][B(C_6_F_5_)_4_^−^] complexes with R=*t*Bu or Me. Note that the sterically congested (^*t*Bu^BDI)Mg^+^ does not form complexes with cht, dmbd and eb due to competition with the solvent.

The (^*t*Bu^BDI)Mg^+^ cation was generated in situ by reaction of (^*t*Bu^BDI)Mg*n*Bu with [Ph_3_C^+^][B(C_6_F_5_)_4_^−^] in chlorobenzene. In the presence of 1,3‐divinyltetramethyldisiloxane (abbreviated as divinylsiloxane), a bis‐alkene ligand used to stabilize Pt^0^ in the Karstedt catalyst,^[35]^ [(^*t*Bu^BDI)Mg^+^(divinylsiloxane)][B(C_6_F_5_)_4_^−^] (**1**) was obtained in 41 % yield of crystalline product (Scheme 3). Crystal structure determination (vide infra) showed that, other than in the Pt complex, only one alkene ligand is bound to the Mg center. In addition to this Mg–alkene bond, a weak Mg–O[SiMe_2_(vinyl)]_2_ interaction is observed. The first unsupported metal siloxane complex with the simplest silyl ether O(SiMe_3_)_2_ (**II**) was only isolated recently and this unusual bond should be classified as weak.^[28]^

Also cod, a popular chelating diene for stabilization of transition metal complexes,[^36^, ^37^] formed a stable Mg–alkene complex (**2**). However, due to the restricted coordination sphere around Mg, the cod ligand coordinates only with one of its alkene groups. The mono‐alkene cyclooctene (coe) gave the similar complex **3**. Attempts to isolate complexes with 1,3,5‐cycloheptatriene (cht), 2,3‐dimethyl‐buta‐1,3‐diene (dmbd) or 2‐ethyl‐1‐butene (eb) failed and in all cases complexation of the solvent to give the recently reported [(^*t*Bu^BDI)Mg^+^(ClC_6_H_5_)][B(C_6_F_5_)_4_^−^]^[32]^ was observed.

Since dienes such as divinylsiloxane or cod gave stable Mg–alkene complexes in which only one of the alkene bonds directly interacts with the metal center, also alkene complexation with the less bulky β‐diketiminate ligand ^Me^BDI was attempted. Reaction of (^Me^BDI)Mg*n*Bu with [Ph_3_C^+^][B(C_6_F_5_)_4_^−^] in chlorobenzene led to in situ formation of the cation–anion pair [(^Me^BDI)Mg^+^][B(C_6_F_5_)_4_^−^]. The much more open coordination sphere in (^Me^BDI)Mg^+^ allowed for isolation of an Mg–alkene complex with a chelating cod ligand (**4**, Scheme 3). Interestingly, the asymmetric unit of the crystal structure of **4** contains five independent (^Me^BDI)Mg^+^(η^4^‐cod) cations, which can be divided into two types of structures, that is, those with an additional Mg⋅⋅⋅F contact (**4 a**) or those without cation–anion interaction (**4 b**). The smaller β‐diketiminate ligand also enabled isolation of complexes with cht (**5**), dmbd (**6**), and eb (**7**), that is, ligands that could not form complexes with the bulkier (^*t*Bu^BDI)Mg^+^. The Mg⋅⋅⋅alkene interaction is in all cases accompanied by a cation–anion contact.

### Crystal structures

**Figure 1:**
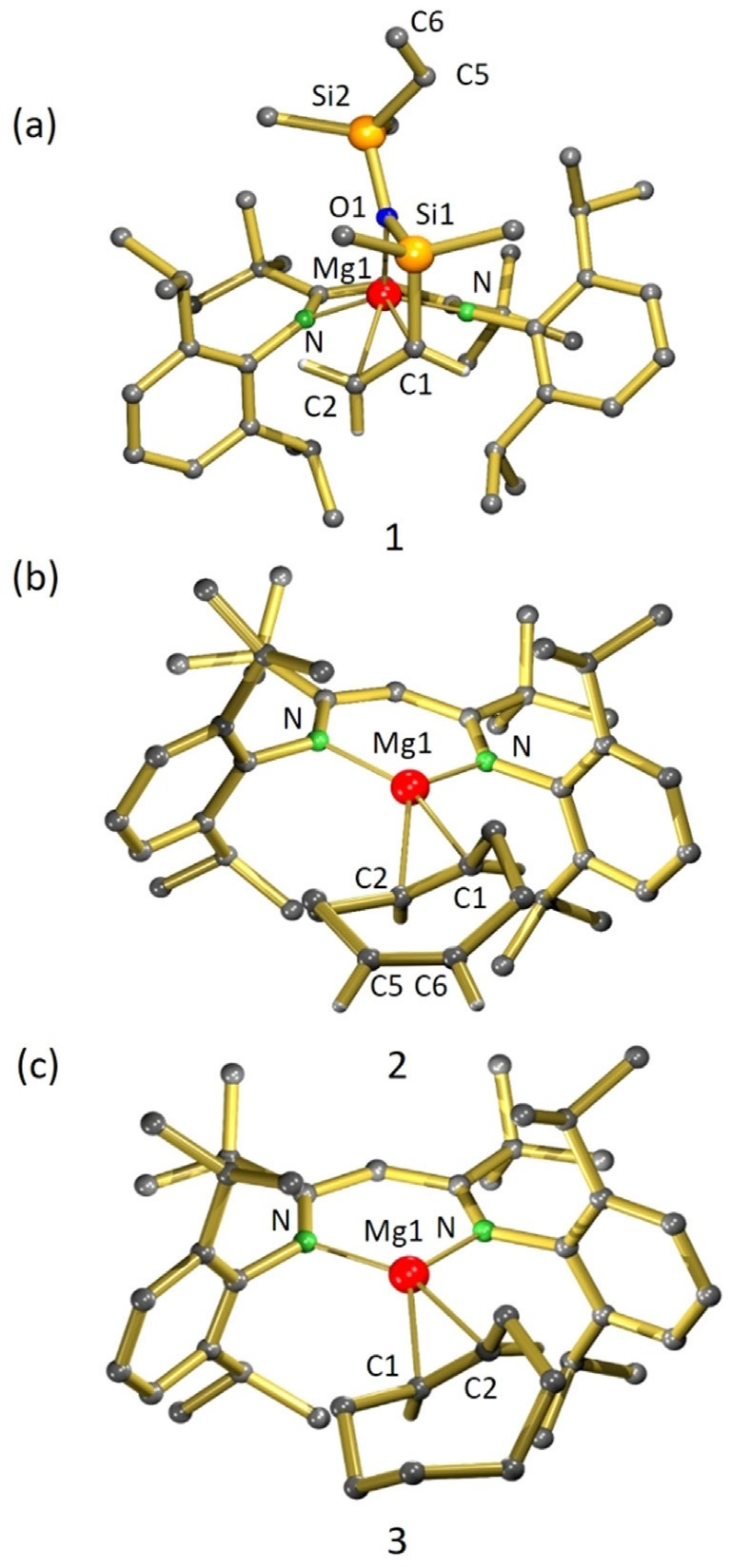
Crystal structures of (a) [(^*t*Bu^BDI)Mg^+^(divinylsiloxane)][B(C_6_F_5_)_4_^−^] (**1**), (b) [(^*t*Bu^BDI)Mg^+^(cod)][B(C_6_F_5_)_4_^−^] (**2**), and (c) [(^*t*Bu^BDI)Mg^+^(coe)][B(C_6_F_5_)_4_^−^] (**3**). The noncoordinating anion and the H atoms have been omitted for clarity, except for H atoms at double bonds.

**Figure 2:**
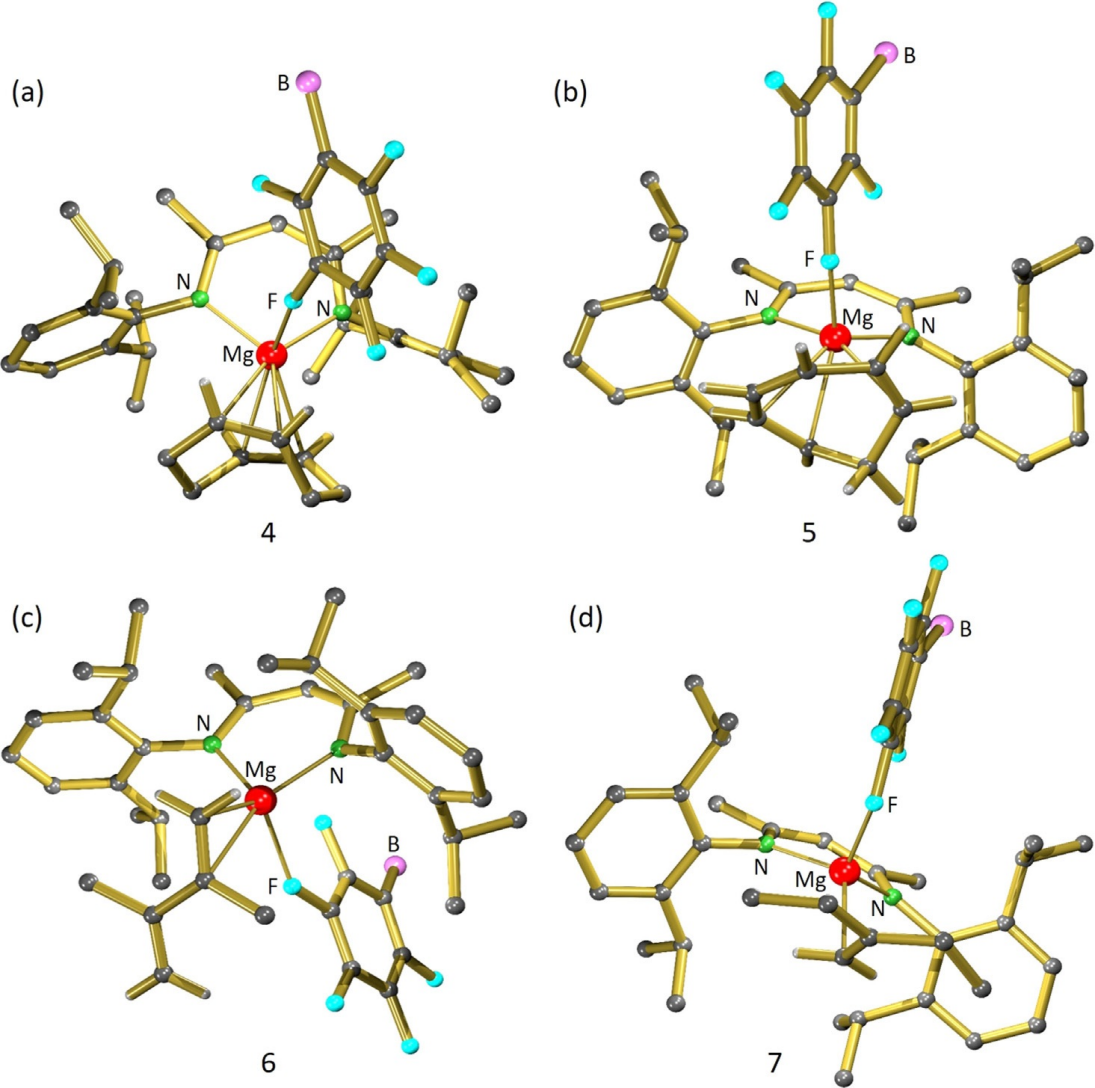
Crystal structures of a) [(^Me^BDI)Mg^+^(cod)][B(C_6_F_5_)_4_^−^] (**4**), b) [(^Me^BDI)Mg^+^(cht)][B(C_6_F_5_)_4_^−^] (**5**), c) [(^Me^BDI)Mg^+^(dmbd)][B(C_6_F_5_)_4_^−^] **6**), and (d) [(^Me^BDI)Mg^+^(eb)][B(C_6_F_5_)_4_^−^] (**7**). The monodentate coordinating anion is only partially shown and H atoms have been omitted for clarity, except for H atoms at double bonds.

Crystal structures of the (^*t*Bu^BDI)Mg^+^(alkene) and (^Me^BDI)Mg^+^(alkene) complexes are shown in Figures 1 and 2, respectively. The most important bond lengths and interactions are summarized in Table 1.

**Table 1:** Overview of selected bond lengths and interactions [Å] in the crystal structures of alkene complexes of the cations (^*t*Bu^BDI)Mg^+^ and (^Me^BDI)Mg^+^ in the form of their B(C_6_F_5_)_4_^−^ salts. Calculated values (ωB97XD/6‐31+G**) are given in brackets.

Complex	**1**	**2**	**3**	**4 a**	**4 b**	**5**	**6**	**7**
				Mg⋅⋅⋅F bond^[a]^	no Mg⋅⋅⋅F bond^[a]^			
Mg⋅⋅⋅C	2.539(2)–2.603(2)	2.437(2)–2.536(2)	2.415(3)–2.526(3)	2.488(2)–2.695(2)	2.491(3)–2.746(3)	2.601(2)–2.844(2)	2.365(1)–2.753(1)	2.338(2)–2.944(5)
	[2.570] [2.611]	[2.470–2.500]	[2.442–2.479]	[2.625–2.769]		[2.572–3.304]	[2.407–2.806]	[2.399–3.008]
C=C_coord._	1.330(3)	1.352(3)	1.327(3)	1.335(4)–1.357(4)	1.331(4)–1.354(3)	1.336(3)–1.351(3)	1.348(2)	1.328(5)
	[1.345]	[1.355]	[1.355]	[1.345–1.349]		[1.349–1.361]	[1.355]	[1.351]
C=C_uncoord._	1.321(3)	1.326(3)	–	–	–	1.358(3)	1.336(2)	–
	[1.338]	[1.336]				[1.359]	[1.340]	
Mg⋅⋅⋅F	–	–	–	2.454(2)–2.576(2)	–	2.154(1)	2.095(8)	2.106(1)
				[2.279]		[2.063]	[2.084]	[2.117]
Mg⋅⋅⋅O	2.049(1) [2.042]	–	–	–	–	–	–	–

[a] Five symmetry‐independent molecules are embedded in each unit cell. Three of those show anion‐cation interactions.

The following general and more specific observations can be made:

1. The Mg center in complexes with the bulkier β‐diketiminate ligand (^tBu^BDI) can only interact with one alkene bond. This is exemplified by the mono‐alkene coordination of divinylsiloxane and cod.
2. Complexes with the smaller β‐diketiminate ligand (^Me^BDI) are generally stabilized by one additional Mg⋅⋅⋅F interaction with the borate anion and can accommodate up to two alkenes in the Mg coordination sphere. This is illustrated by the crystal structures of the Mg–cod complex **4** and the Mg–cht complex **5**, in which the Mg center is too small to interact with all three alkenes. Note that in the latter the two remote alkene bonds in cht coordinate with Mg but one alkene is merely bound η^1^ with a long contact distance of 2.844(2) Å. Coordination of neighboring alkenes is apparently unfavorable, which explains that for dmbd only mono‐alkene instead of diene coordination is observed (the *cis* isomer of dmbd is only 2 kcal mol^−1^ higher in energy than its *trans* isomer).^[38]^ Also the recently isolated cationic Au complex [(*t*Bu_3_P)Au^+^(η^2^‐dmbd)][SbF_6_^−^] shows a planar *trans* conformation of dmbd and binding of the metal center to only one of the C=C bonds.^[39]^
3. In all cases, asymmetric Mg⋅⋅⋅alkene bonding is observed; that is, one of the Mg–C contacts is generally significantly shorter than the other. A similar preference for asymmetric alkene coordination was found in structures with intramolecular alkene coordination.^[24]^ Interestingly, in the crystal structure of [(^Me^BDI)Mg^+^(cod)][B(C_6_F_5_)_4_^−^] (**4**), the asymmetric unit contains five independent molecules with a wide range of different Mg⋅⋅⋅C interactions. There is not only a difference in alkene coordination but also in Mg⋅⋅⋅F bonding, which is absent in two of these molecules. This enormous fluctuation in a set of identical molecules originates only from crystal packing effects and clearly shows that the Mg–alkene bond should be considered weak and very dynamic.
4. The most extreme asymmetry in Mg–alkene coordination is found in complexes with the cations (^Me^BDI)Mg^+^(dmbd) (**6**, Mg⋅⋅⋅C 2.365(1)/2.753(1) Å) and (^Me^BDI)Mg^+^(eb) (**7**, Mg⋅⋅⋅C: 2.338(2)/2.944(5) Å). While in the latter, the longer Mg⋅⋅⋅C distance should be considered very weak, the shorter 2.338(2) Å contact is hitherto, to the best of our knowledge, the shortest Mg⋅⋅⋅C interaction observed to a neutral π ligand and even shorter than previously reported intramolecular Mg⋅⋅⋅C(alkene) interactions (range: 2.55–2.85 Å).[^19^, ^20^, ^24^, ^25^] It is also shorter than the shortest Mg⋅⋅⋅C contact in alkyne complex **III** ()2.399(2) Å or benzene adduct **IV** (2.367(2) Å).^[27]^ It is comparable to the Mg−C distance of 2.304(8) Å in Cp_2_Mg, which has a strong electrostatic interaction between Mg^2+^ and the Cp^−^ π ligands.^[40]^ DFT calculations confirm that strong coordination of Mg to the least alkylated olefin carbon atom is indeed most favorable (vide infra).
5. As noted previously, the metal–siloxane coordination in [(^*t*Bu^BDI)Mg^+^(divinylsiloxane)][B(C_6_F_5_)_4_^−^] (**1**) is rare and usually only observed in silacrown–metal complexes, in which metal complexation is facilitated by the cooperative effect of multiple metal–siloxane interactions.[^41^, ^42^] We reported the first metal–O(SiMe_3_)_2_ coordination in the form of a cationic Mg complex (**II**). The current divinylsiloxane complex **1** shows, apart from unusual Mg–alkene coordination, also a second example of intermolecular Mg–siloxane coordination. The somewhat longer Mg−O bond length of 2.049(1) Å in **1** compared with that of 1.993(1) Å in **II** is likely related to the bulkier β‐diketiminate ligand and concomitant alkene coordination. The Mg–O contact triggers polarization of electron density to O enforcing the Mg–O contact and diminishing the O→Si negative hyperconjugation typically found in silyl ethers.^[43]^ This is supported by the unusually acute Si‐O‐Si angle of 128.5(7)° [cf. 148.3(1)° in O(SiMe_3_)_2_] and the long Si−O bonds of 1.714(9) Å (average) [cf. 1.631(1) Å in O(SiMe_3_)_2_].^[44]^

### Solution studies

The crystal structures of the Mg–alkene complexes in the solid state are subject to crystal packing effects. The variety of different structures present in the unit cell of cod complex **4** already hints at highly dynamic solution behavior. All complexes dissolve in deuterated bromobenzene and were studied by NMR spectroscopy. Alkene coordination typically results in a shift of the alkene signals compared to the free alkenes. Significant broadening of the alkene signals, which was observed in some cases, indicates ligand exchange equilibria: (BDI)Mg^+^(alkene)⇄(BDI)Mg^+^+alkene.

Bromobenzene solutions of the Mg–alkene complexes with the bulkier ^*t*Bu^BDI ligand (**1**–**3**) all give ^1^H NMR spectra in which the alkene signals are sharp and coincide with those of the free alkenes. This demonstrates that these complexes are mainly dissociated in bromobenzene.

The Mg–alkene complexes with the smaller ^Me^BDI ligand are clearly more stable in bromobenzene solution. The cod ^1^H NMR signals in (**4**) (5.77 and 2.01 ppm) are shifted from those of free cod in C_6_D_5_Br (5.53 and 2.24 ppm). The relatively broad signals indicate an exchange equilibrium. On heating the sample to 55 °C sharper signals could be obtained and signals shifted towards those for free cod. For the cht complex **5**, similar observations have been made, and also this ligand is clearly partially bound in C_6_D_5_Br. In contrast, the signals of dmbd and eb in complexes **6** and **7**, which coincide with those of the free alkenes and are sharp, indicate dissociation.

### Theoretical considerations

Interactions between the β‐diketiminate Mg complexes and various alkenes were studied by DFT calculations (ωB97XD/6–311+G**//6‐31+G**, including corrections for dispersion by Grimme's D2 method). The Mg–alkene complexes **1**–**7** were fully optimized. In all cases the presence of the WCA B(C_6_F_5_)_4_^−^ was considered. Although the alkene ligands in these complexes are only loosely bound and subject to facile deformation by crystal packing effects, there is a surprisingly good match between crystal and calculated structures (see Table 1 for the most important bond lengths and interactions). Also the bond enthalpies (Table 2) and NPA charges (Table S3 in the Supporting Information) were calculated and Atoms in Molecules analyses (AIM) were performed. The following main conclusions can be drawn.

**Table 2:** Alkene complexation energies for [(^*t*Bu^BDI)Mg^+^][B(C_6_F_5_)_4_^−^] and [(^Me^BDI)Mg^+^][B(C_6_F_5_)_4_^−^]; ωB97XD/6–311+G**//6–31+G**. Δ*H* and Δ*G* contain corrections for zero‐point energy and entropy, respectively.

Alkene	Δ*E* [kcal mol^−^]	Δ*H* [kcal mol^−^]	Δ*G* (298 K, 1 atm) [kcal mol^−^]
[(^Me^BDI)Mg^+^][B(C_6_F_5_)_4_^−^]+alkene→(^Me^BDI)Mg^+^(alkene)][B(C_6_F_5_)_4_^−^]			
cod	−10.8	−5.9	+7.4
cht	−12.7	−7.8	−0.2
dmbd	−11.2	−7.0	+4.2
eb	−14.4	−10.2	−0.2
[(^*t*Bu^BDI)Mg^+^][B(C_6_F_5_)_4_^−^]+alkene→[(^*t*Bu^BDI)Mg^+^(alkene)][B(C_6_F_5_)_4_^−^]			
divinylsiloxane	−39.8	−37.1	−18.0
cod	−22.4	−22.1	−7.1
coe	−24.0	−24.3	−11.4

**Figure 3:**
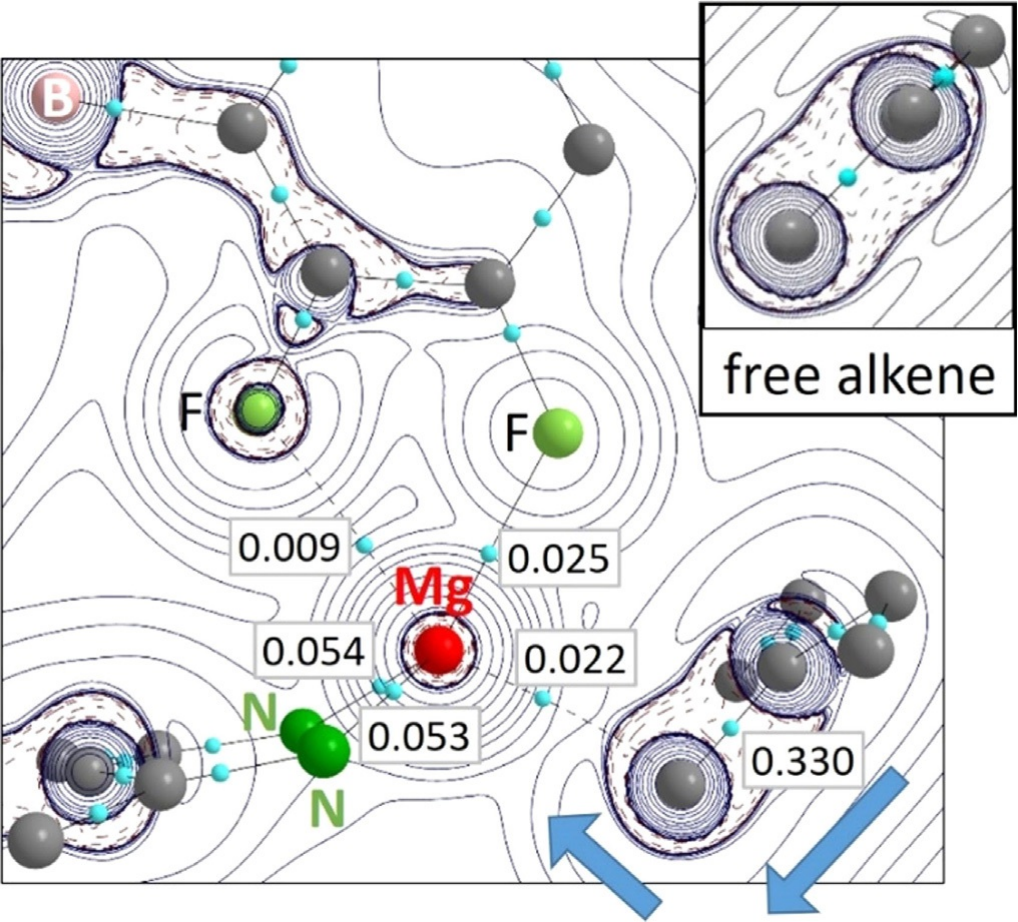
Laplacian of the electron density in the Mg/C=C plane of [(^Me^BDI)Mg^+^(eb)][B(C_6_F_5_)_4_^−^] (bond critical points are indicated by light blue spheres and boxed numbers relate to the electron density at these points (e B^−3^). Blue arrows show horizontal and vertical polarization of the electron density in the C=C bond compared to that in the free alkene (see inset). H atoms and DIPP groups are not shown for clarity.

1) Like in the crystal structures, the borate anion is not bound to Mg in complexes with the larger ^*t*Bu^BDI ligand (**1**–**3**). For **4**–**7**, that is, complexes with the smaller ^Me^BDI ligand, the borate anion is in all cases bound to Mg by a single Mg⋅⋅⋅(η^1^)B(C_6_F_5_)_4_ interaction. In agreement with experiment, the longest Mg⋅⋅⋅F contacts are found in the Mg–cod complex **4**, while Mg–cht complex **5** features an exceptionally short interaction.

2) The experimentally observed Mg–alkene coordination modes are fully reproduced by the calculations. For example, the diene cod is chelating in the complex with the ^Me^BDI ligand but only one alkene is bound in the complex with the bulkier (^*t*Bu^BDI) ligand. The triene cht in **5** binds Mg with one short Mg–η^2^‐alkene interaction (2.601(2)/2.692(2) Å), while the second interaction should be categorized as an Mg–η^1^‐alkene bond (2.844(2) Å). The diene dmbd in **6** can only bind Mg with one of its double bonds.

3) In all cases asymmetric Mg–alkene bonding is observed. The most extreme cases of asymmetric Mg–alkene bonding are found in the complexes with dmbd and eb ligands (**6** and **7**) and, although these ligands are only loosely bound, the experimental solid‐state values are reproduced surprisingly well by these gas‐phase calculations. This important observation means that the geometries of these complexes are mainly influenced by intramolecular forces and much less by intermolecular packing effects.

4) The C=C bonds are in all cases hardly elongated by Mg–alkene coordination. Compared with the free alkenes generally an elongation of 0.01–0.02 Å is observed.

5) Considering the complexation energies (Table 2) it is striking that alkene bonding to the more sterically congested [(^*t*Bu^BDI)Mg^+^][B(C_6_F_5_)_4_^−^] is significantly more exothermic than that to [(^Me^BDI)Mg^+^][B(C_6_F_5_)_4_^−^]. Although this seems counterintuitive, it may be explained by the more facile cation–anion dissociation in the sterically congested complex. However, overestimation of the dispersion correction could also contribute to this phenomenon. In the (^Me^BDI)Mg^+^ series of complexes, coordination of eb is more exothermic than chelating cod complexation. Although this may seem surprising, it should be considered that the shortest Mg–C contact to cod (2.625 Å) is longer than that to eb (2.399 Å). Also the large asymmetry in the Mg–eb bond and the concomitant C=C bond polarization (vide infra) contribute positively to the bond energy. Comparison of Δ*H* and Δ*G* values shows especially for chelating ligands such as cod or divinylsiloxane large changes, signifying the considerable loss in entropy for bidentate coordination. It is noteworthy that alkene coordination can be more favorable than benzene coordination. Energies [kcal mol^−1^] for formation of the benzene complex [(^Me^BDI)Mg(benzene)^+^][B(C_6_F_5_)_4_^−^]: Δ*E* −6.8, Δ*H*=−5.9, Δ*G*=+5.6.

6) The calculated NPA charges show that bonding in the (BDI)Mg^+^ cations is mainly electrostatic (the charges on the Mg centers vary from +1.81 to +1.86 and those on the BDI ligands from −0.89 to −0.94). There is hardly any electron transfer from the alkene to the Mg^2+^ cation: charges on the alkene ligands in the complexes vary from +0.02 to +0.05. This is in agreement with a predominantly electrostatic interaction between a highly positively charged Mg^2+^ cation and a polarized alkene bond. AIM analyses show that the C=C bond in the alkene ligands is only weakly affected by Mg coordination, as is evident from minor changes in electron densities in the C−C bond critical points, bond ellipticities, and delocalization indices (Table S4, Figures S55–61 in the Supporting Information).

7) Earlier AIM studies have shown polarization of the C=C π‐electron density towards the C atom closest to the metal atom (horizontal polarization) and towards the metal atom itself (vertical polarization).[^24^, ^25^] The Mg–eb bond in **7** is an extreme case of asymmetric Mg–alkene bonding. AIM analysis of this complex shows tremendous horizontal and vertical polarization of the π‐electron density (Figure 3). Strong horizontal polarization of Mg‐bound H_2_C=CEt_2_ is supported by the NPA charges on CH_2_ (−0.70) and CEt_2_ (+0.68) groups [ cf. free eb: CH_2_ (−0.43) and CEt_2_ (+0.43)]. Although the AIM parameters of the C=C bond are generally hardly affected by Mg–alkene coordination, Mg–eb coordination has a pronounced effect on the C=C bond ellipticities and delocalization indices (Table S4 in the Supporting Information).

## Conclusion

The first crystal structures of unsupported magnesium alkene complexes have been presented. Complexation of neutral, nonpolar alkenes to Mg was accomplished by exploiting the very high Lewis acidity of the unsolvated β‐diketiminate Mg cation (BDI)Mg^+^. Since Mg–solvent interactions compete with Mg–alkene bonding, weakly coordinating chlorobenzene was used as solvent. Also (BDI)Mg⋅⋅⋅(C_6_F_5_)_4_B^−^ interactions may hinder alkene coordination, but these cation–anion interactions can be minimized by choosing a bulky BDI ligand, such as ^*t*Bu^BDI, that still leaves room for alkene coordination.

Crystal structures of a variety of Mg–alkene complexes show that the bulkier (^*t*Bu^BDI)Mg^+^ cation can only bind to one C=C bond, while the more accessible (^Me^BDI)Mg^+^ cation binds the diene cod in a chelating fashion. In all cases, asymmetric Mg–alkene bonding is observed. Although it may be expected that such weak Mg⋅⋅⋅alkene interactions are prone to distortion by crystal packing effects, this is not the case. Calculated gas‐phase structures reproduce the experimental solid‐state structures quite well. The geometry of Mg–alkene coordination is therefore mainly determined by intramolecular steric and electronic factors. Since the π‐electron density in Et_2_C=CH_2_ (eb) is strongly polarized towards the terminal CH_2_ group by the σ‐electron‐donating Et substituents, this alkene shows highly asymmetric bonding. The complex could be regarded as having partial (^Me^BDI)Mg‐CH_2_‐(Et)_2_C^+^ character. All Mg–alkene bonds are quite labile and, when dissolved in deuterated bromobenzene, the complexes are either completely dissociated or in a dissociation equilibrium.

DFT calculations on the full complexes, that is, including the weakly coordinating (C_6_F_5_)_4_B^−^ anions, show that alkenes form stronger bonds to the bulkier (^*t*Bu^BDI)Mg^+^ cation than to (^Me^BDI)Mg^+^. This is likely related to the fact that the smaller cation forms a tighter cation–anion pair. This conclusion does not agree with solution studies, which show complete dissociation for alkene complexes with the bulkier ^*t*Bu^BDI ligand, while those with the ^Me^BDI ligand could be detected. This discrepancy between gas‐phase calculations and solution studies must be related to competition with solvent coordination. On coordination, the C=C bonds are hardly elongated, and also insignificant electron transfer to the metal is observed. The Mg–alkene bonds are therefore mainly electrostatic and should be described as Mg^2+^ ion‐induced dipole interactions, which are especially strong for asymmetric alkene coordination. Although much weaker than transition metal–alkene bonding, these first examples of unsupported s‐block metal– alkene bonds clearly show that these interactions can also be of significance in early main group chemistry, especially in the related field of catalysis.

## Experimental Section

### General experimental procedures

All experiments were conducted under an inert nitrogen atmosphere by using standard Schlenk and glovebox techniques (MBraun, Labmaster SP). *n*‐Hexane and *n*‐pentane were degassed with nitrogen, dried over activated aluminum oxide (Solvent Purification System: Pure Solv 400‐4‐MD, Innovative Technology), and stored over 3 Å molecular sieves. Chlorobenzene, bromobenzene, and methylcyclohexane were dried over calcium hydride, distilled under N_2_ atmosphere, and stored over 3 Å molecular sieves. C_6_D_5_Br (99.6 % D, Sigma‐Aldrich) was dried over 3 Å molecular sieves. 1,5‐Cyclooctadiene (cod, Acros Organics, 99+%), cyclooctene (coe, ABCR, 95 %), 1,3,5‐cycloheptatriene (cht, Sigma‐Aldrich, 95 %), 2,3‐dimethylbuta‐1,3‐diene (dmbd, Alfa Aesar, 98 %), 2‐ethyl‐1‐butene (eb, TCI, 97 %), and 1,3‐divinyltetramethyldisiloxane (divinylsiloxane, TCI, 98 %) were dried over calcium hydride, distilled under N_2_ atmosphere, and stored over 3 Å molecular sieves. [Ph_3_C^+^][B(C_6_F_5_)_4_^−^] (Boulder Scientific) was used as received. [CH[C(Me)N‐DIPP]_2_ Mg*n*Bu]_2_[(^Me^BDI)Mg*n*Bu]_2_,^[45]^ and CH[C(tBu)N‐DIPP]_2_Mg*n*Bu (^*t*Bu^BDI)Mg*n*Bu^[45]^ were synthesized according to literature procedures. [(^Me^BDI)Mg^+^][B(C_6_F_5_)_4_^−^] was synthesized according to an adapted literature procedure by using [(^Me^BDI)Mg*n*Bu]_2_ instead of [(^Me^BDI)Mg*n*Pr]_2_.^[27]^ NMR spectra were recorded with a Bruker Avance III HD 400 MHz or a Bruker Avance III HD 600 MHz spectrometer. The spectra were referenced to the respective residual signals of the deuterated solvents.^[46]^ Elemental analysis was performed with a Euro EA 3000 (Euro Vector) analyzer. All crystal data were measured with a SuperNova (Agilent) diffractometer with dual Cu and Mo microfocus sources and an Atlas or Atlas S2 detector. Deposition Numbers 2040108 (for **1**), 2040109 (for **2**), 2040110 (for **3**), 2040111 (for **4**), 2040112 (for **5**), 2040113 (for **6**), and 2040114 (for **7**) contain the supplementary crystallographic data for this paper. These data are provided free of charge by the joint Cambridge Crystallographic Data Centre and Fachinformationszentrum Karlsruhe Access Structures service.

### Synthesis

**Synthesis of [(** ^***t*****Bu**^ **BDI)Mg^+^(divinylsiloxane)][B(C_6_F_5_)_4_** ^**−**^ **] (1)**: (^*t*Bu^BDI)Mg*n*Bu (0.0498 g, 0.0854 mmol) and [Ph_3_C^+^][B(C_6_F_5_)_4_^−^] (0.0719 g, 0.0780 mmol) were dissolved in chlorobenzene (2 mL). After 1 min of stirring, a color change from reddish brown to pale yellow was observed. The solution was layered with a large excess of divinylsiloxane (1 mL) at ambient temperature. After 1 d colorless crystals had formed. After decantation, washing with *n*‐hexane (2×0.5 mL) and briefly drying under vacuum, the crystalline product was obtained in 41 % yield (0.0451 g, 0.0321 mmol). ^1^H NMR (C_6_D_5_Br, 600 MHz, 298 K): *δ*=7.10–7.08 (m, 2 H, Ar*H*), 6.95–6.93 (br m, 4 H, Ar*H*), 6.17–6.13 (br m, 2 H, (CH_3_)_2_Si(CH=CH_2_)_2_), 5.92 (dd, *vicinal cis* ^3^ *J*_HH_=14.8 Hz, *geminal* ^3^ *J*_HH_=3.8 Hz, 2 H, (CH_3_)_2_Si(CH=CH_2_)_2_), 5.75 (dd, *vicinal trans* ^3^ *J*_HH_=20.4 Hz, *geminal* ^3^ *J*_HH_=3.8 Hz, 2 H, (CH_3_)_2_Si(CH=CH_2_)_2_), 5.51 (s, 1 H, CC*H*C), 2.89 (br hept, 4 H, C*H*Me_2_), 1.17 (d, ^3^ *J*_HH_=6.7 Hz, 12 H, CH(C*H*_3_)_2_), 1.03 (s, 18 H, C(C*H*_3_)_3_), 0.87–0.81 (m, 12 H, CH(C*H*_3_)_2_), 0.17 ppm (s, 12 H, (C*H*_3_)_2_Si(CH=CH_2_)_2_). ^13^C{^1^H} NMR (C_6_D_5_Br, 151 MHz, 298 K): *δ*=181.7 (s, N*C*(CH_3_)_3_), 149.1 (br d, ^1^ *J*_CF_=244 Hz, B(*C*_6_F_5_)_4_), 143.1 (s, Ar*C*), 141.6 (s, Ar*C*), 140.0 (s, (CH_3_)_2_Si(*CH=C*H_2_)_2_), 138.8 (br d, ^1^ *J*_CF_=244 Hz, B(*C*_6_F_5_)_4_), 137.2 (br d, ^1^ *J*_CF_=244 Hz, B(*C*_6_F_5_)_4_), 132.1 (s, (CH_3_)_2_Si(*CH=C*H_2_)_2_), 125.1 (s, Ar*C*H), 121.3 (s, Ar*C*H), 97.8 (s, C*C*HC), 45.2 (s, *C*(CH_3_)_3_), 32.7 (s, C(*C*H_3_)_3_), 28.6 (s, *C*H(CH_3_)_2_), 26.2 (s, CH(*C*H_3_)_2_), 23.9 (s, CH(*C*H_3_)_2_), 1.0 ppm (s, (*C*H_3_)_2_Si(CH=CH_2_)_2_). Signals for vinyl groups and C*C*HC can only be assigned by HSQC, signal for N*C*(CH_3_)_3_ can only be assigned by HMBC, B‐*C* of B(C_6_F_5_)_4_ was not detected. ^19^F{^1^H} NMR (C_6_D_5_Br, 565 MHz, 298 K): *δ*=−130.4 (br s, 8 F, *o*‐C*F*), −160.9 (t, ^3^ *J_FF_*=21 Hz, 4 F, *p*‐C*F*), −165.8 ppm (br s, 8 F, *m*‐C*F*). ^11^B{^1^H} NMR (C_6_D_5_Br, 193 MHz, 298 K): δ−15.7 (s, *B*(C_6_F_5_)_4_) ppm. IR (ATR, neat): $\tilde{\nu }\;$ =2968 (m), 2901 (w), 2878 (w), 1642 (m), 1512 (m), 1459 (vs.), 1406 (s), 1383 (m), 1359 (m), 1310 (m), 1266 (m), 1253 (m), 1107 (s), 978 (vs.), 842 (m), 801 (m), 755 (m), 725 (m), 660 (m), 574 cm^−1^ (m). Elemental analysis calcd (%) for C_67_H_71_BF_20_MgN_2_OSi_2_ (*M*=1406.60 g mol^−1^): C, 57.83; H, 5.14; N, 2.01; found: C, 57.16; H, 5.05; N, 1.79.

**Synthesis of [(** ^***t*****Bu**^ **BDI)Mg^+^(cod)][B(C_6_F_5_)_4_** ^**−**^ **] (2)**: (^*t*Bu^BDI)Mg*n*Bu (0.0300 g, 0.0514 mmol) and [Ph_3_C^+^][B(C_6_F_5_)_4_^−^] (0.0452 g, 0.0490 mmol) were dissolved in chlorobenzene (0.5 mL). After 1 min of stirring, a color change from orange brown to pale yellow was observed, and subsequently a large excess of cod (0.1 mL) was slowly added at room temperature. The microcrystalline solid that formed overnight was isolated by decantation, washed with *n*‐hexane (6×0.5 mL), and briefly dried under vacuum to give the product in a yield of 70 % (0.0450 g, 0.0343 mmol). Crystals suitable for X‐ray diffraction were grown from bromobenzene. ^1^H NMR (C_6_D_5_Br, 600 MHz, 298 K): *δ*=7.09 (t, ^3^ *J*_HH_=7.7 Hz, 2 H, Ar*H*), 6.94 (br d, 4 H, Ar*H*), 5.53 (br s, 4 H, C*H (*cod)), 5.51 (s, 1 H, CC*H*C), 2.90 (hept, ^3^ *J_HH_*=6.8 Hz, 4 H, C*H*Me_2_), 2.24 (br s, 8 H, C*H_2_* (cod)), 1.17 (d, ^3^ *J*_HH_=6.8 Hz, 12 H, CH(C*H*_3_)_2_), 1.04 (s, 18 H, C(C*H*_3_)_3_), 0.84 ppm (d, ^3^ *J*_HH_=6.8 Hz, 12 H, CH(C*H*_3_)_2_). ^13^C{^1^H} NMR (C_6_D_5_Br, 151 MHz, 298 K): *δ*=181.6 (s, N*C*(CH_3_)_3_), 148.7 (br d, ^1^ *J*_CF_=244 Hz, B(*C*_6_F_5_)_4_), 142.6 (s, Ar*C*), 141.3 (s, Ar*C*), 138.6 (br d, ^1^ *J*_CF_=244 Hz, B(*C*_6_F_5_)_4_), 137.0 (br d, ^1^ *J*_CF_=244 Hz, B(*C*_6_F_5_)_4_), 128.9 (s, *C*H (cod)), 126.9 (s, Ar*C*H), 124.8 (s, Ar*C*H), 97.3 (s, C*C*HC), 44.9 (s, *C*(CH_3_)_3_), 32.4 (s, C(*C*H_3_)_3_), 28.4 (s, *C*H_2_ (cod)), 28.4 (s, *C*H(CH_3_)_2_), 25.8 (s, CH(*C*H_3_)_2_), 23.5 (s, CH(*C*H_3_)_2_) ppm. Signal for B‐*C* of B(*C_6_*F_5_)_4_ was not detected. ^19^F{^1^H} NMR (C_6_D_5_Br, 565 MHz, 298 K): *δ*=−130.7 (br s, 8 F, *o*‐C*F*), −161.2 (br t, 4F, *p*‐C*F*), −166.2 ppm (br s, 8 F, *m*‐C*F*). ^11^B{^1^H} NMR (C_6_D_5_Br, 193 MHz, 298 K): *δ*=−16.0 (s, *B*(C_6_F_5_)_4_) ppm. IR (ATR, neat): $\tilde{\nu }\;$ =2969 (m), 2875 (w), 1640 (m), 1510 (m), 1457 (vs.), 1379 (m), 1357 (s), 1316 (m), 1275 (m), 1218 (m), 1196 (w), 1081 (s), 977 (vs.), 808 (w), 773 (m), 755 (m), 716 (w), 683 (m), 659 cm^−1^ (m). Elemental analysis calcd (%) for C_67_H_65_BF_20_MgN_2_ (*M*=1313.15 g mol^−1^): C, 61.27; H, 4.99; N, 2.13; found: C, 61.26; H, 5.12; N, 2.63.

**Synthesis of [(** ^***t*****Bu**^ **BDI)Mg^+^(coe)][B(C_6_F_5_)_4_** ^**−**^ **] (3)**: (^*t*Bu^BDI)Mg*n*Bu (0.0300 g, 0.0514 mmol) and [Ph_3_C^+^][B(C_6_F_5_)_4_^−^] (0.0452 g, 0.0490 mmol) were dissolved in chlorobenzene (0.5 mL). Stirring the reaction mixture for 1 min resulted in a color change from orange‐brown to pale yellow. Subsequently the reaction mixture was layered with a large excess of coe (0.1 mL) at room temperature. Layering with *n*‐hexane (1 mL) gave colorless crystals, which after decantation were washed with *n*‐hexane (3×2 mL) and briefly dried under vacuum to give the product in a yield of 74 % (0.0480 g, 0.0365 mmol). ^1^H NMR (C_6_D_5_Br, 600 MHz, 298 K): *δ*=7.09 (t, ^3^ *J*_HH_=7.7 Hz, 2 H, Ar*H*), 6.94 (br d, 4 H, Ar*H*), 5.60 (m, 2 H, C*H* (coe)), 5.51 (s, 1 H, CC*H*C), 2.89 (hept, ^3^ *J*_HH_=6.8 Hz, 4 H, C*H*Me_2_), 2.04 (br s, 4 H, C*H_2_* (coe)), 1.42 (br s, 8 H, C*H_2_* (coe)), 1.17 (d, ^3^ *J*_HH_=6.8 Hz, 12 H, CH(C*H*_3_)_2_), 1.04 (s, 18 H, C(C*H*_3_)_3_), 0.84 (d, ^3^ *J*_HH_=6.8 Hz, 12 H, CH(C*H*_3_)_2_) ppm. ^13^C{^1^H} NMR (C_6_D_5_Br, 151 MHz, 298 K): *δ*=181.7 (s, N*C*(CH_3_)_3_), 148.7 (br d, ^1^ *J*_CF_=244 Hz, B(*C*_6_F_5_)_4_), 142.6 (s, Ar*C*), 141.3 (s, Ar*C*), 138.6 (br d, ^1^ *J*_CF_=244 Hz, B(*C*_6_F_5_)_4_), 137.0 (br d, ^1^ *J*_CF_=244 Hz, B(*C*_6_F_5_)_4_), 126.8 (s, Ar*C*H), 124.8 (s, Ar*C*H), 97.4 (s, C*C*HC), 44.9 (s, *C*(CH_3_)_3_), 32.4 (s, C(*C*H_3_)_3_), 29.6 (s, *C*H_2_ (coe)), 28.4 (s, *C*H(CH_3_)_2_), 26.4 (s, *C*H_2_ (coe)), 25.8 (s, CH(*C*H_3_)_2_), 25.8 (s, *C*H_2_ (coe)), 23.5 ppm (s, CH(*C*H_3_)_2_). Signals for B‐*C* of B(*C_6_*F_5_)_4_ and coe C=CH were not detected. ^19^F{^1^H} NMR (C_6_D_5_Br, 565 MHz, 298 K): *δ*=−130.8 (br s, 8 F, *o*‐C*F*), −161.2 (br t, 4 F, *p*‐C*F*), −166.1 ppm (br s, 8 F, *m*‐C*F*). ^11^B{^1^H} NMR (C_6_D_5_Br, 193 MHz, 298 K): *δ*=−16.0 (s, *B*(C_6_F_5_)_4_) ppm. IR (ATR, neat): $\tilde{\nu }\;$ =2964 (m), 2936 (w), 2871 (w), 1640 (m), 1510 (m), 1457 (vs.), 1379 (m), 1355 (vs.), 1316 (m), 1275 (m), 1218 (w), 1081 (s), 979 (vs.), 906 (w), 801 (m), 775 (m), 755 (m), 683 (m), 659 cm^−1^ (m). Elemental analysis calcd (%) for C_67_H_67_BF_20_MgN_2_ (*M*=1315.37 g mol^−1^): C, 61.18; H, 5.13; N, 2.13; found: C, 61.20; H, 5.36; N, 1.90.

**Synthesis of [(^Me^BDI)Mg^+^(cod)][B(C_6_F_5_)_4_** ^**−**^ **] (4)**: [(^Me^BDI)Mg*n*Bu]_2_ (0.0601 g, 0.0602 mmol) and [Ph_3_C^+^][B(C_6_F_5_)_4_^−^] (0.0871 g, 0.0944 mmol) were dissolved in chlorobenzene (0.5 mL) and stirred until the solution became almost colorless (1 min). After filtration, cod (0.2 mL) was added, and the reaction mixture was left at room temperature for 2 d. *n*‐Hexane (1 mL) was added and the glass wall of the vial was scratched with a spatula to initiate crystallization. The crystalline product was washed with *n*‐hexane (4×0.6 mL), briefly dried under vacuum, and isolated in a yield of 62 % (0.0721 g, 0.0587 mmol). ^1^H NMR (600 MHz, C_6_D_5_Br, 298 K): *δ*=7.19–7.14 (m, 3 H, Ar*H*), 7.07–7.05 (m, 3 H, Ar*H*), 5.77 (br s, 4 H, C*H (*cod)), 4.91 (s, 1 H, CC*H*C), 2.76 (hept, ^3^ *J*_HH_=6.6 Hz, 4 H, C*H*Me_2_), 2.01 (br s, 8 H, C*H_2_* (cod)), 1.59 (s, 6 H, CC*H_3_*), 1.04 ppm (d, ^3^ *J*_HH_=6.6 Hz, 24 H, CH(C*H*_3_)_2_). ^13^C{^1^H} NMR (151 MHz, C_6_D_5_Br, 328 K): *δ*=173.2 (s, N*C*(CH_3_)), 148.9 (br d, ^1^ *J*_CF_=231 Hz, B(*C_6_*F_5_)_4_), 142.1 (s, Ar*C*), 141.9 (s, Ar*C*), 138.7 (br d, ^1^ *J*_CF_=231 Hz, B(*C_6_*F_5_)_4_), 136.7 (br d, ^1^ *J*_CF_=244 Hz, B(*C_6_*F_5_)_4_), 128.8 (s, Ar*C*), 127.6 (s, Ar*C*H), 126.6 (s, Ar*C*H), 124.9 (s, Ar*C*H), 96.9 (s, C*C*HC), 29.0 (s, *C*HMe_2_), 27.7 (s, *C*H_2_ (cod)), 24.7 (s, CH*C*H_3_), 24.5 (s, NC(*C*H_3_)), 23.9 ppm (s, CH*C*H_3_). The signals for *C*H of cod and B‐*C* of B(*C_6_*F_5_)_4_) were not detected_._^19^F{^1^H} NMR (565 MHz, C_6_D_5_Br, 298 K): *δ*=−131.2 (s, 8 F, *o*‐C*F*), −160.4 (t, ^3^ *J*_FF_=21 Hz, 4 F, *p*‐C*F*), −165.0 ppm (s, 8 F, *m*‐C*F*). ^11^B{^1^H} NMR (128 MHz, C_6_D_5_Br, 298 K): *δ*=−15.6 (s, *B*(C_6_F_5_)_4_) ppm. IR (ATR, neat): $\tilde{\nu }$ =2966 (m), 2935 (w), 2872 (w), 1641 (m), 1513 (m), 1459 (vs.), 1388 (s), 1307 (m), 1263 (s), 1085 (s), 931 (vs.), 856 (w), 755 (w), 725 (m), 684 (m), 661 cm^−1^ (m). Elemental analysis calcd (%) for C_61_H_53_N_2_BF_20_Mg (*M*=1229.19 g mol^−1^): C, 59.61; H, 4.35; N, 2.28; found: C, 59.90; H, 4.33; N, 1.89.

**Synthesis of [(^Me^BDI)Mg^+^(cht)][B(C_6_F_5_)_4_** ^**−**^ **] (5)**: [(^Me^BDI)Mg*n*Bu]_2_ (0.0521 g, 0.0522 mmol) and [Ph_3_C^+^][B(C_6_F_5_)_4_^−^] (0.0790 g, 0.0857 mmol) were dissolved in chlorobenzene (0.3 mL) and stirred until the solution became almost colorless (1 min). After filtration, a mixture of cht (42 μL, 5 equiv.) and methylcyclohexane (0.3 mL) was added and the reaction mixture was left at room temperature for 1 d. Adding 0.9 mL of *n*‐hexane at room temperature initiated crystallization. The crystalline product was washed with *n*‐pentane (4×0.2 mL), briefly dried under vacuum, and isolated in a yield of 52 % (0.0532 g, 0.0433 mmol). ^1^H NMR (400 MHz, C_6_D_5_Br_,_ 298 K): *δ*=7.20–7.14 (m, 4 H, Ar*H*), 7.06–7.04 (m, 2 H, Ar*H*), 6.46 (br s, 2 H, C*H* (cht)), 6.09 (d, ^3^ *J*_HH_=6.6 Hz, 2 H, C*H* (cht)), 5.27–5.21 (m, 2 H, C*H* (cht)), 4.92 (s, 1 H, CC*H*C), 2.73 (hept, ^3^ *J*_HH_=6.6 Hz, 4 H, C*H*Me_2_), 2.07 (br s, 2 H, C*H_2_* (cht)), 1.57 (s, 6 H, CC*H_3_*), 1.04 (d, ^3^ *J*_HH_=6.6 Hz, 12 H, CH(C*H*_3_)_2_), 0.94 (d, ^3^ *J*_HH_=6.6 Hz, 12 H, CH(C*H*_3_)_2_) ppm. ^13^C{^1^H} NMR (101 MHz, C_6_D_5_Br, 298 K): *δ*=173.2 (s, N*C*(CH_3_)), 149.2 (br d, ^1^ *J*_CF_=240 Hz, B(*C_6_*F_5_)_4_), 142.0 (s, Ar*C*), 138.6 (br d, ^1^ *J*_CF_=240 Hz, B(*C_6_*F_5_)_4_), 137.1 (br d, ^1^ *J*_CF_=240 Hz, B(*C_6_*F_5_)_4_), 128.8 (s, Ar*C*), 127.4 (s, Ar*C*H), 124.8 (s, Ar*C*H), 96.8 (s, C*C*HC), 35.7 (*C*H_2_ (cht)), 28.8 (s, *C*HMe_2_), 28.2 (s, CH*C*H_3_), 24.4 (s, NC(*C*H_3_)), 24.1 (s, CH*C*H_3_) ppm. The olefinic signals for cht and B‐*C* of B(*C_6_*F_5_)_4_ were not detected. ^19^F{^1^H} NMR (376 MHz, C_6_D_5_Br, 298 K): *δ*=−131.3 (br s, 8 F, *o*‐C*F*), −159.9 (t, ^3^ *J*_FF_=21 Hz, 4 F, *p*‐C*F*), −164.9 ppm (t, ^3^ *J*_FF_=21 Hz, 8 F, *m*‐C*F*). ^11^B{^1^H} NMR (128 MHz, C_6_D_5_Br, 298 K): *δ*=−15.7 ppm (s, *B*(C_6_F_5_)_4_). IR (ATR, neat): $\tilde{\nu \;}$ =2967 (m), 2939 (w), 2878 (w), 1643 (m), 1534 (m), 1460 (vs), 1372 (s), 1327 (m), 1290 (s), 1084 (s), 989 (vs), 858 (w), 796 (w), 755 (m), 712 (m), 660 cm^−1^ (m). Elemental analysis calcd (%) for C_61_H_53_N_2_BF_20_Mg (*M*=1229.19 g mol^−1^): C, 59.40; H, 4.07; N, 2.31; found: C, 59.10; H, 4.53; N, 2.00.

**Synthesis of [(^Me^BDI)Mg^+^(dmbd)][B(C_6_F_5_)_4_** ^**−**^ **] (6)**: [(^Me^BDI)Mg*n*Bu]_2_ (0.0389 g, 0.0390 mmol) and [Ph_3_C^+^][B(C_6_F_5_)_4_^−^] (0.0582 g, 0.0631 mmol) were dissolved in chlorobenzene (0.2 mL) and stirred until the solution became almost colorless (1 min). After filtration, dmbd (0.2 mL) was added and the reaction mixture left at −20 °C for 2 d. Scratching the glass wall of the vial with a spatula initiated crystallization. The crystalline product was washed with *n*‐hexane (3×0.2 mL), briefly dried under vacuum, and isolated in a yield of 58 % (0.0441 g, 0.0367 mmol). ^1^H NMR (600 MHz, C_6_D_5_Br_,_ 298 K): *δ*=7.20–7.14 (m, 3 H, Ar*H*), 7.06–7.04 (m, 3 H, Ar*H*), 5.02 (s, 2 H, C*H_2_* (dmbd)), 4.95 (s, 1 H, CC*H*C), 4.92 (s, 2 H, C*H_2_* (dmbd)), 2.76 (hept, ^3^ *J*_HH_=6.7 Hz, 4 H, C*H*Me_2_), 1.83 (s, 6 H, C*H_3_* (dmbd)), 1.58 (s, 6 H, CC*H_3_*), 1.06 (d, ^3^ *J*_HH_=6.7 Hz, 12 H, CH(C*H*_3_)_2_), 0.89 (d, ^3^ *J*_HH_=6.7 Hz, 12 H, CH(C*H*_3_)_2_) ppm. ^13^C{^1^H} NMR (151 MHz, C_6_D_5_Br, 298 K): *δ*=173.4 (s, N*C*(CH_3_)), 148.8 (br d, ^1^ *J*_CF_=242 Hz, B(*C_6_*F_5_)_4_), 143.4 (s, *C* (dmbd)), 142.1 (s, Ar*C*), 141.5 (s, Ar*C*), 138.6 (br d, ^1^ *J*_CF_=242 Hz, B(*C_6_*F_5_)_4_), 137.2 (br d, ^1^ *J*_CF_=242 Hz, B(*C_6_*F_5_)_4_), 128.8 (s, Ar*C*), 127.3 (s, Ar*C*H), 126.7 (s, Ar*C*H), 124.8 (s, Ar*C*H), 113.6 (s, *C*H_2_ (dmbd)), 96.9 (s, C*C*HC), 28.8 (s, *C*HMe_2_), 24.3 (s, CH*C*H_3_), 24.2 (s, NC(*C*H_3_)), 24.1 (s, CH*C*H_3_), 21.0 ppm (s, *C*H_3_ (dmbd)). B‐*C* of B(*C_6_*F_5_)_4_ was not detected. ^19^F{^1^H} NMR (376 MHz, C_6_D_5_Br, 298 K): *δ*=−131.5 (s, 8 F, *o*‐C*F*), −159.5 (t, ^3^ *J*_FF_=21 Hz, 4 F, *p*‐C*F*), −164.6 ppm (s, 8 F, *m*‐C*F*). ^11^B{^1^H} NMR (128 MHz, C_6_D_5_Br, 298 K): *δ*=−15.7 (s, *B*(C_6_F_5_)_4_) ppm. IR (ATR, neat): $\tilde{\nu }\;$ =2968 (m), 2930 (w), 2868 (w), 1642 (m), 1512 (m), 1459 (vs.), 1372 (s), 1309 (m), 1275 (m), 1243 (m), 1178 (w), 1024 (s), 976 (vs.), 894 (w), 798 (w), 755 (m), 741 (m), 683 (m), 660 cm^−1^ (m). Elemental analysis calcd (%) for C_59_H_51_N_2_BF_20_Mg (*M*=1203.15 g mol^−1^): C, 58.90; H, 4.27; N, 2.33; found: C, 59.41; H, 4.35; N, 2.04.

**Synthesis of [(^Me^BDI)Mg^+^(eb)][B(C_6_F_5_)_4_** ^**−**^ **] (7)**: [(^Me^BDI)Mg*n*Bu]_2_ (0.0815 g, 0.0817 mmol) and [Ph_3_C^+^][B(C_6_F_5_)_4_^−^] (0.121 g, 0.131 mmol) were dissolved in chlorobenzene (0.3 mL) and stirred until the solution became almost colorless (1 min). After filtration, a mixture of eb (0.1 mL) and methylcyclohexane (0.3 mL) was added and the reaction mixture left at −20 °C for 3 days. Crystallization was initiated by scratching the glass wall of the vial with a spatula. The crystalline product was washed with *n*‐hexane (5×4 mL), briefly dried under vacuum and isolated in a yield of 40 % (0.0635 g, 0.0527 mmol). ^1^H NMR (400 MHz, C_6_D_5_Br_,_ 298 K): *δ*=7.20–7.14 (m, 3 H, Ar*H*), 7.06–7.04 (m, 3 H, Ar*H*), 4.95 (s, 1 H, CC*H*C), 4.74–4.71 (m, 2 H, C*H_2_* (eb)), 2.76 (hept, ^3^ *J*_HH_=6.7 Hz, 4 H, C*H*Me_2_), 1.94 (q, ^3^ *J*_HH_=7.4 Hz, 4 H, C*H_2_* (eb)), 1.58 (s, 6 H, CC*H_3_*), 1.06 (d, ^3^ *J*_HH_=6.7 Hz, 12 H, CH(C*H*_3_)_2_), 0.95 (t, ^3^ *J*_HH_=7.4 Hz, 6 H, C*H_3_* (eb)), 0.89 (d, ^3^ *J*_HH_=6.7 Hz, 12 H, CH(C*H*_3_)_2_) ppm. ^13^C{^1^H} NMR (101 MHz, C_6_D_5_Br, 298 K): *δ*=173.4 (s, N*C*(CH_3_)), 153.1 (s, *C* (eb)), 149.1 (br d, ^1^ *J*_CF_=244 Hz, B(*C_6_*F_5_)_4_), 142.0 (s, Ar*C*), 141.5 (s, Ar*C*), 138.7 (br d, ^1^ *J*_CF_=244 Hz, B(*C_6_*F_5_)_4_), 137.0 (br d, ^1^ *J*_CF_=244 Hz, B(*C_6_*F_5_)_4_), 128.8 (s, Ar*C*), 127.3 (s, Ar*C*H), 126.7 (s, Ar*C*H), 124.8 (s, Ar*C*H), 106.8 (*C*H_2_ (eb)), 96.9 (s, C*C*HC), 29.3 (*C*H_2_ (eb)), 28.8 (s, *C*HMe_2_), 24.3 (s, CH*C*H_3_), 24.2 (s, NC(*C*H_3_)), 24.1 (s, CH*C*H_3_), 12.8 ppm (s, *C*H_3_ (eb)). Quaternary signal for eb can only be assigned by HMBC, B‐*C* of B(*C_6_*F_5_)_4_ was not detected. ^19^F{^1^H} NMR (376 MHz, C_6_D_5_Br, 298 K): *δ*=−131.6 (s, 8 F, *o*‐C*F*), −159.4 (t, ^3^ *J*_FF_=21 Hz, 4 F, *p*‐C*F*), −164.7 ppm (s, 8 F, *m*‐C*F*). ^11^B{^1^H} NMR (128 MHz, C_6_D_5_Br, 298 K) *δ*=−15.6 ppm (s, *B*(C_6_F_5_)_4_). IR (ATR, neat): $\tilde{\nu }\;$ =2965 (m), 2930 (w), 2871 (w), 1643 (m), 1512 (m), 1460 (vs.), 1372 (s), 1310 (m), 1261 (m), 1243 (m), 1177 (w), 1085 (s), 976 (vs.), 902 (w), 798 (w), 773 (m), 702 (m), 661 (m), 610 cm^−1^ (m). Elemental analysis calcd (%) for C_59_H_53_N_2_BF_20_Mg (*M*=1205.17 g mol^−1^): C, 58.80; H, 4.43; N, 2.32; found: C, 58.27; H, 4.47; N, 2.29.

## Conflict of interest

The authors declare no conflict of interest.

## Supporting information

As a service to our authors and readers, this journal provides supporting information supplied by the authors. Such materials are peer reviewed and may be re‐organized for online delivery, but are not copy‐edited or typeset. Technical support issues arising from supporting information (other than missing files) should be addressed to the authors.

Supplementary
