# Supplementary material for: Unsupported Mg–Alkene Bonding
Source: Chemistry. 2020 Dec 23;27(7):2513–22. doi: 10.1002/chem.202004716 (PMC7898539; doi:10.1002/chem.202004716)
Supplement: Supplementary file 1 — Supplementary [file CHEM-27-2513-s001.pdf]

Chemistry–A European Journal

Supporting Information

Unsupported Mg–Alkene Bonding

Katharina Thum, Alexander Friedrich, Jürgen Pahl, Holger Elsen, Jens Langer, and Sjoerd Harder*[a]

Contents

1. Supporting Experimental Data S1

1.1 NMR Spectra S2

1.2 Single Crystal X-Ray Diffraction S25

2. Computational Details S36

3. References S49

1. Supporting Experimental Data

1.1 NMR Spectra

1.1.1 Spectra of free alkenes

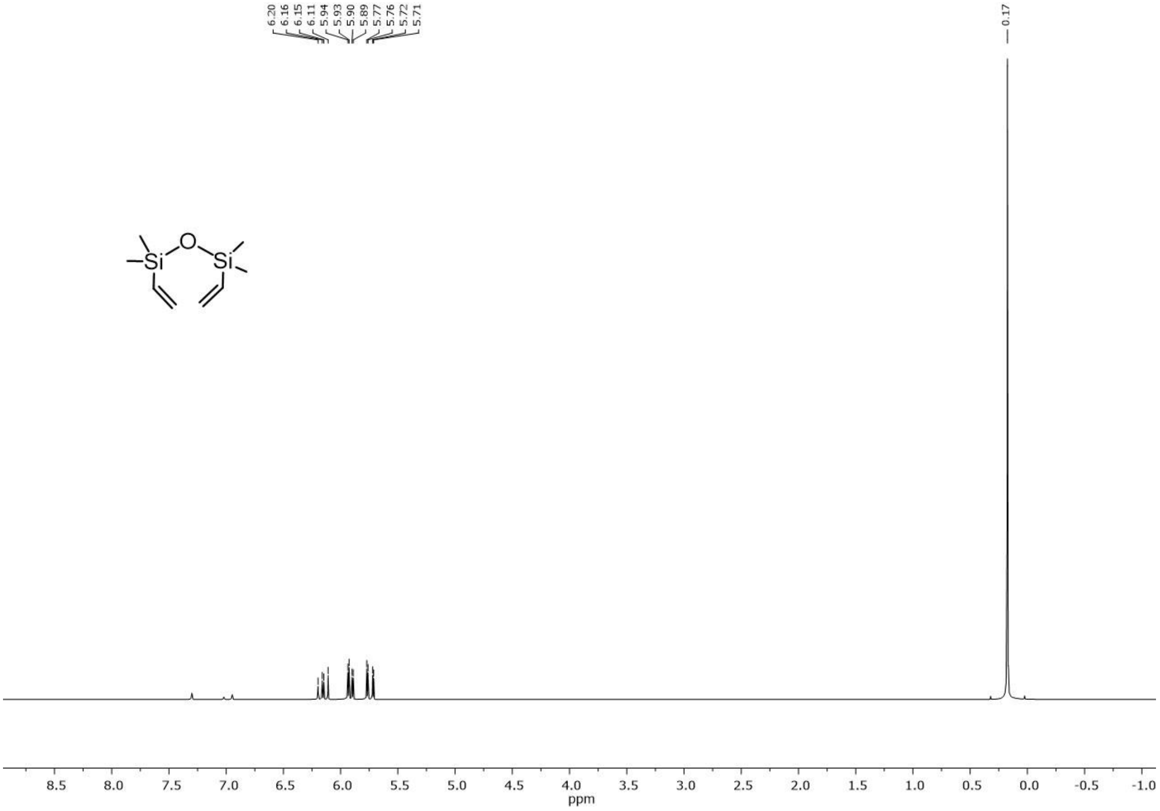

Figure S1: ^{1}H NMR (400 MHz, 298 K) spectrum of free *divinylsiloxane* in C_6D_5Br.

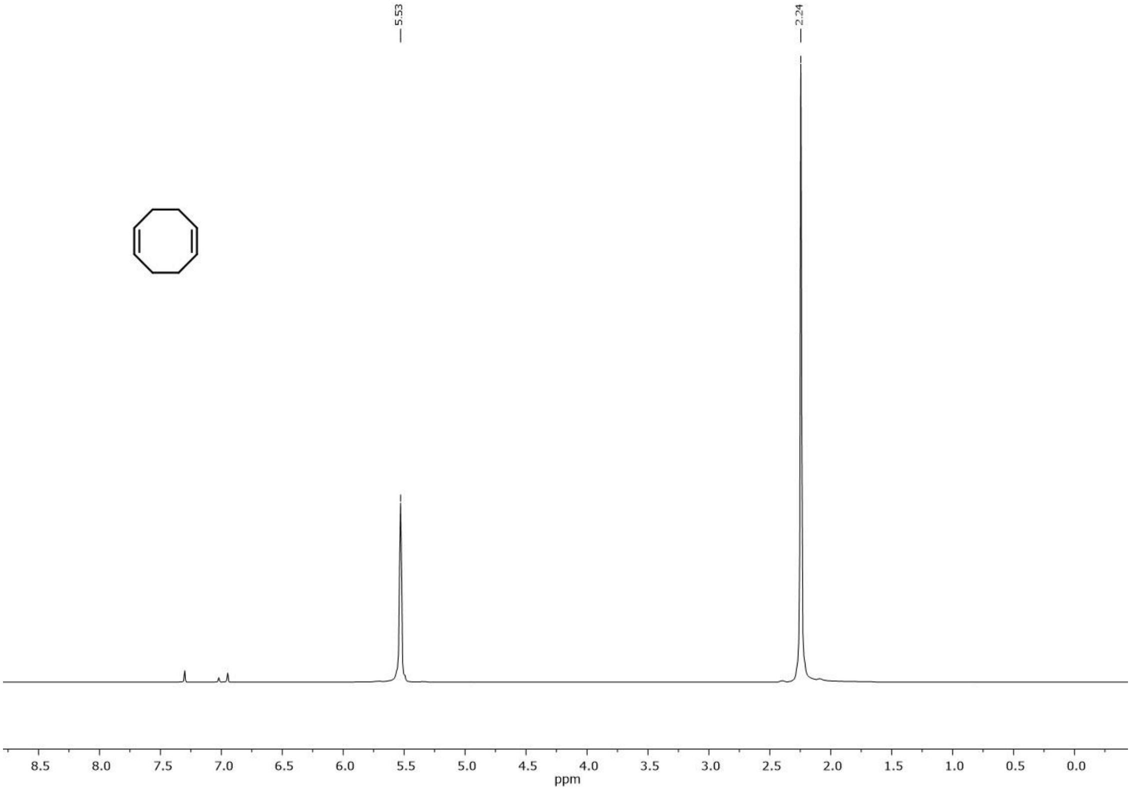

Figure S2: ^{1}H NMR spectrum (400 MHz, 298 K) of free *cod* in C_6D_5Br.

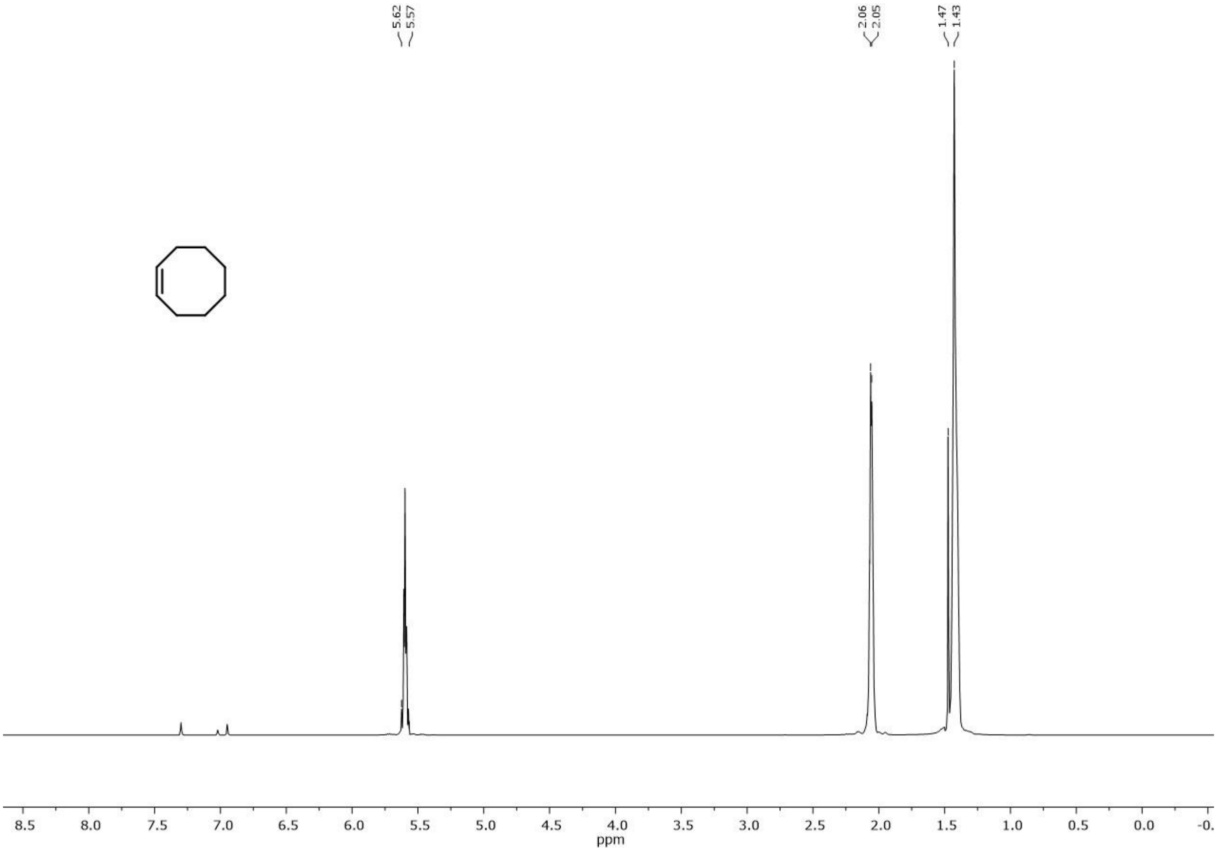

Figure S3: ^{1}H NMR spectrum (600 MHz, 298 K) of free *coe* in C_6D_5Br.

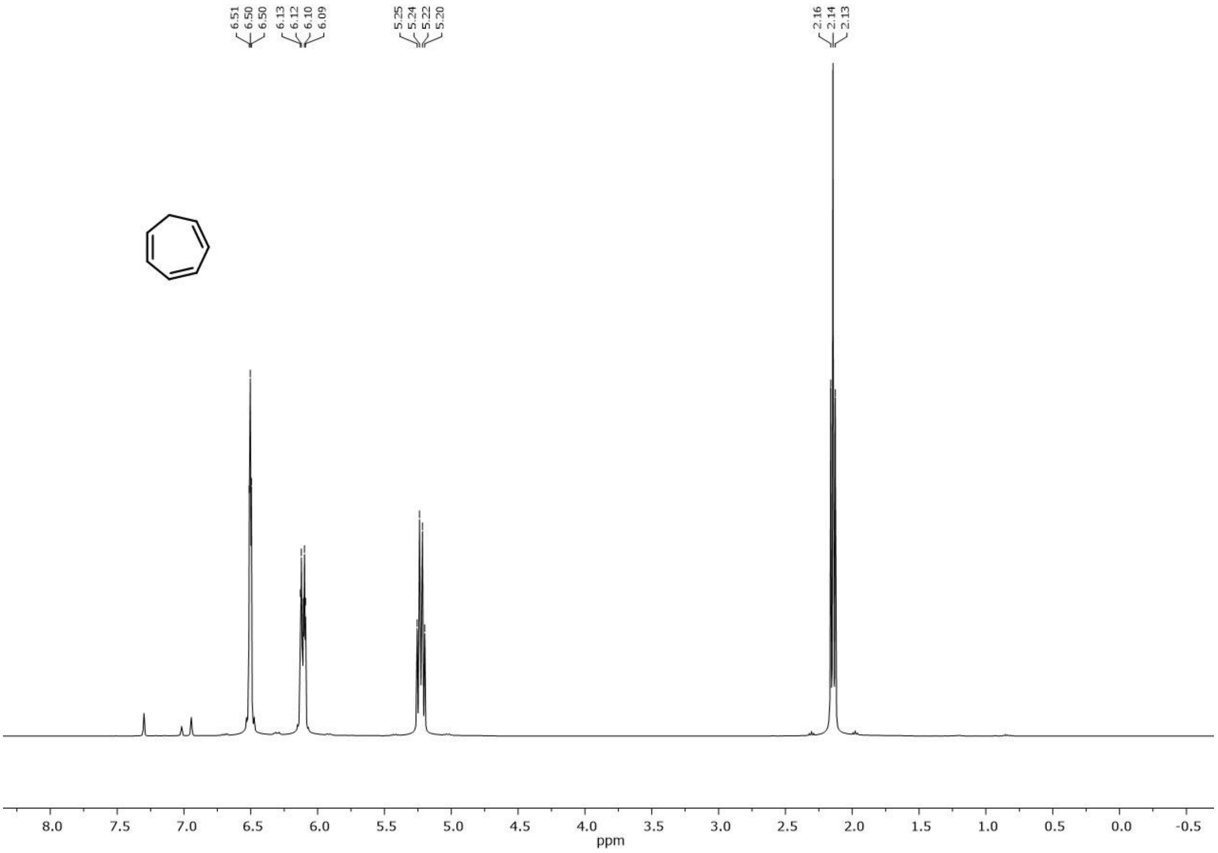

Figure S4: ^{1}H NMR spectrum (400 MHz, 298 K) of free *cht* in C_6D_5Br.

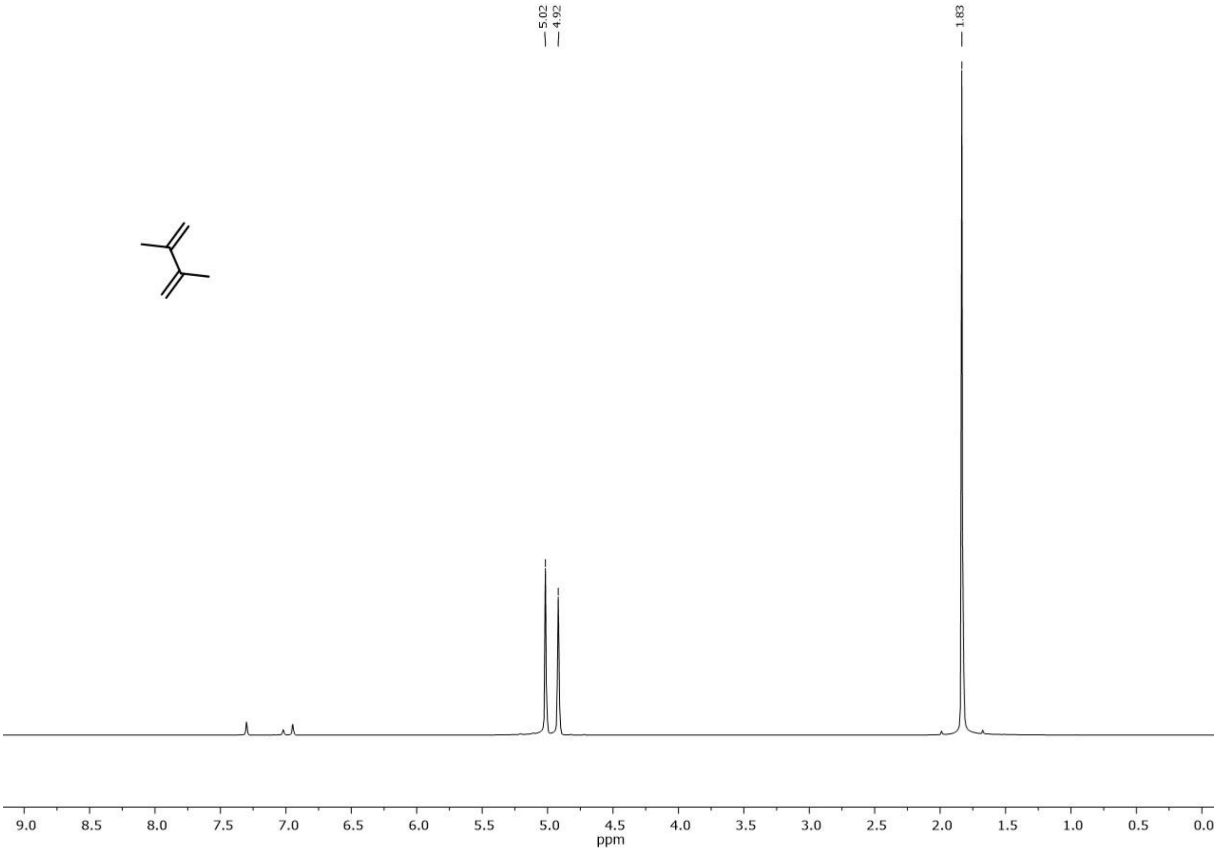

Figure S5: ^{1}H NMR spectrum (400 MHz, 298 K) of free *dmbd* in C_6D_5Br.

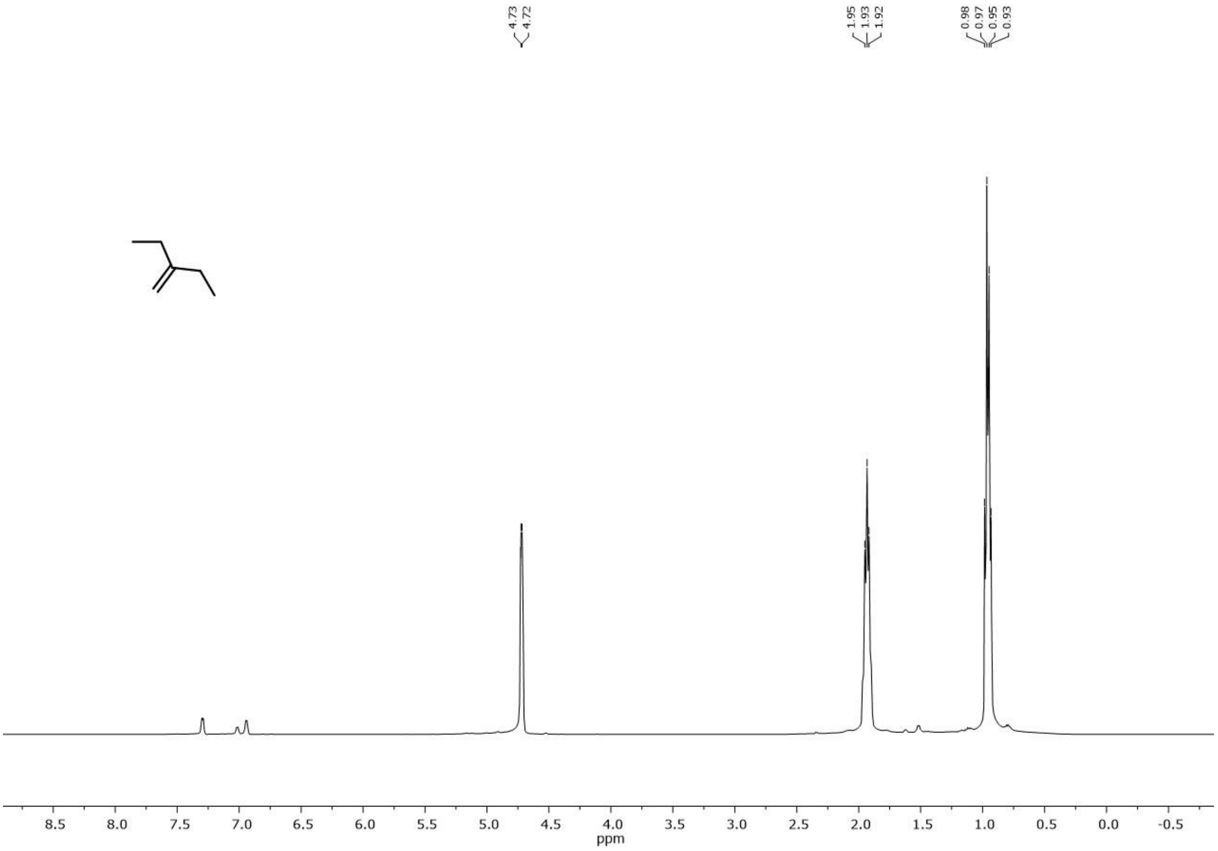

Figure S6: ^{1}H NMR spectrum (400 MHz, 298 K) of free *eb* in C_6D_5Br.

1.1.2 Spectra of $[(^{tBu}BDI)Mg^{+}(\textit{divinylsiloxane})][B(C_6F_5)_4^{-}]$ (1)

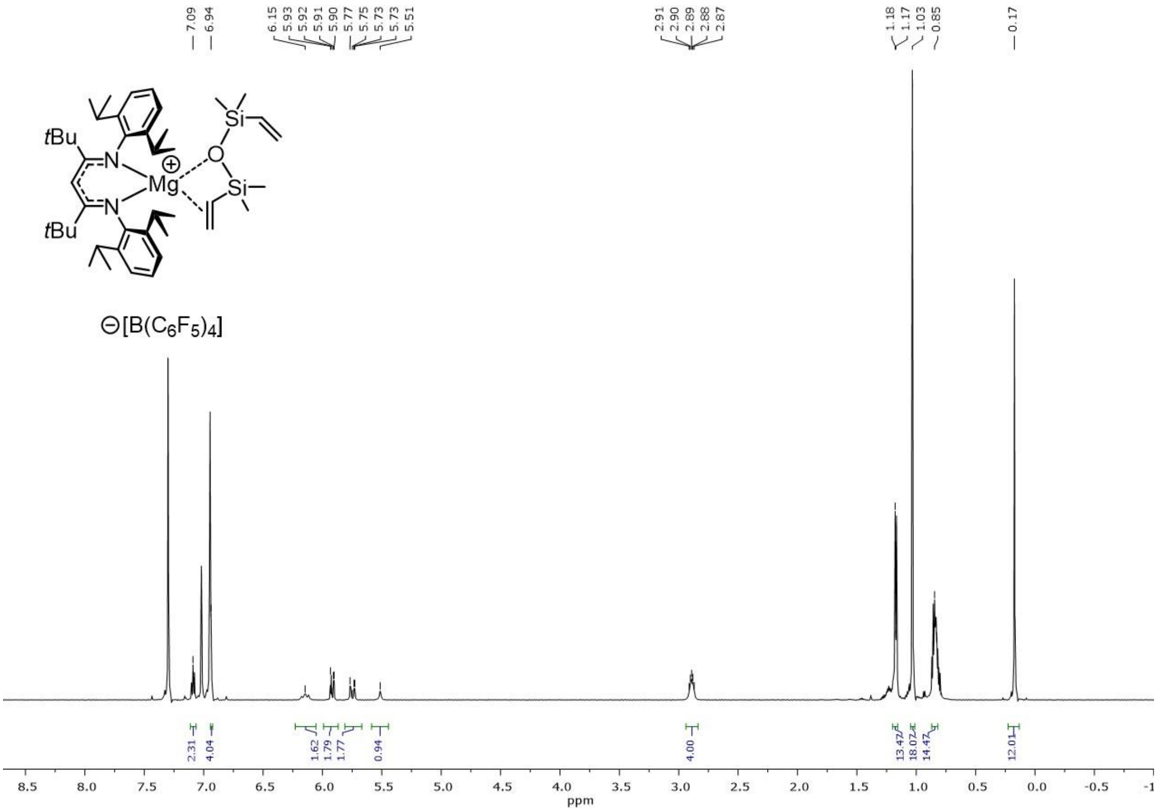

Figure S7: ^{1}H NMR spectrum (600 MHz, 298 K) of $[(^{tBu}BDI)Mg^{+}(\textit{divinylsiloxane})][B(C_6F_5)_4^{-}]$ in C_6D_5Br. Alkene chemical shifts correspond to shifts for free alkene. Signals are sharp → no coordination in solution.

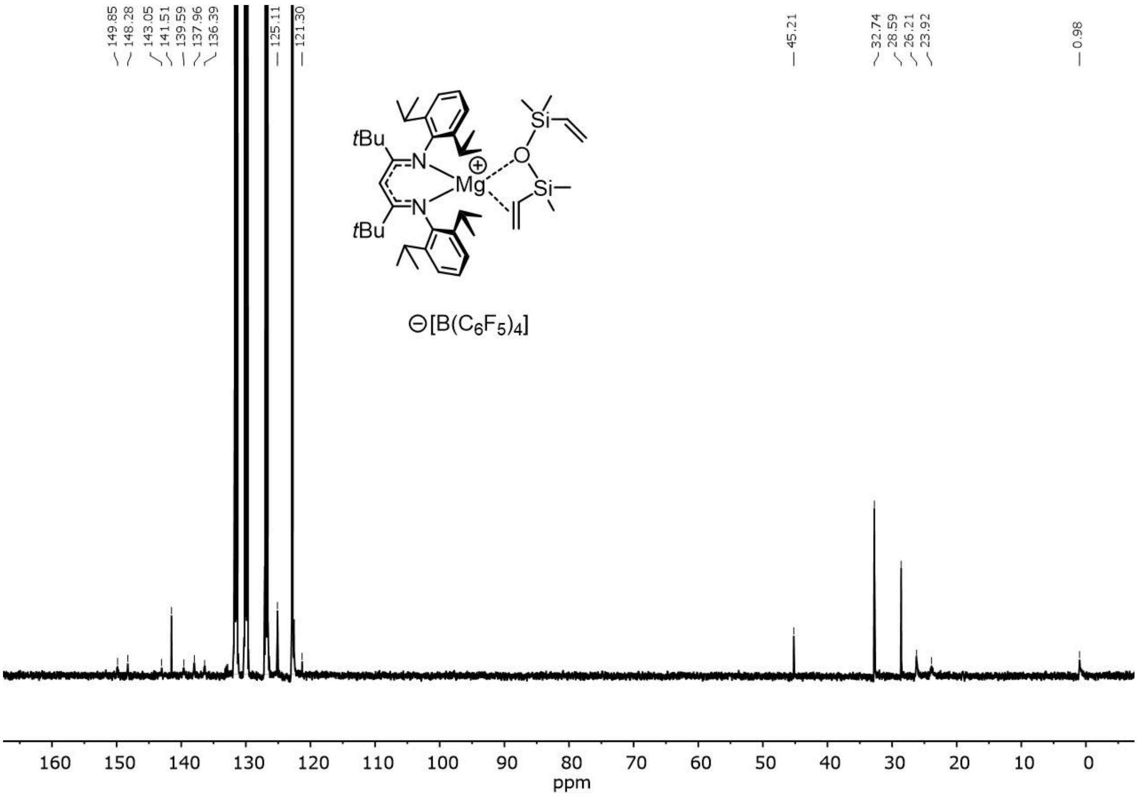

Figure S8: $^{13}C\{^{1}H\}$ NMR spectrum (151 MHz, 298 K) of $[(^{tBu}BDI)Mg^{+}(\textit{divinylsiloxane})][B(C_6F_5)_4^{-}]$ in C_6D_5Br.

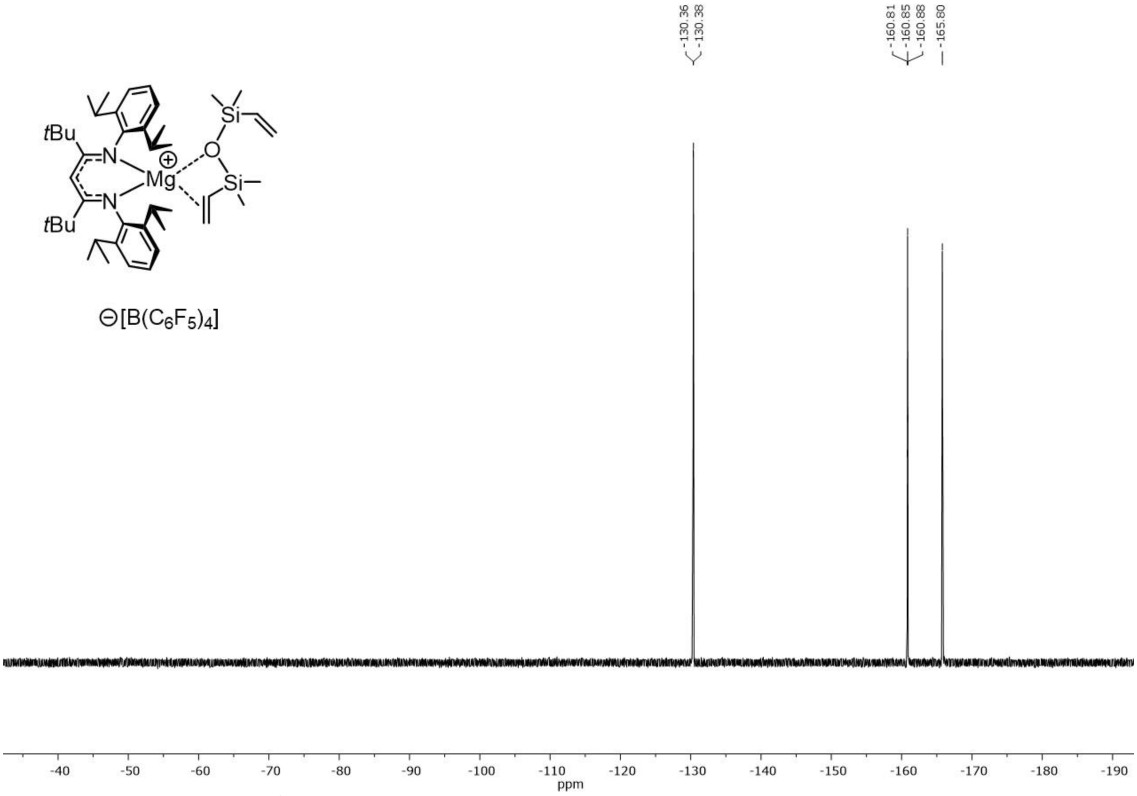

Figure S9: $^{19}F\{^{1}H\}$ NMR spectrum (565 MHz, 298 K) of $[(^{tBu}BDI)Mg^{+}$(*divinylsiloxane*)$][B(C_6F_5)_4^{-}]$ in C_6D_5Br.

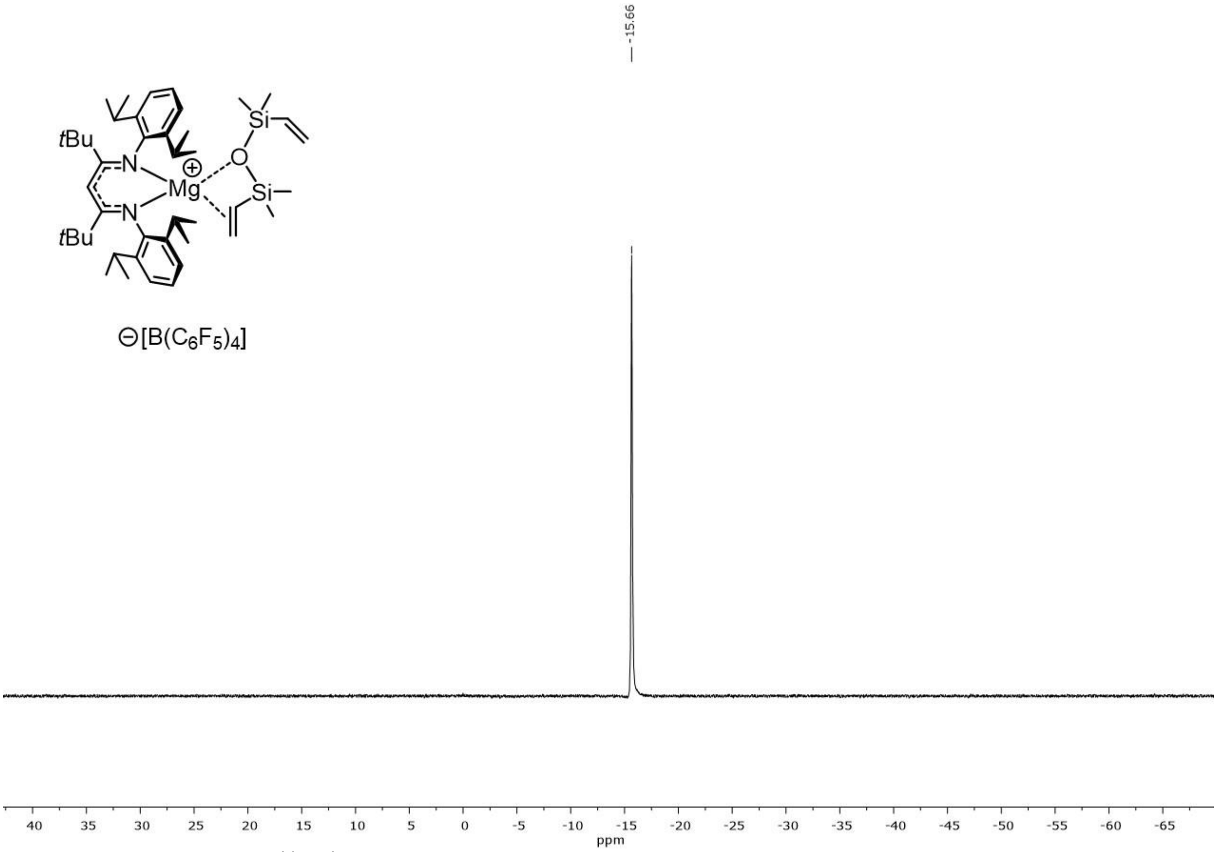

Figure S10: $^{11}B\{^{1}H\}$ NMR spectrum (193 MHz, 298 K) of $[(^{tBu}BDI)Mg^{+}$(*divinylsiloxane*)$][B(C_6F_5)_4^{-}]$ in C_6D_5Br.

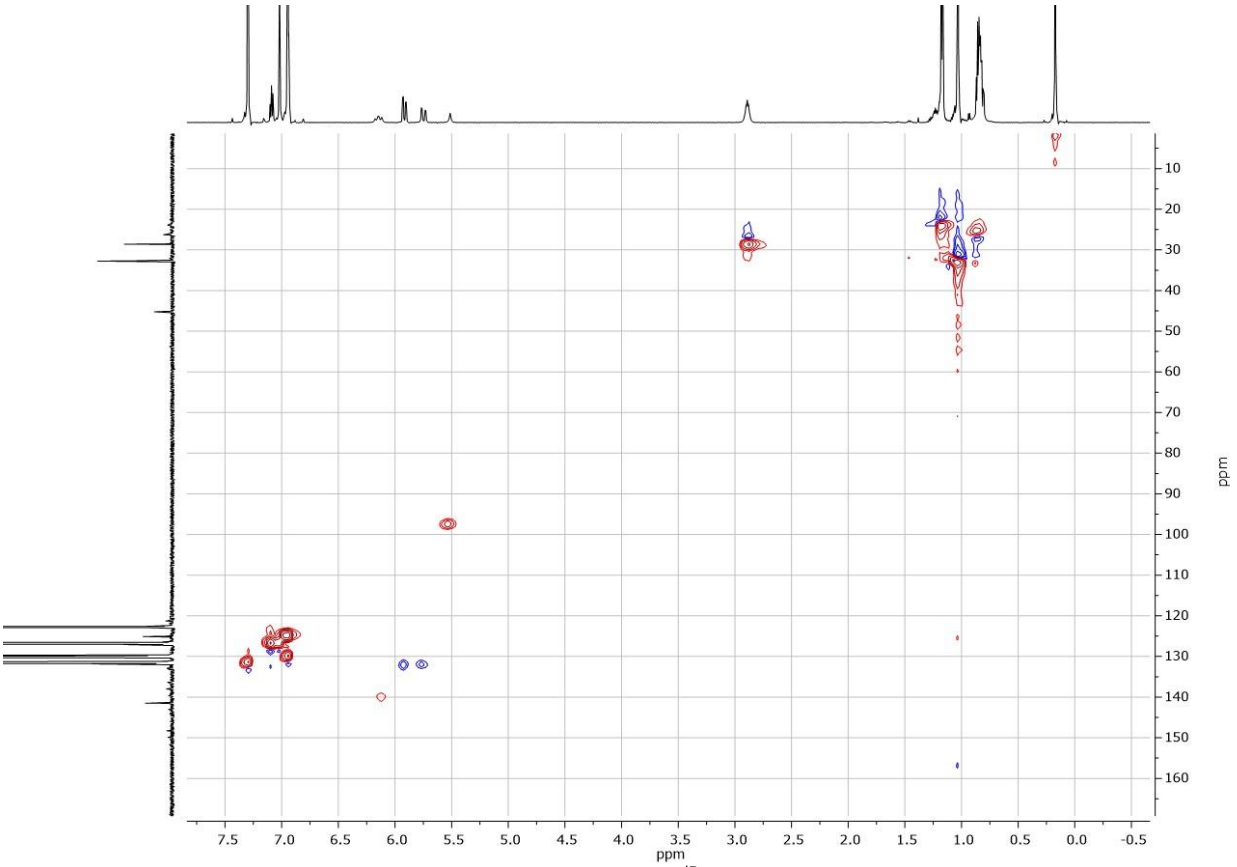

Figure S11: 2-dimensional HSQC spectrum of [(^{tBu}BDI)Mg^{+}(*divinylsiloxane*)][B(C_6F_5)$_4^{-}$] in C_6D_5Br.

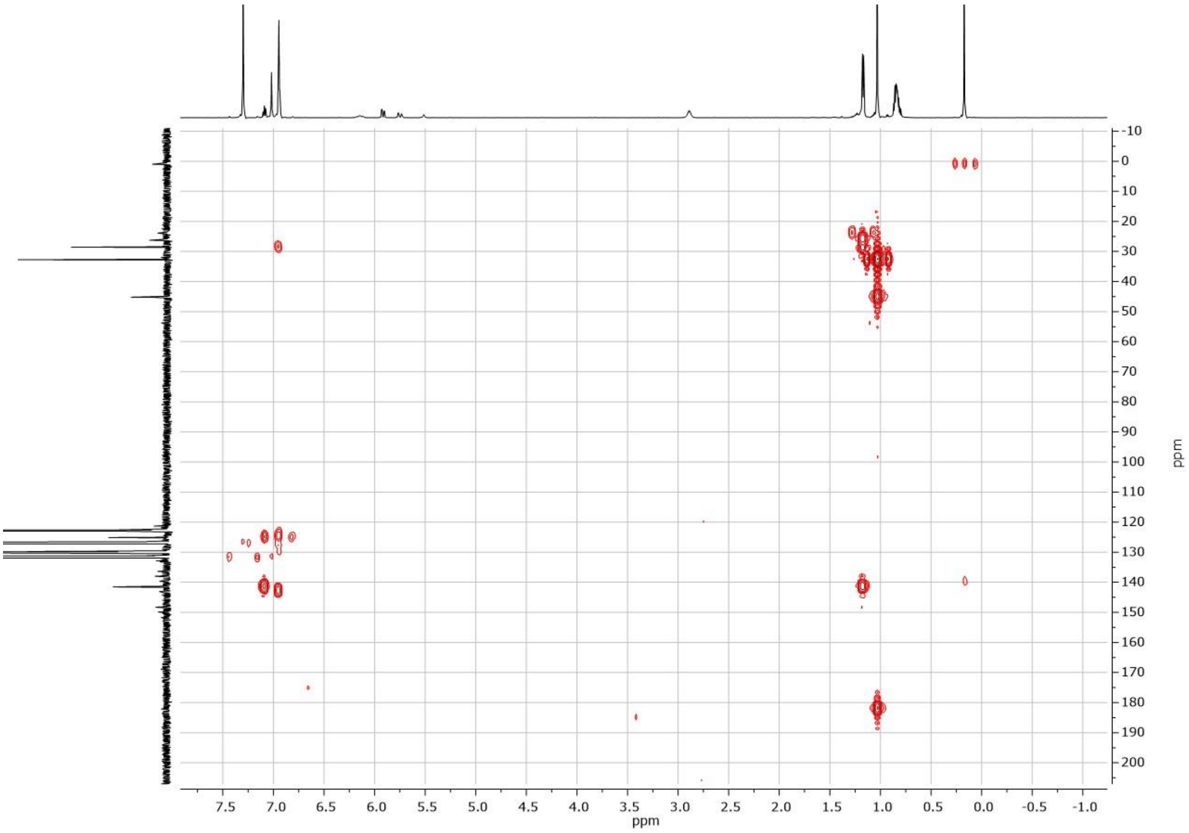

Figure S12: 2-dimensional HMBC spectrum of [(^{tBu}BDI)Mg^{+}(*divinylsiloxane*)][B(C_6F_5)$_4^{-}$] in C_6D_5Br.

1.1.3 Spectra of [(^{tBu}BDI)Mg$^+$(*cod*)][B(C_6F_5)$_4^-$] (2)

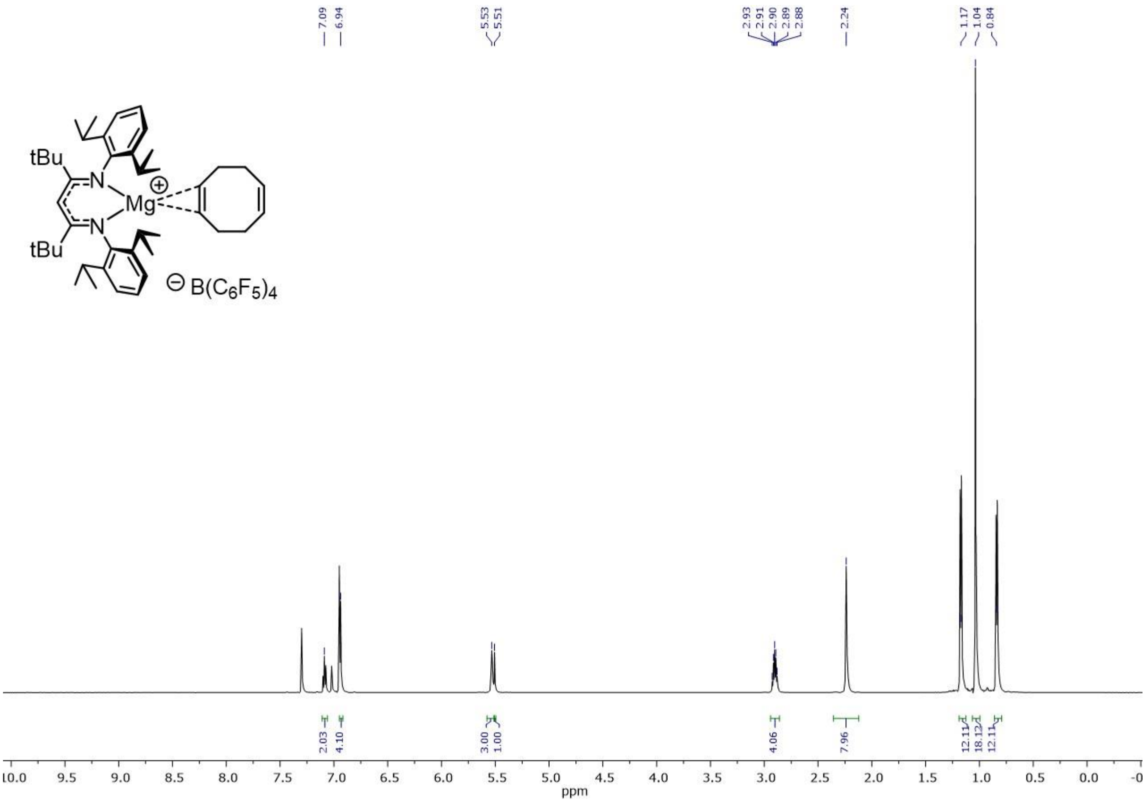

Figure S13: ^{1}H NMR spectrum (600 MHz, 298 K) of [(^{tBu}BDI)Mg$^+$(*cod*)][B(C_6F_5)$_4^-$] in C_6D_5Br. Alkene chemical shifts correspond to shifts for free alkene. Signals are sharp → no coordination in solution.

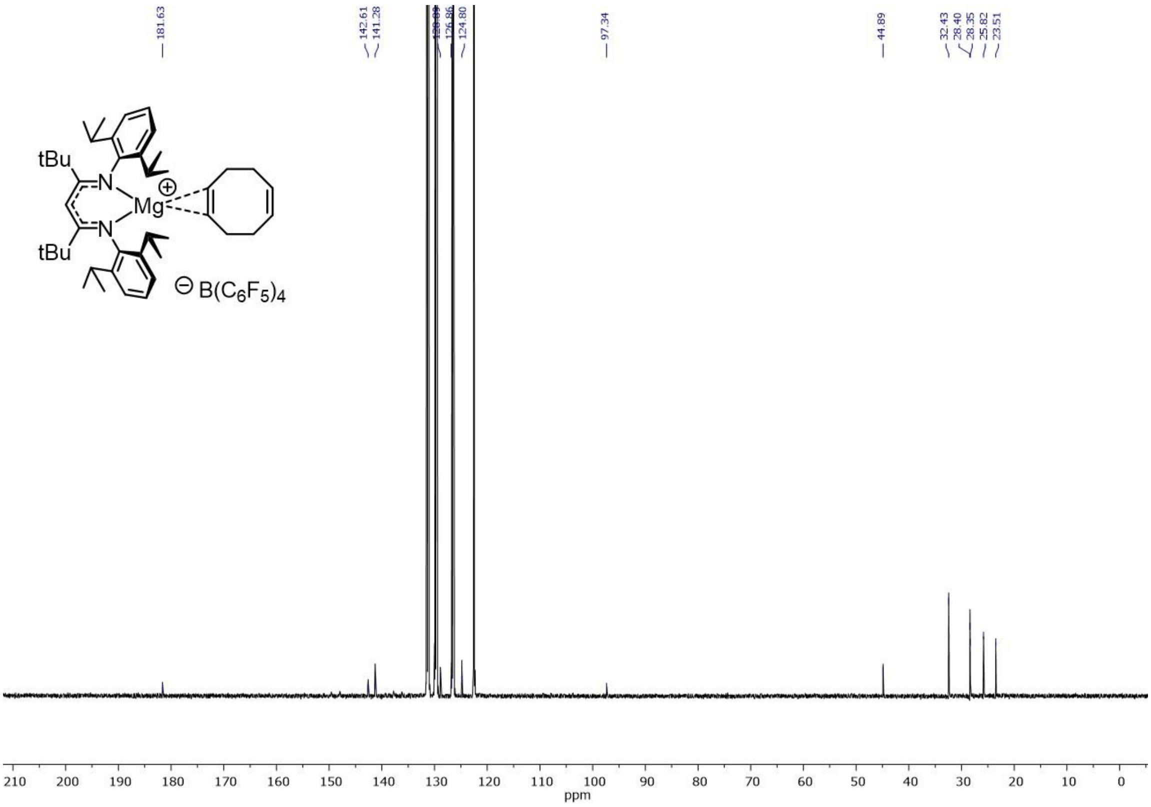

Figure S14: ^{13}C{^{1}H} NMR spectrum (151 MHz, 298 K) of [(^{tBu}BDI)Mg$^+$(*cod*)][B(C_6F_5)$_4^-$] in C_6D_5Br.

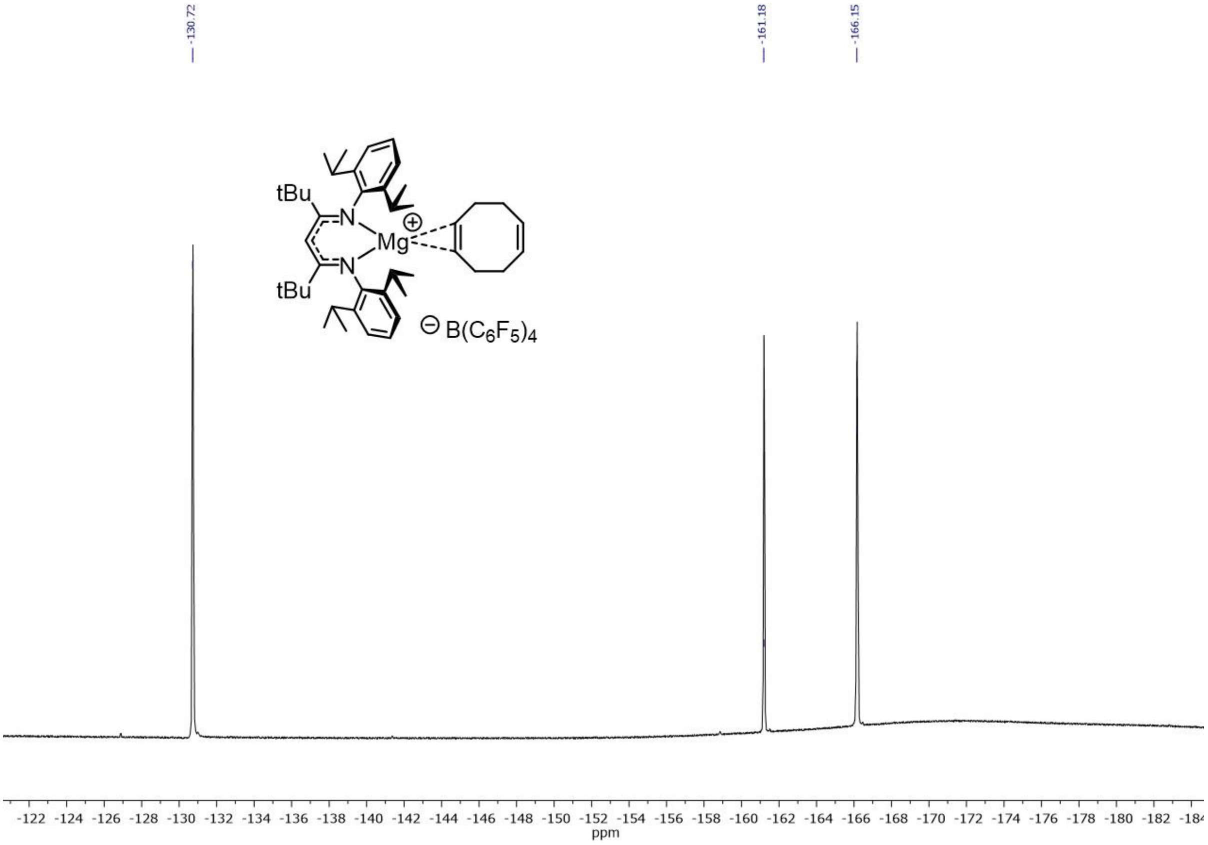

Figure S15: $^{19}F\{^{1}H\}$ NMR spectrum (565 MHz, 298 K) of $[(^{tBu}BDI)Mg^{+}(cod)][B(C_6F_5)_4^{-}]$ in C_6D_5Br.

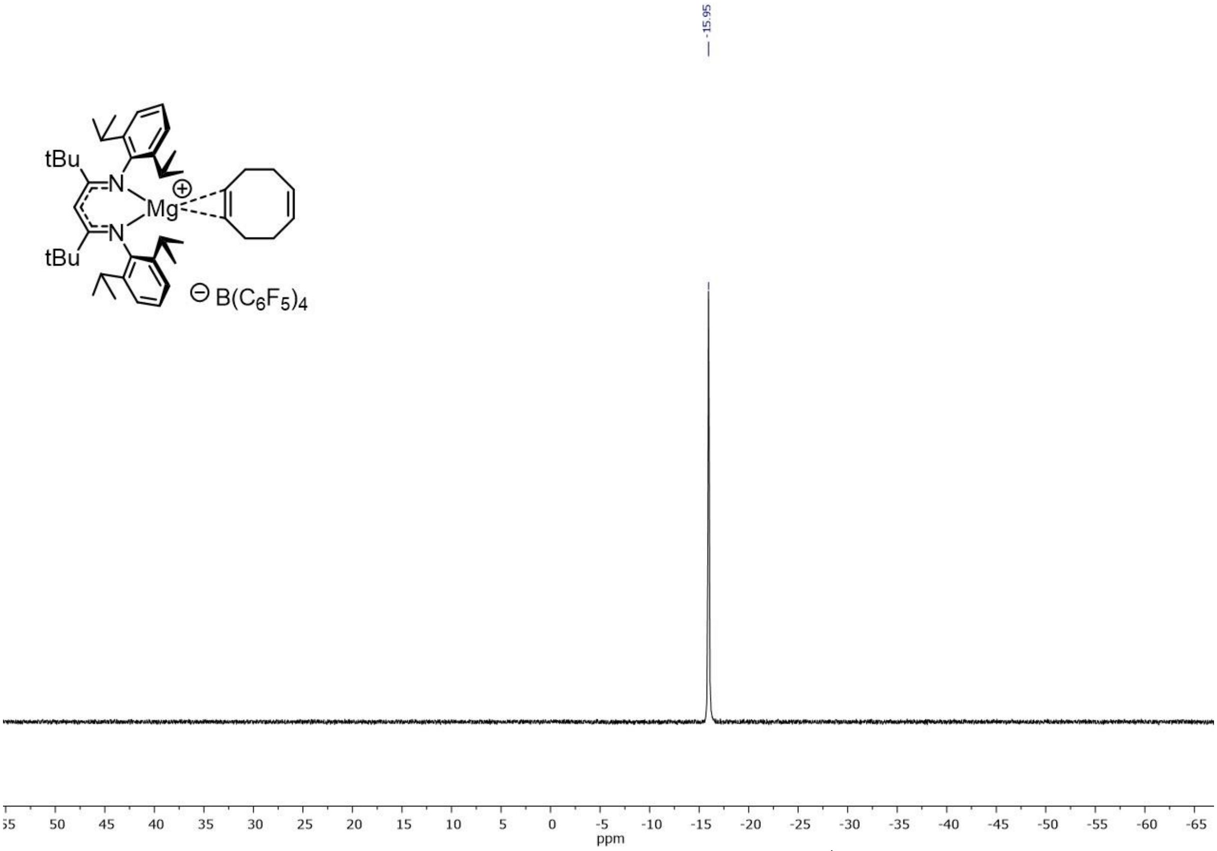

Figure S16: $^{11}B\{^{1}H\}$ NMR spectrum (193 MHz, 298 K) of $[(^{tBu}BDI)Mg^{+}(cod)][B(C_6F_5)_4^{-}]$ in C_6D_5Br.

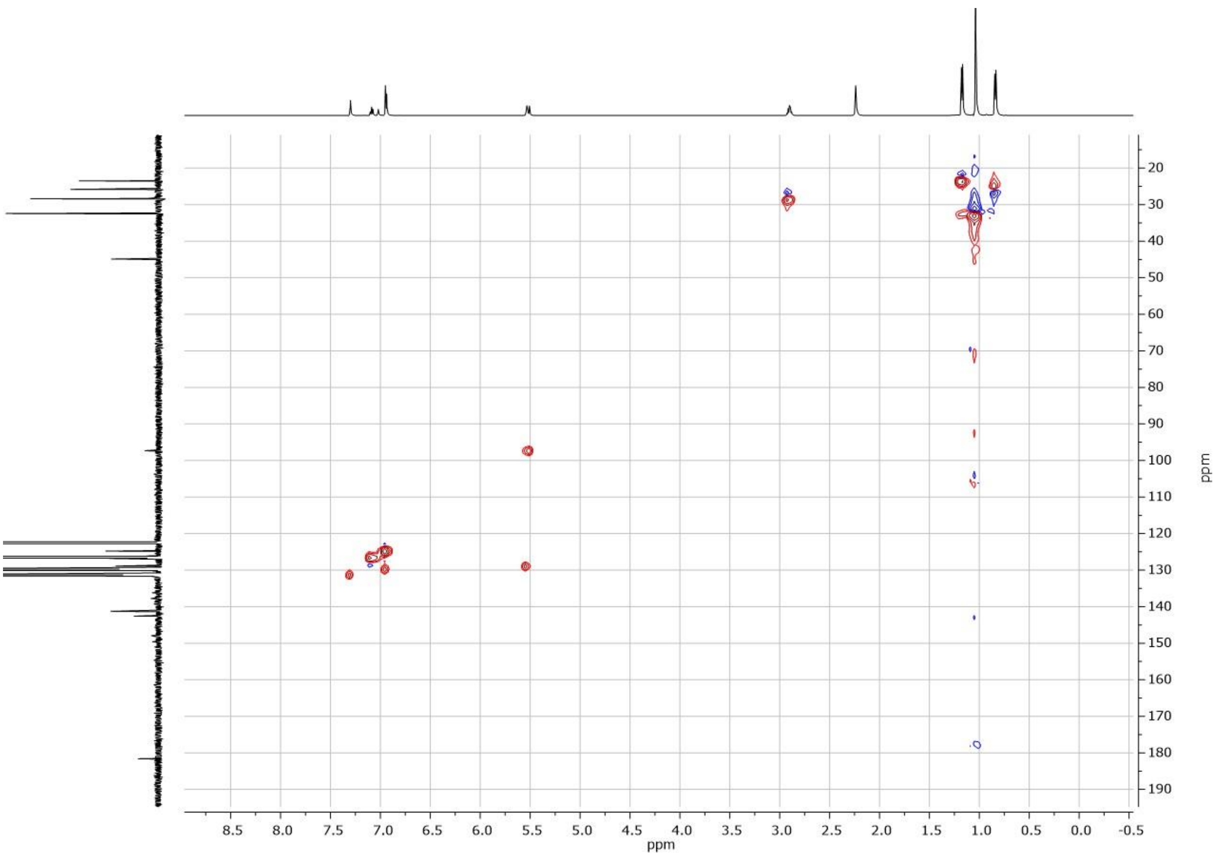

Figure S17: 2-dimensional HSQC spectrum of [(^{tBu}BDI)Mg$^+$(*cod*)][B(C_6F_5)$_4^-$] in C_6D_5Br.

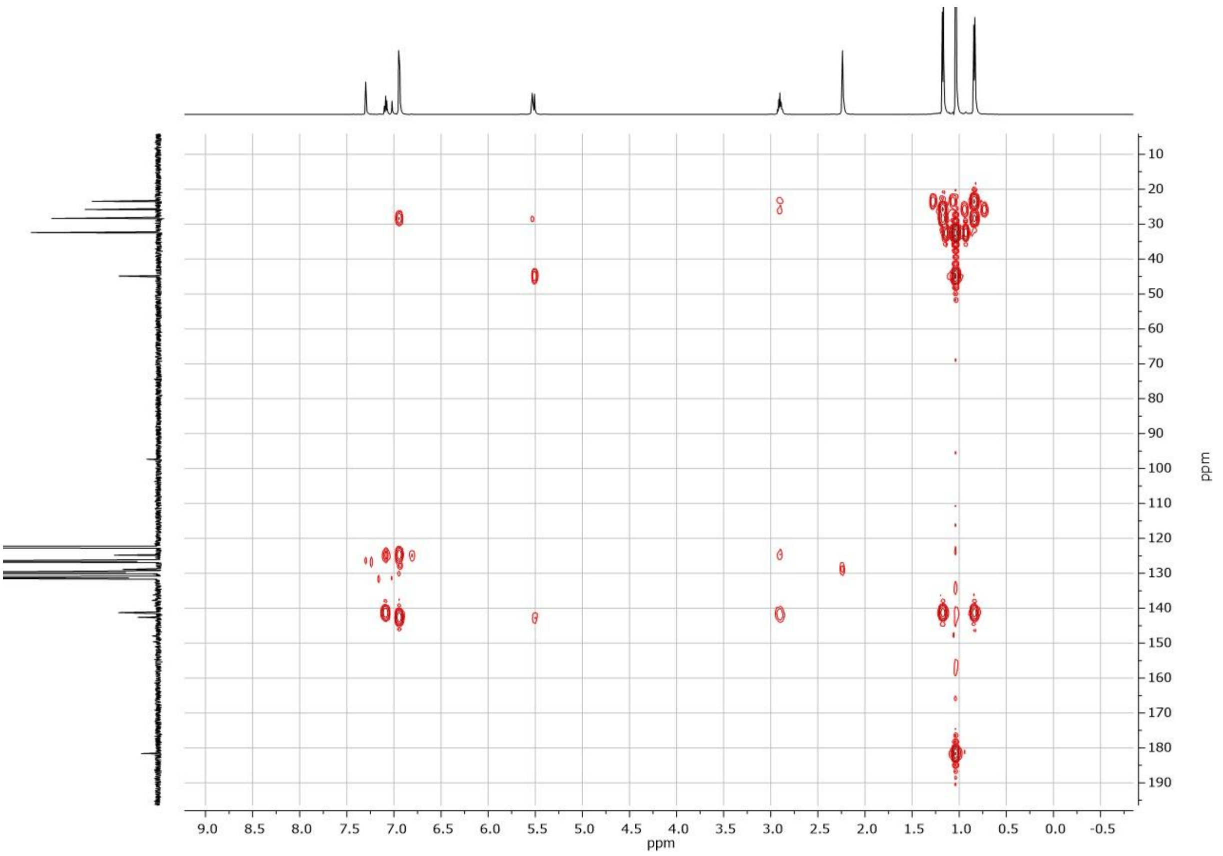

Figure S18: 2-dimensional HMBC spectrum of [(^{tBu}BDI)Mg$^+$(*cod*)][B(C_6F_5)$_4^-$] in C_6D_5Br.

1.1.4 Spectra of [(^{tBu}BDI)Mg$^+$(*coe*)][B(C_6F_5)$_4^-$] (3)

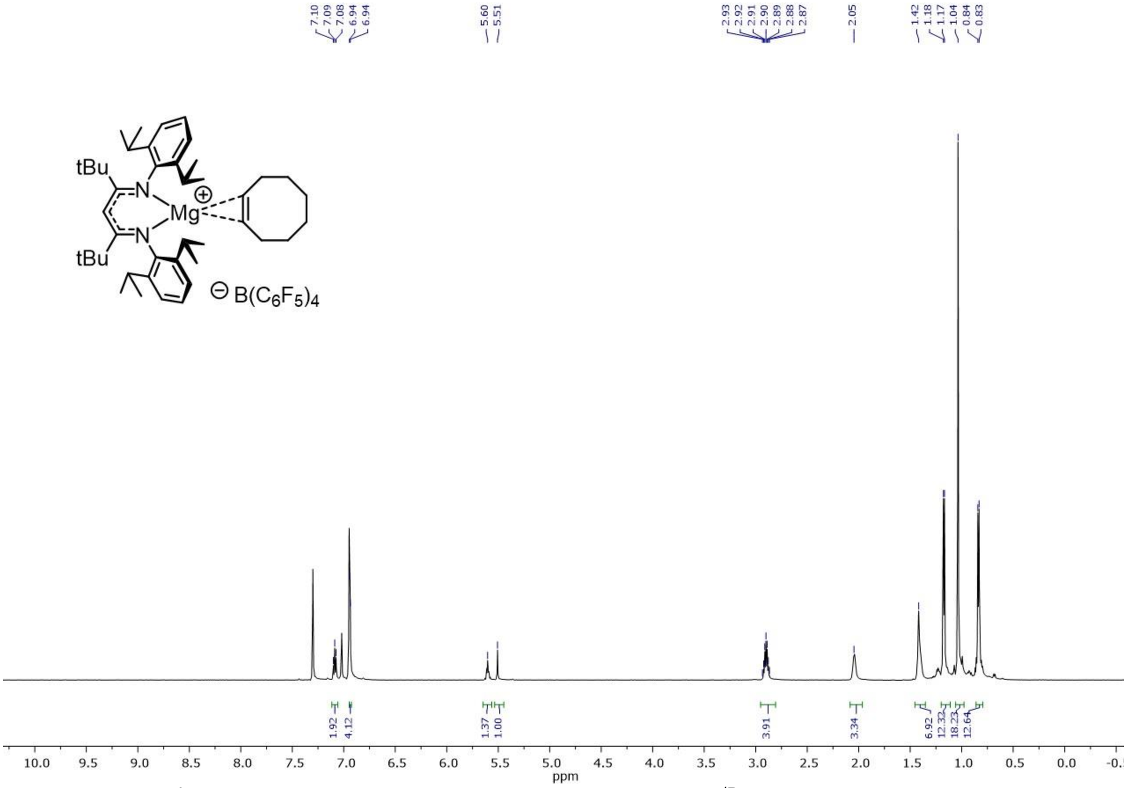

Figure S19: ^{1}H NMR spectrum (600 MHz, 298 K) of [(^{tBu}BDI)Mg$^+$(*coe*)][B(C_6F_5)$_4^-$] in C_6D_5Br. Alkene chemical shifts correspond to shifts for free alkene. Signals are sharp → no coordination in solution.

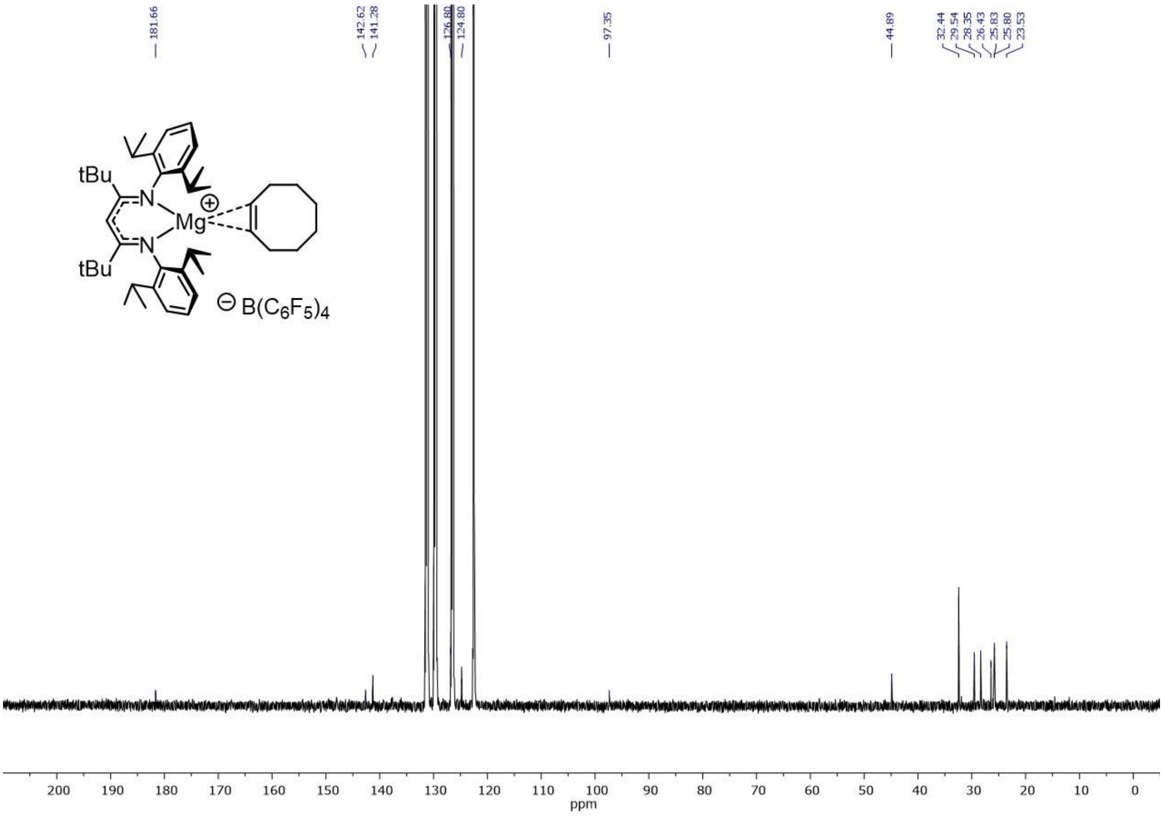

Figure S20: ^{13}C{^{1}H} NMR spectrum (151 MHz, 298 K) of [(^{tBu}BDI)Mg$^+$(*coe*)][B(C_6F_5)$_4^-$] in C_6D_5Br.

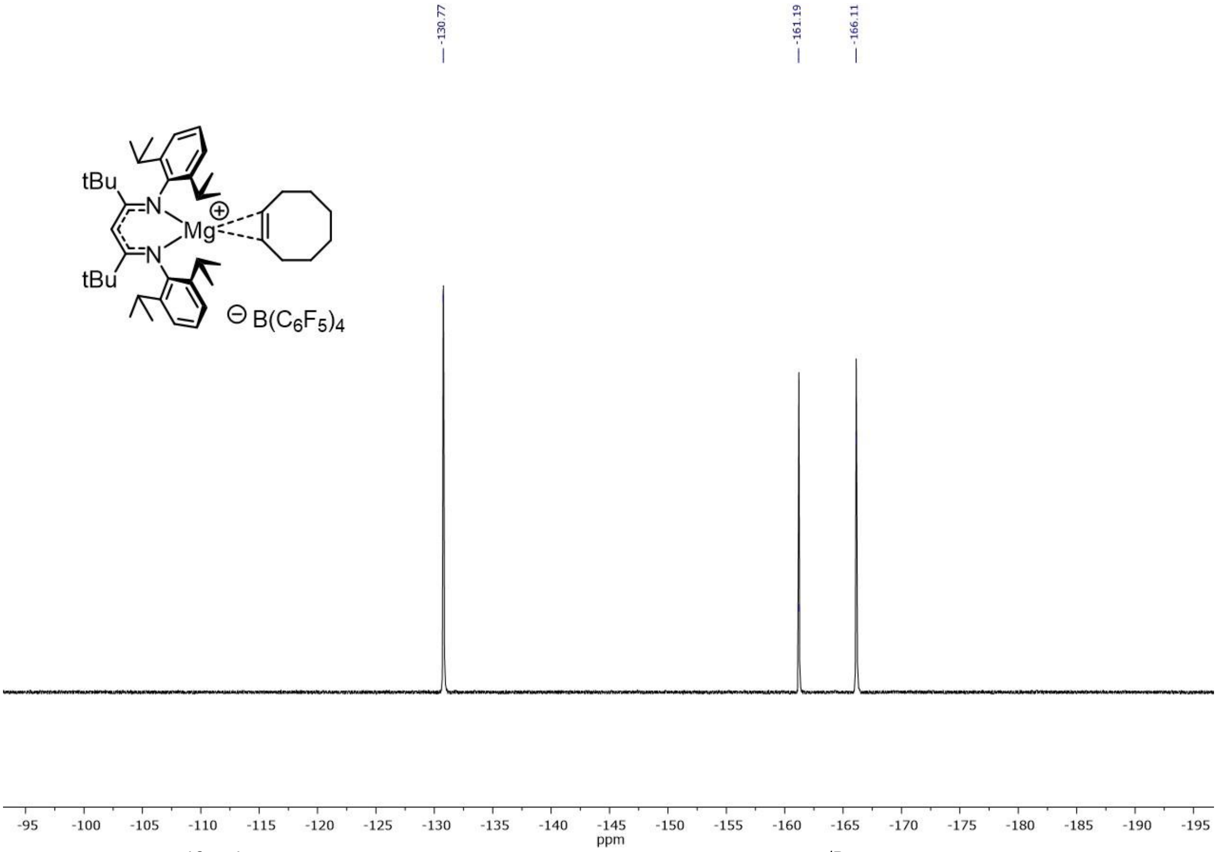

Figure S21: $^{19}F\{^1H\}$ NMR spectrum (565 MHz, 298 K) of $[(^{tBu}BDI)Mg^+(coe)][B(C_6F_5)_4^-]$ in C_6D_5Br.

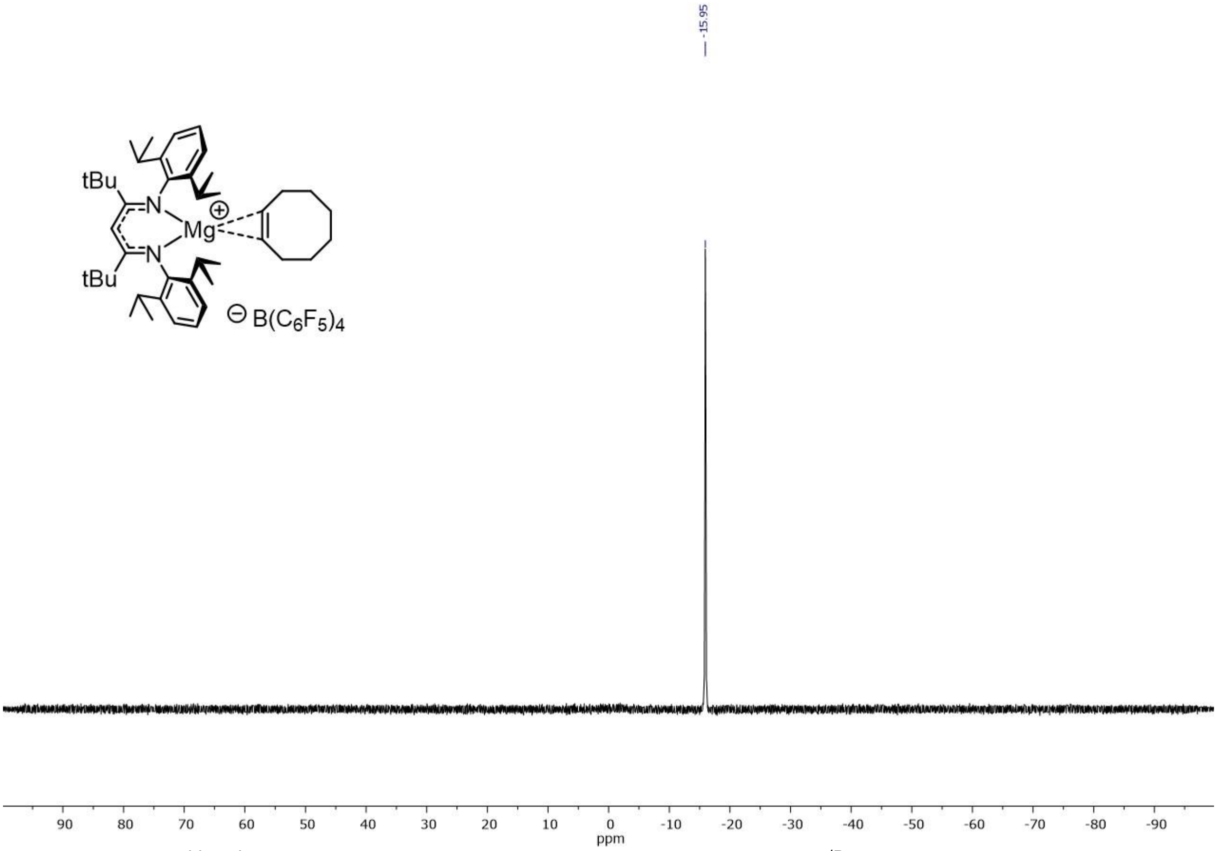

Figure S22: $^{11}B\{^1H\}$ NMR spectrum (193 MHz, 298 K) of $[(^{tBu}BDI)Mg^+(coe)][B(C_6F_5)_4^-]$ in C_6D_5Br.

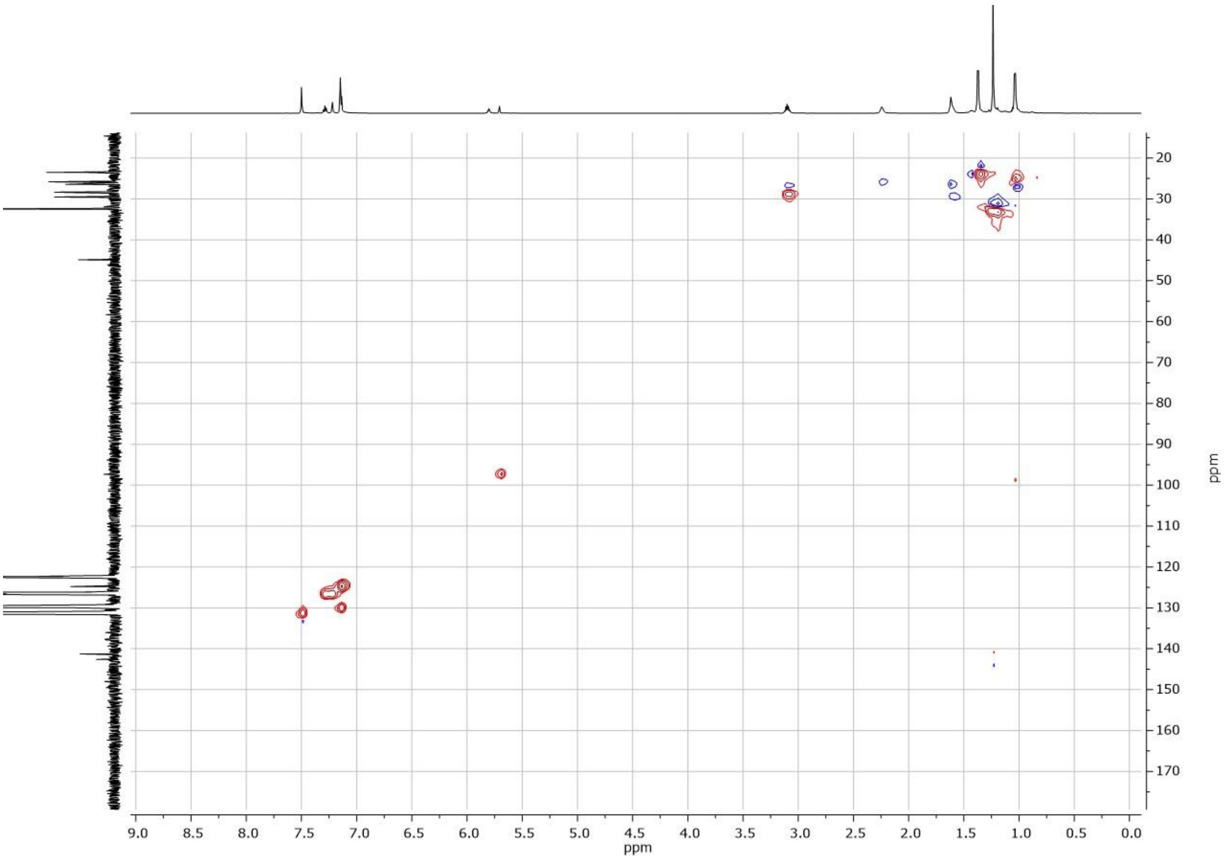

Figure S23: 2-dimensional HSQC spectra of [(^{tBu}BDI)Mg$^+$(*coe*)][B(C_6F_5)$_4^-$] in C_6D_5Br.

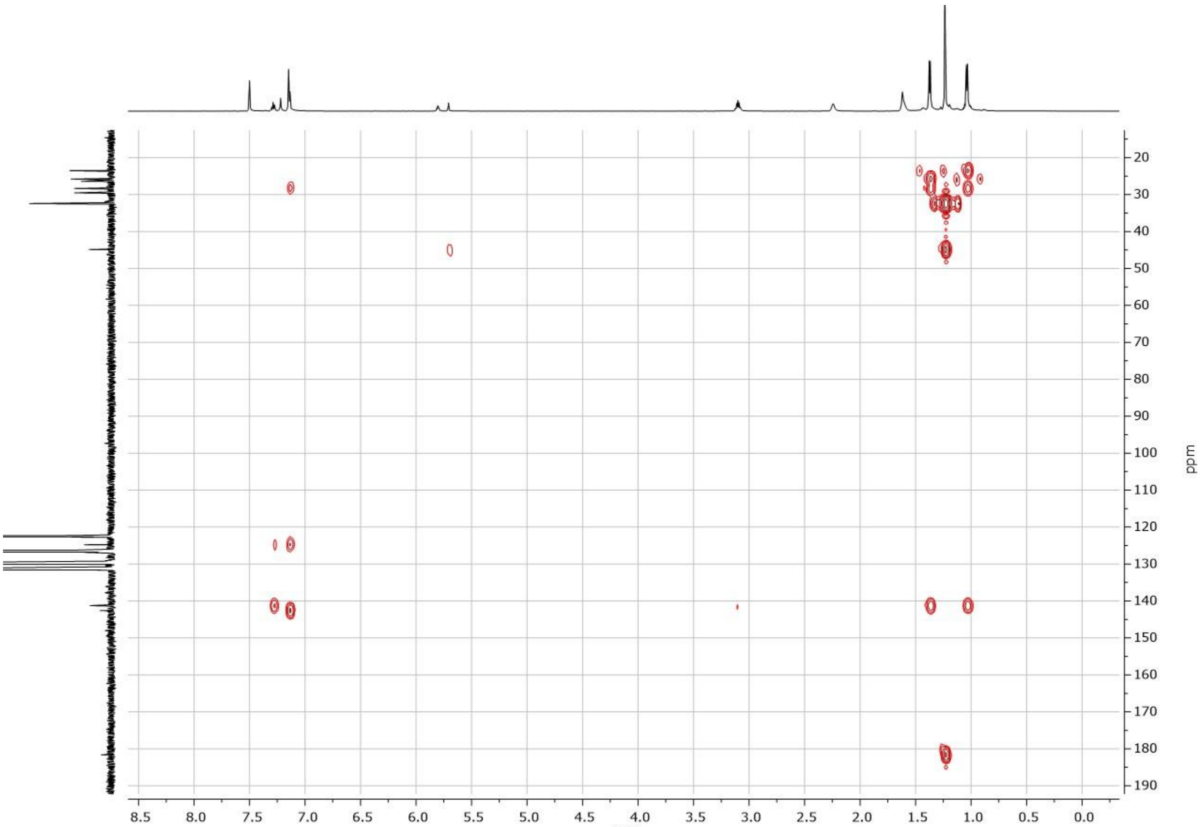

Figure S24: 2-dimensional HMBC spectra of [(^{tBu}BDI)Mg$^+$(*coe*)][B(C_6F_5)$_4^-$] in C_6D_5Br.

1.1.5 Spectra of [(^{Me}BDI)Mg^{+}(*cod*)][B(C_6F_5)$_4^{-}$] (4)

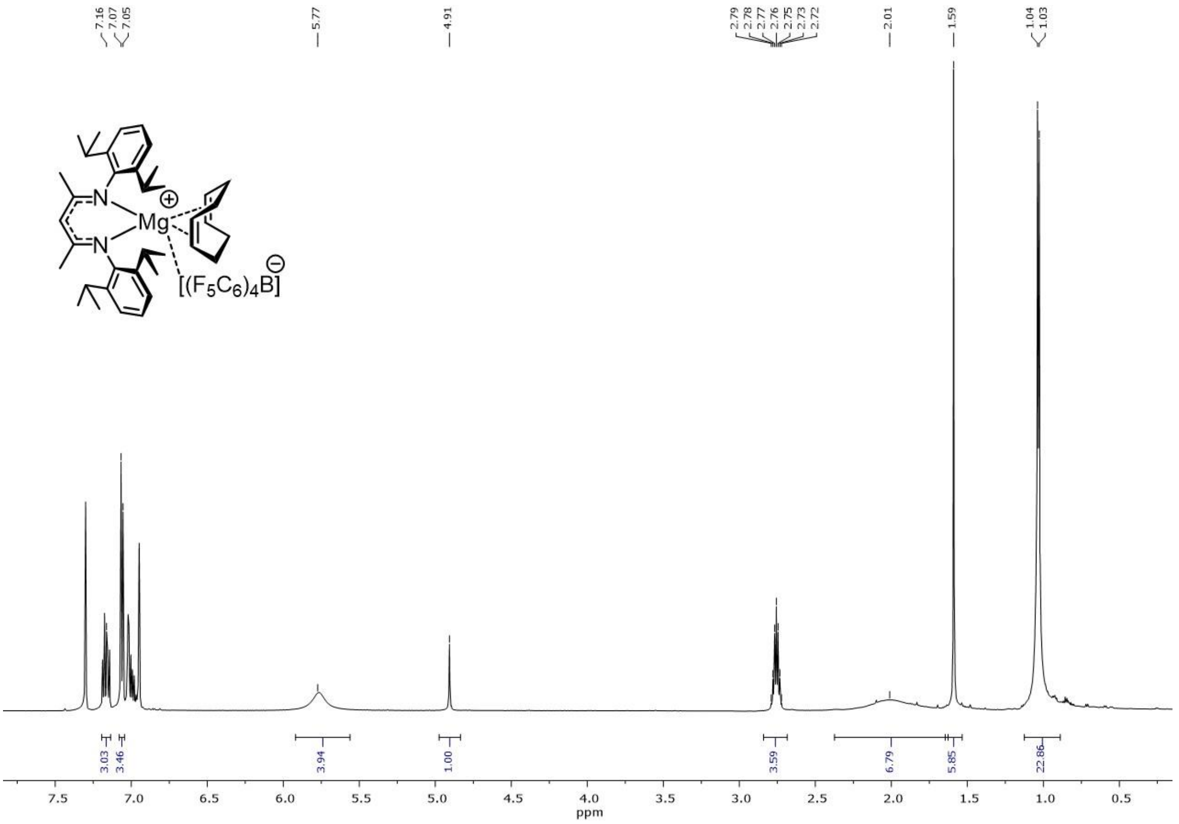

Figure S25: ^{1}H NMR spectrum (600 MHz, 298 K) of [(^{Me}BDI)Mg^{+}(*cod*)][B(C_6F_5)$_4^{-}$] in C_6D_5Br. Alkene chemical shifts do not correspond to shifts for free alkene. Signals are broadened → *cod* partially bound in solution.

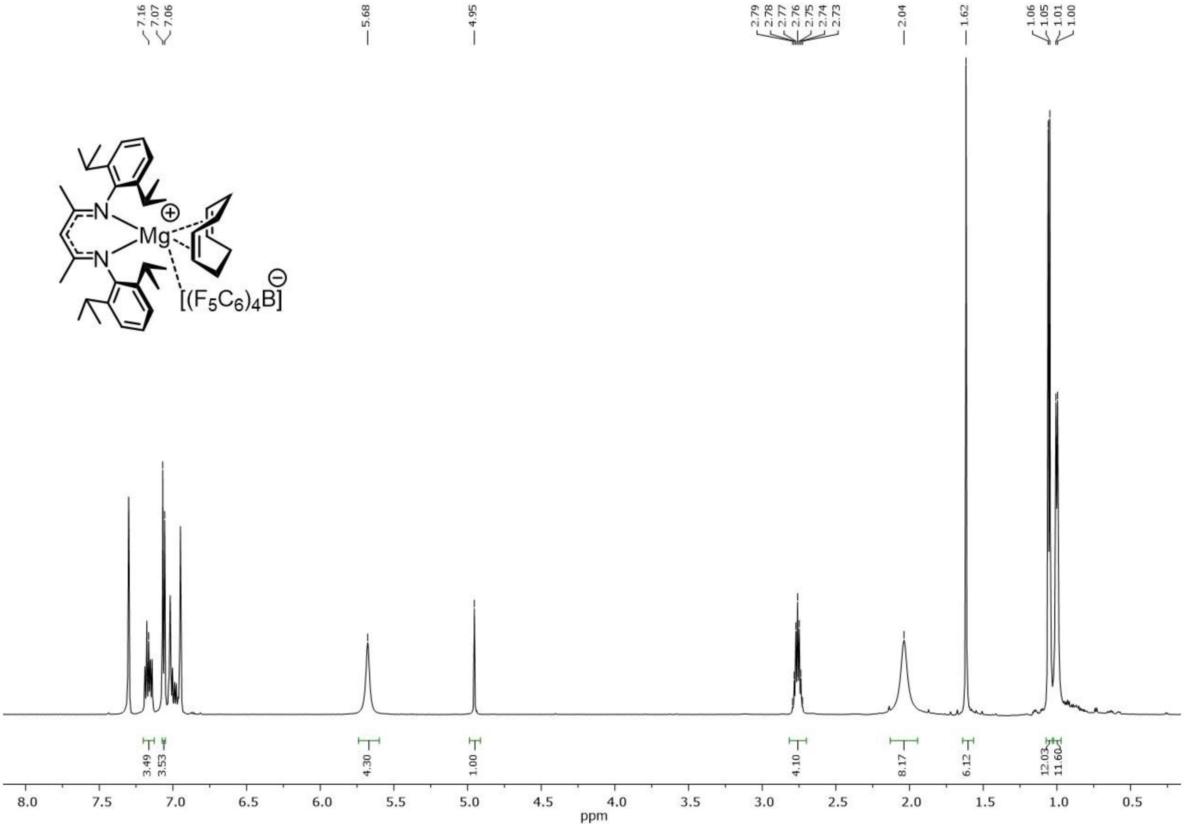

Figure S26: ^{1}H NMR spectrum (600 MHz, 328 K) of [(^{Me}BDI)Mg^{+}(*cod*)][B(C_6F_5)$_4^{-}$] in C_6D_5Br.

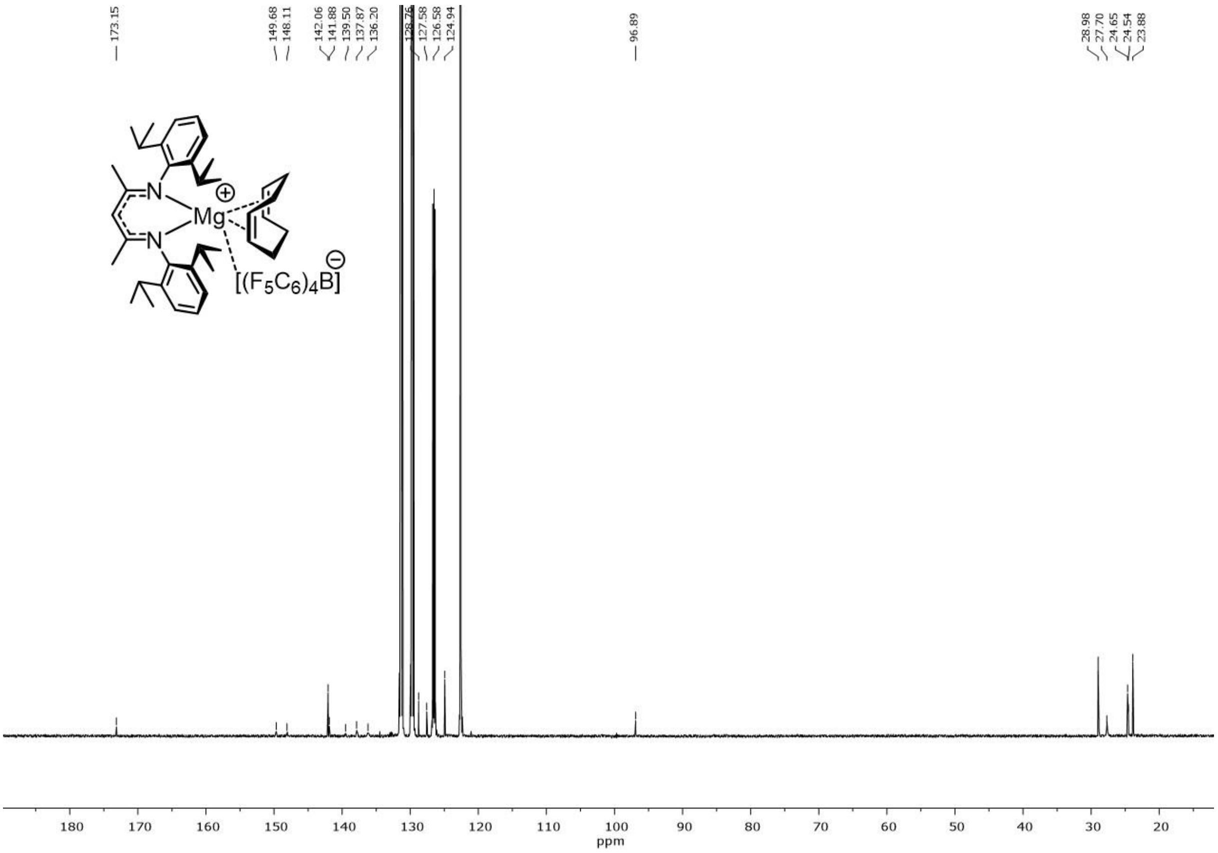

Figure S27: $^{13}C\{^1H\}$ NMR spectrum (151 MHz, 328 K) of $[(^{Me}BDI)Mg^+(cod)][B(C_6F_5)_4^-]$ in C_6D_5Br.

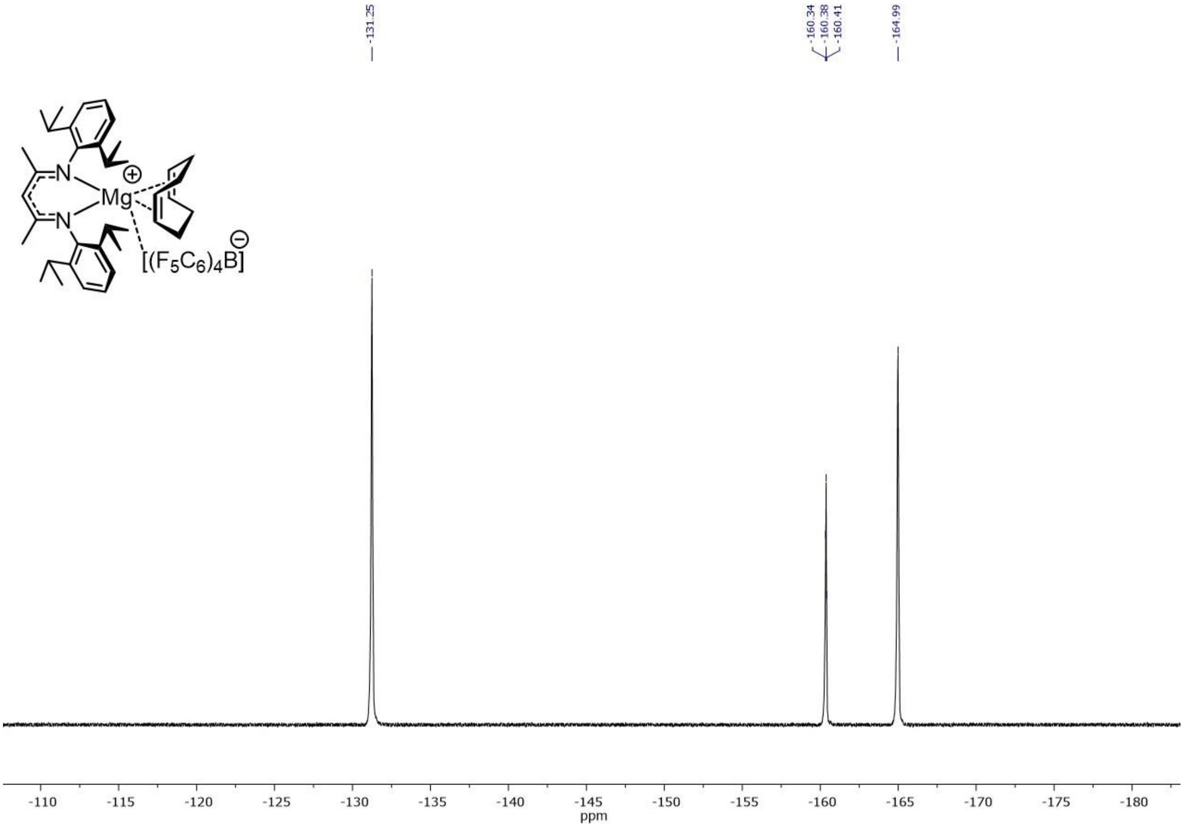

Figure S28: $^{19}F\{^1H\}$ NMR spectrum (565 MHz, 298 K) of $[(^{Me}BDI)Mg^+(cod)][B(C_6F_5)_4^-]$ in C_6D_5Br.

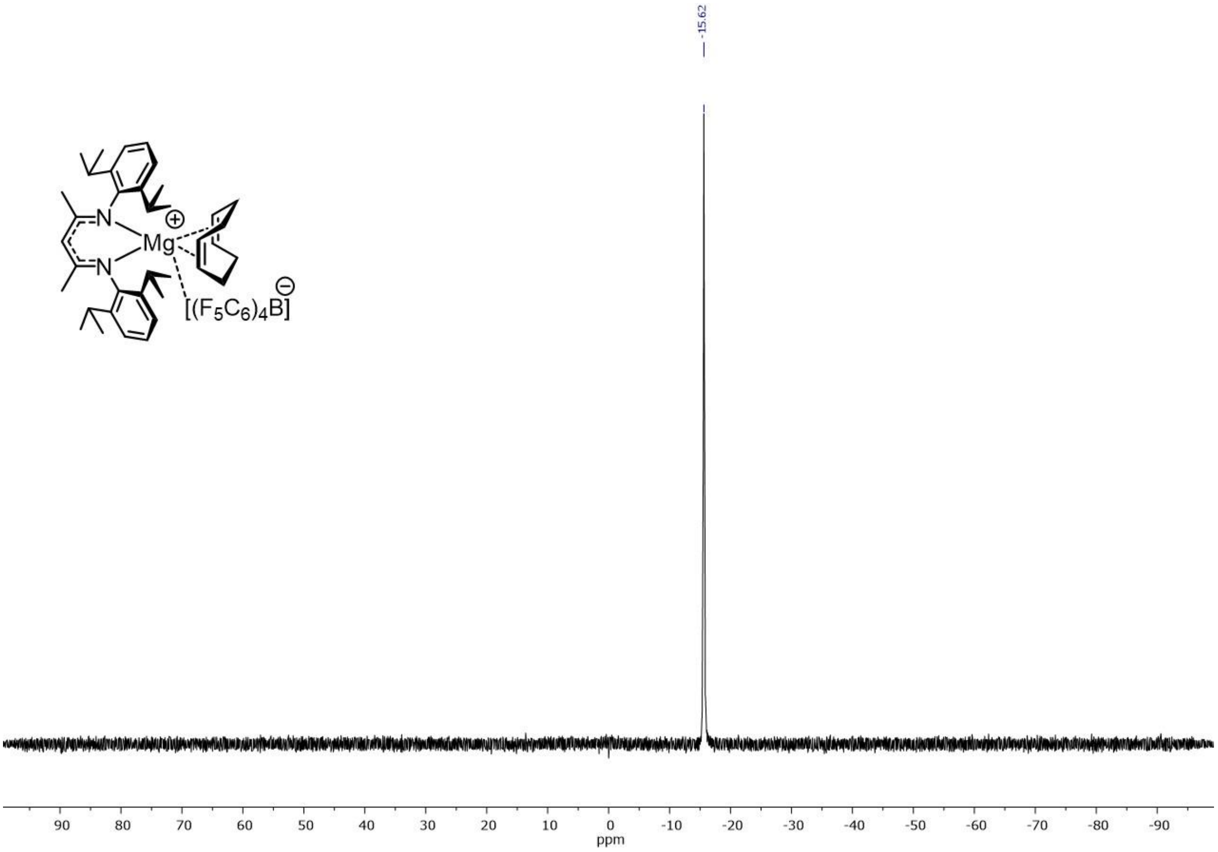

Figure S29: $^{11}B\{^{1}H\}$ NMR spectrum (128 MHz, 298 K) of $[(^{Me}BDI)Mg^{+}(\textit{cod})][B(C_6F_5)_4^{-}]$ in C_6D_5Br.

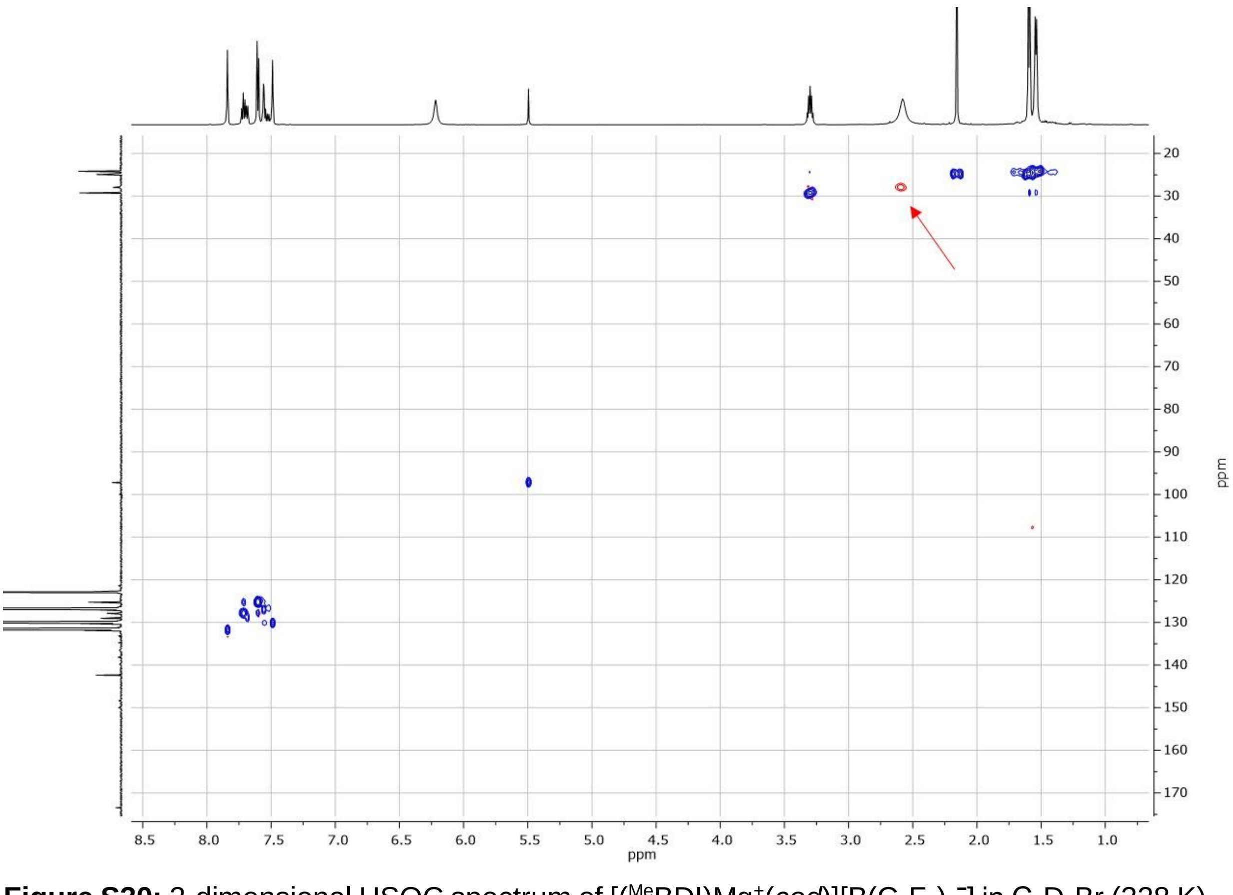

Figure S30: 2-dimensional HSQC spectrum of $[(^{Me}BDI)Mg^{+}(\textit{cod})][B(C_6F_5)_4^{-}]$ in C_6D_5Br (328 K).

1.1.6 Spectra of [(^{Me}BDI)Mg$^+$(*cht*)][B(C_6F_5)$_4^-$] (5)

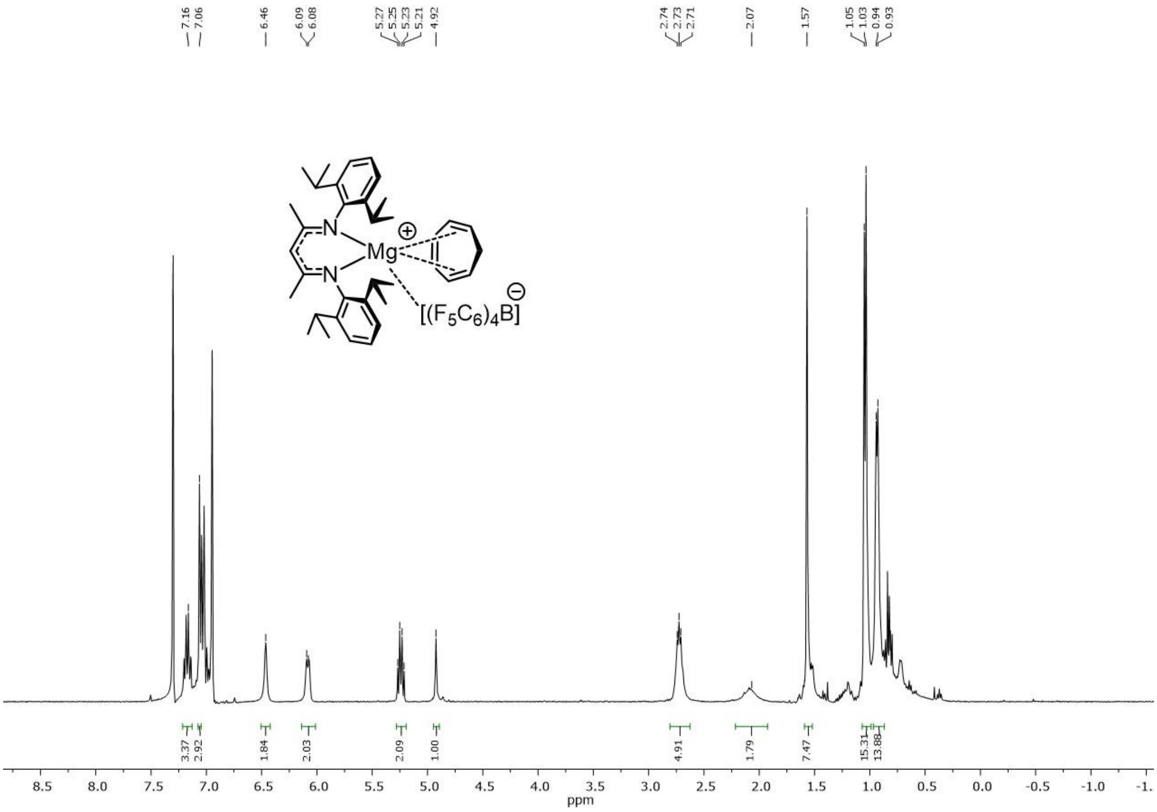

Figure S31: ^{1}H NMR spectrum (400 MHz, 298 K) of [(^{Me}BDI)Mg$^+$(*cht*)][B(C_6F_5)$_4^-$] in C_6D_5Br. Alkene chemical shifts do not correspond to shifts for free alkene. Signals are broadened *cht* partially bound in solution.

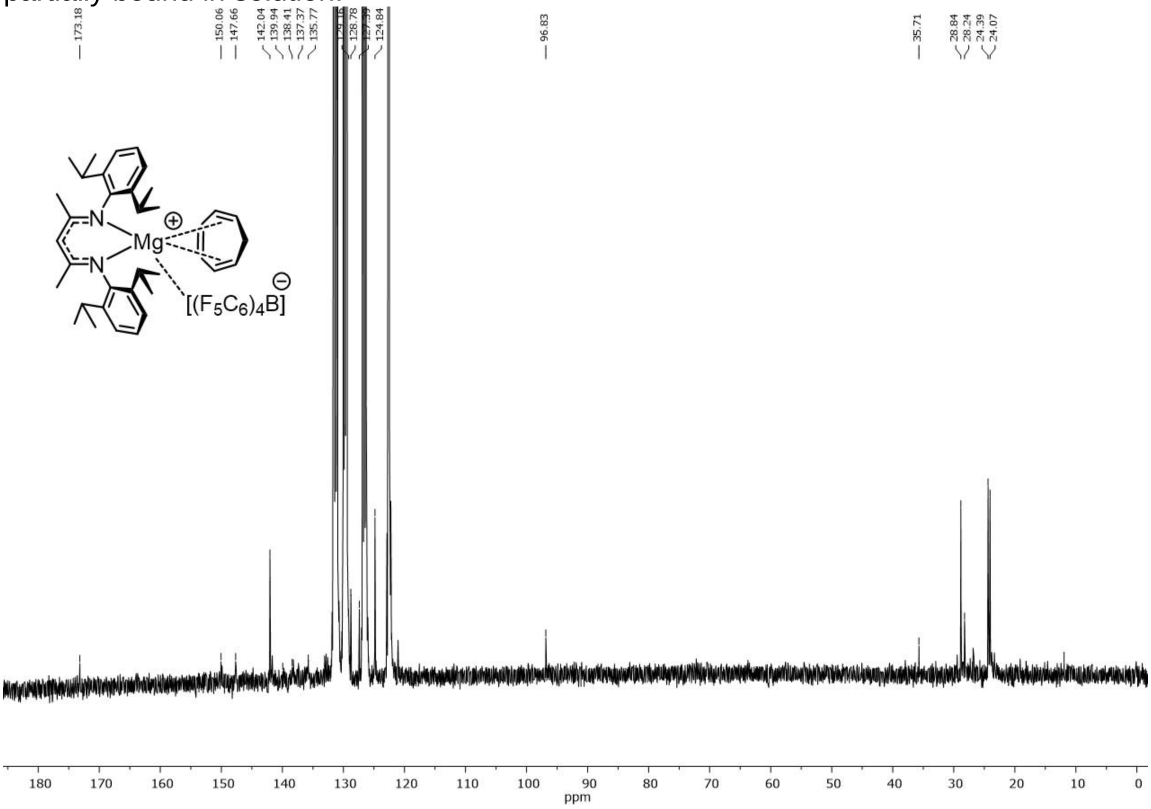

Figure S32: ^{13}C{^{1}H} NMR spectrum (101 MHz, 298 K) of [(^{Me}BDI)Mg$^+$(*cht*)][B(C_6F_5)$_4^-$] in C_6D_5Br.

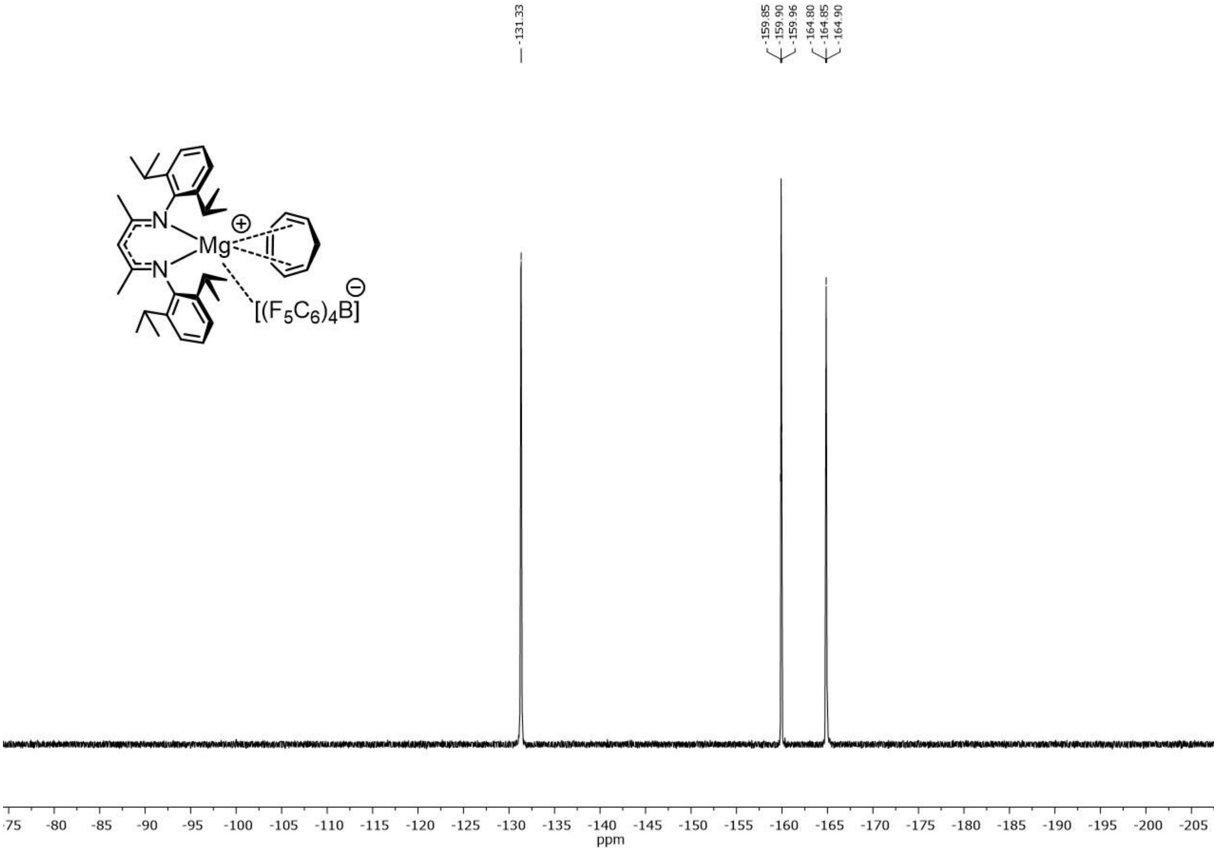

Figure S33: $^{19}F\{^1H\}$ NMR spectrum (376 MHz, 298 K) of [(^{Me}BDI)Mg^+(*cht*)][$B(C_6F_5)_4^-$] in C_6D_5Br.

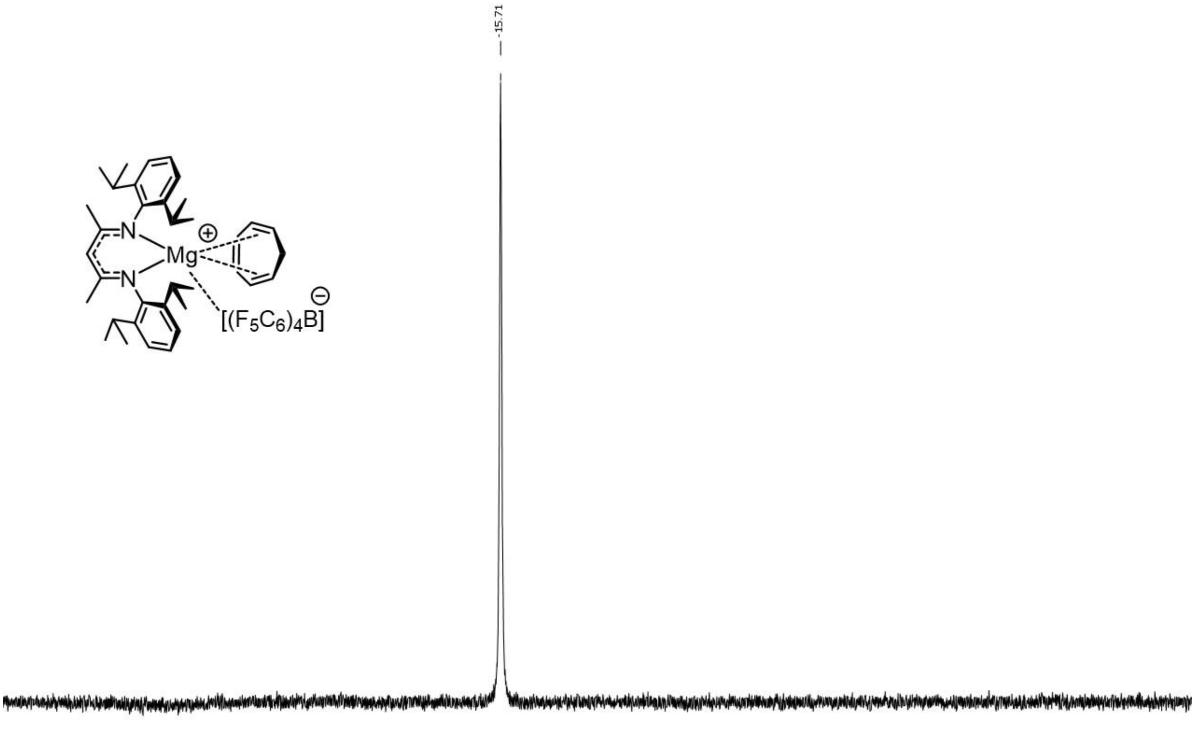

Figure S34: $^{11}B\{^1H\}$ NMR spectrum (128 MHz, 298 K) of [(^{Me}BDI)Mg^+(*cht*)][$B(C_6F_5)_4^-$] in C_6D_5Br.

1.1.7 Spectra of [(^{Me}BDI)Mg$^+$(*dmbd*)][B(C_6F_5)$_4^-$] (6)

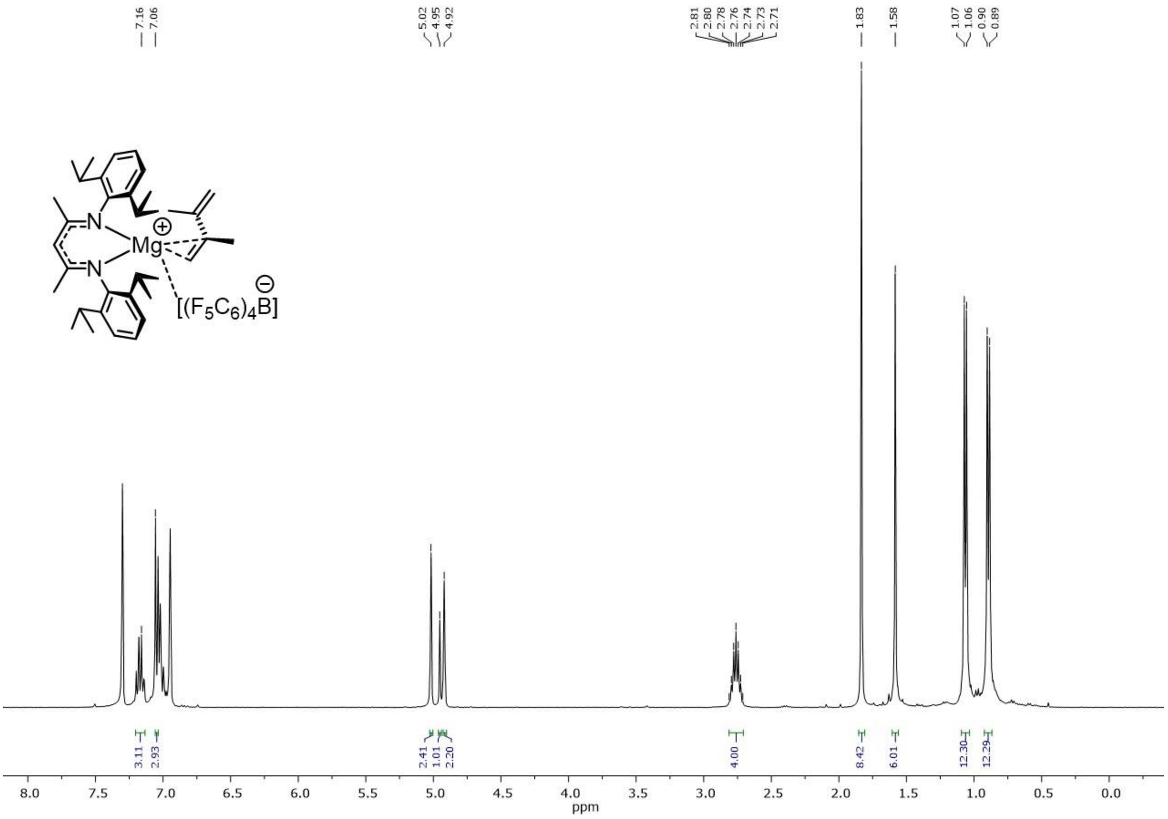

Figure S35: ^{1}H NMR spectrum (600 MHz, 298 K) of [(^{Me}BDI)Mg$^+$(*dmbd*)][B(C_6F_5)$_4^-$] in C_6D_5Br. Alkene chemical shifts correspond to shifts for free alkene. Signals are sharp → no coordination in solution.

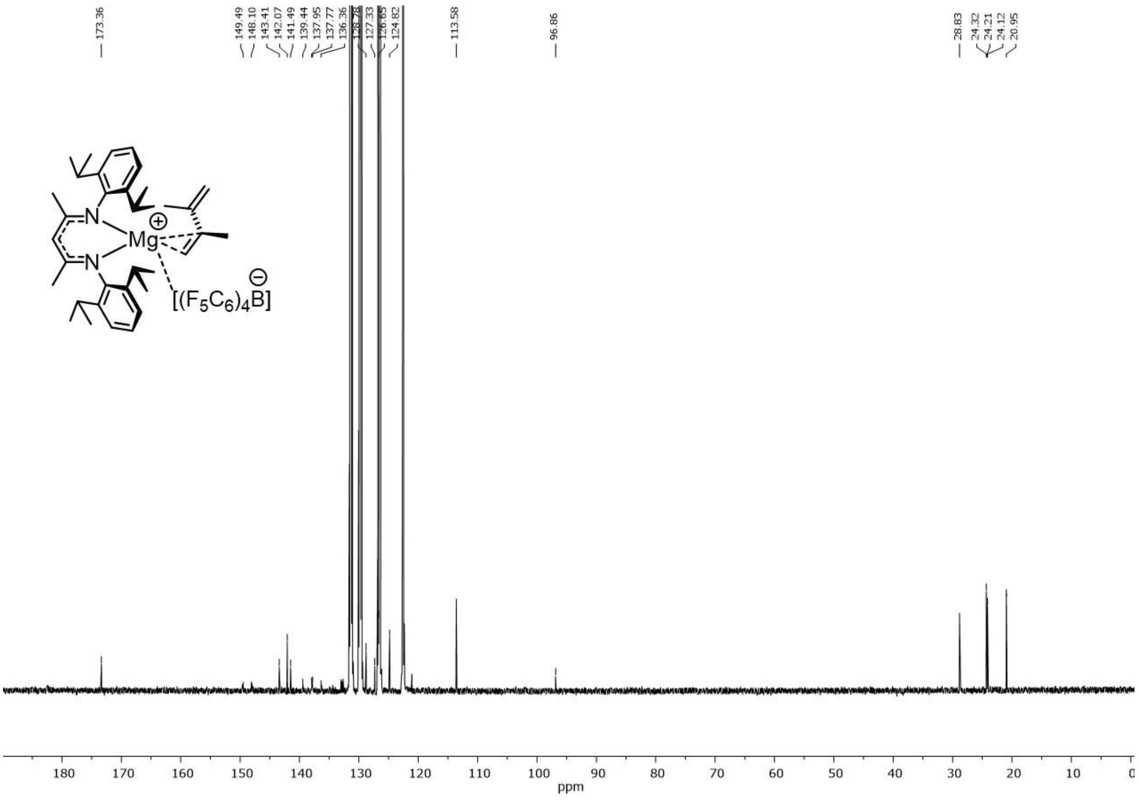

Figure S36: ^{13}C{^{1}H} NMR spectrum (151 MHz, 298 K) of [(^{Me}BDI)Mg$^+$(*dmbd*)][B(C_6F_5)$_4^-$] in C_6D_5Br.

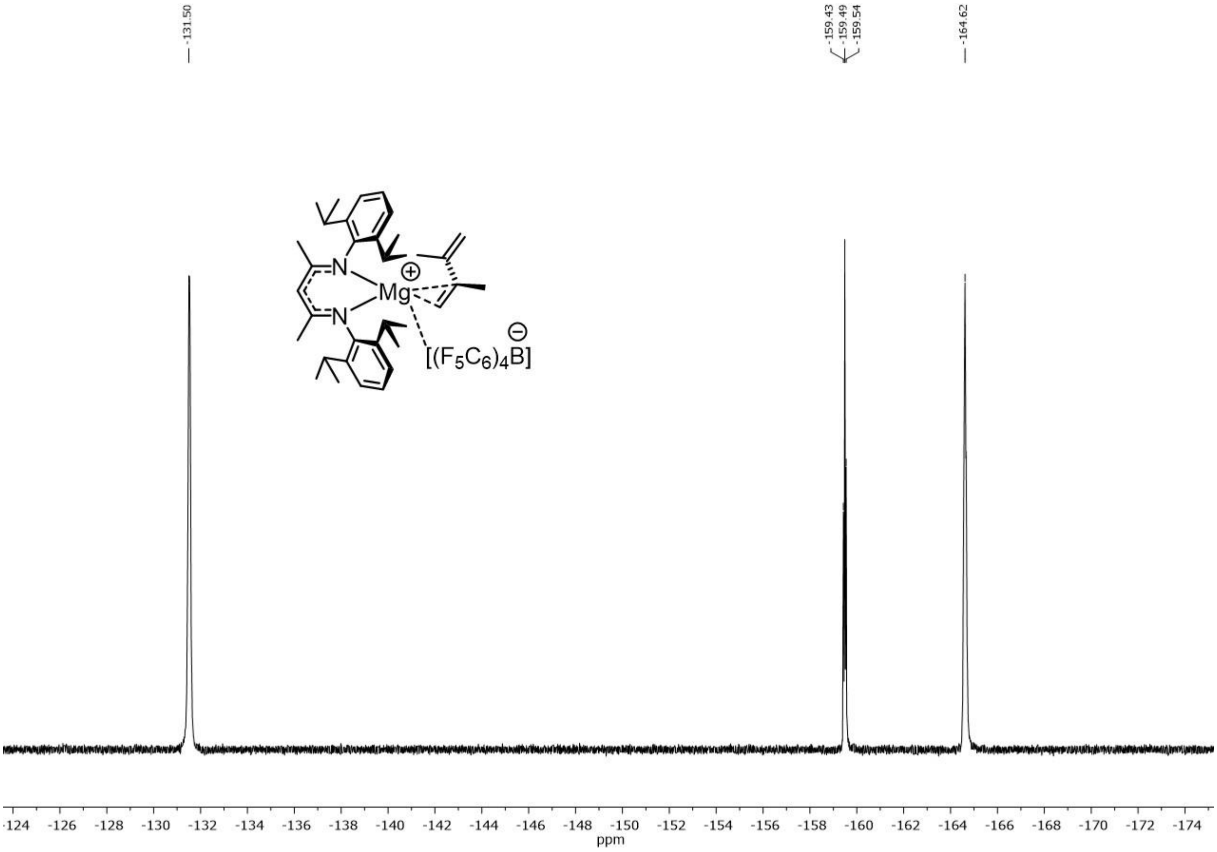

Figure S37: $^{19}F\{^1H\}$ NMR spectrum (376 MHz, 298 K) of $[(^{Me}BDI)Mg^+$(*dmbd*)]$[B(C_6F_5)_4^-]$ in C_6D_5Br.

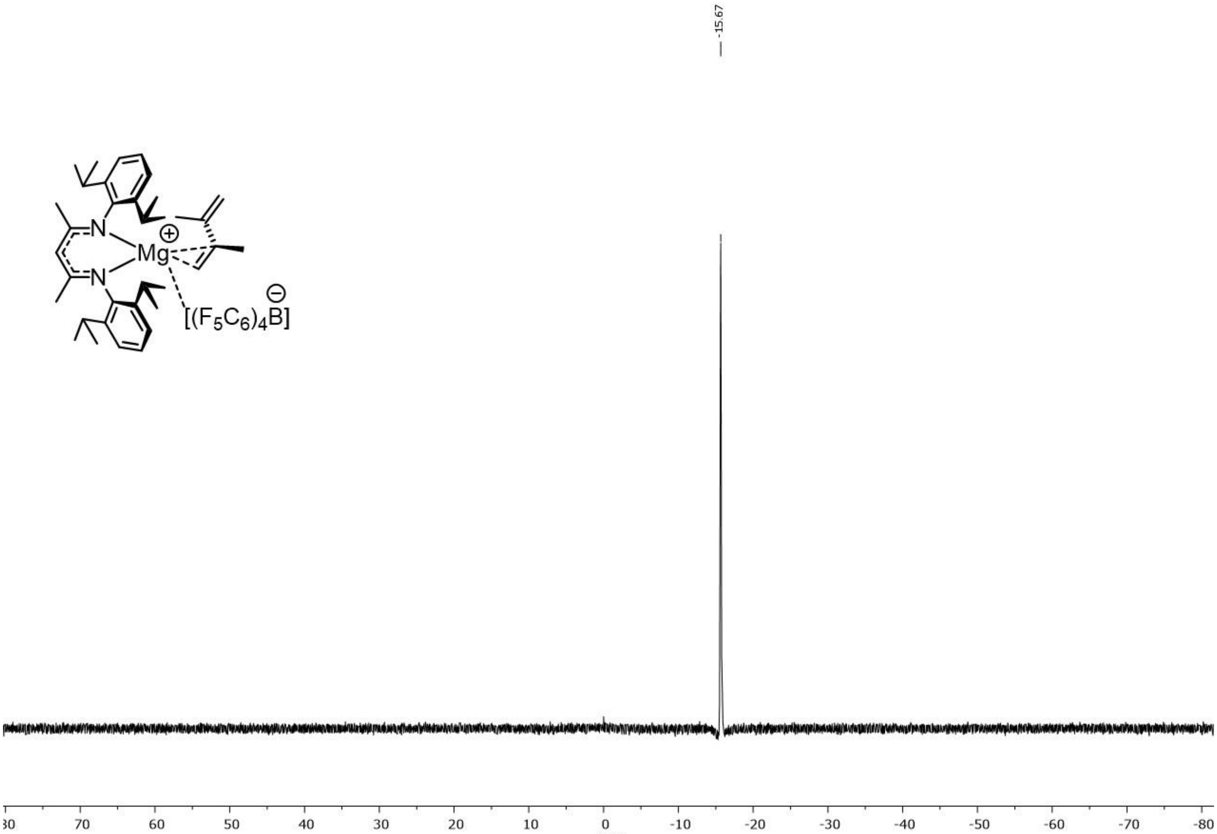

Figure S38: $^{11}B\{^1H\}$ NMR spectrum (128 MHz, 298 K) of $[(^{Me}BDI)Mg^+$(*dmbd*)]$[B(C_6F_5)_4^-]$ in C_6D_5Br.

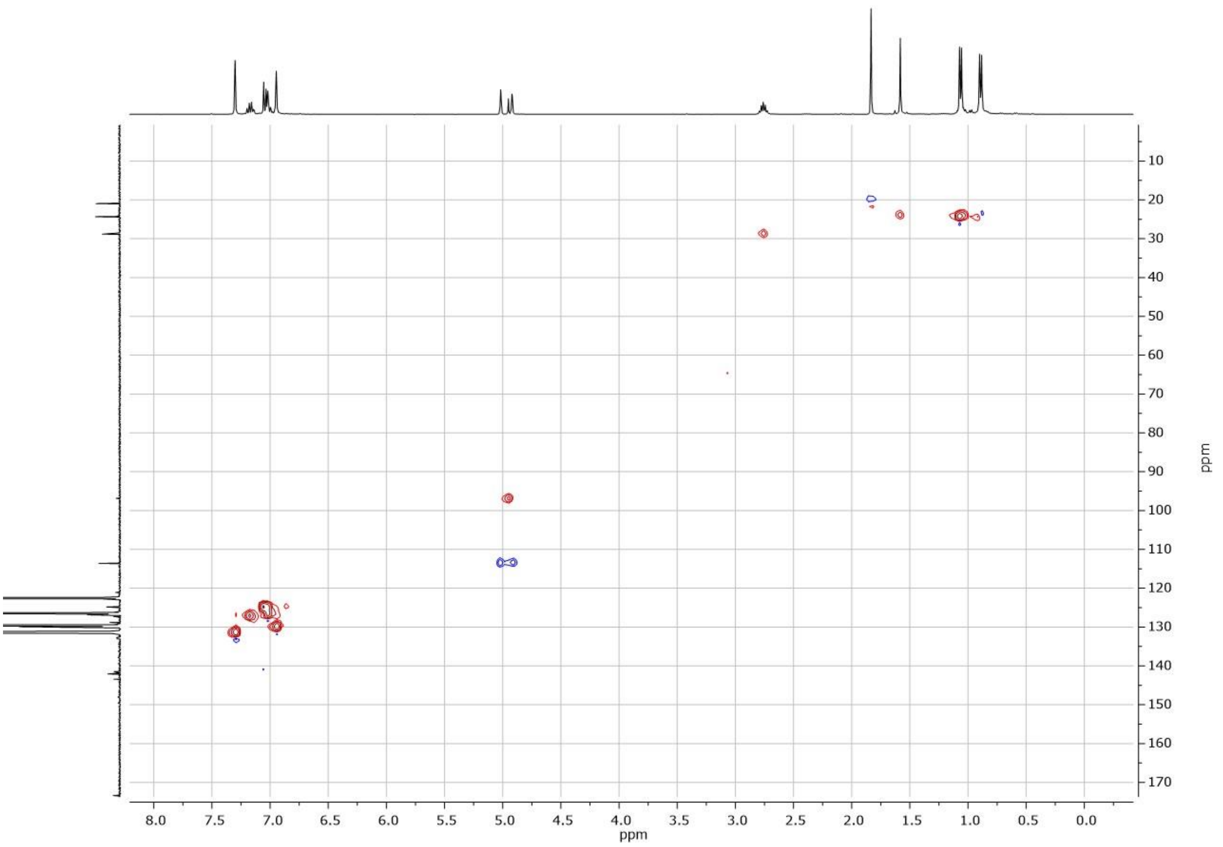

Figure S39: 2-dimensional HSQC spectrum of [(^{Me}BDI)Mg^{+}(*dmbd*)][B(C_6F_5)$_4^-$] in C_6D_5Br.

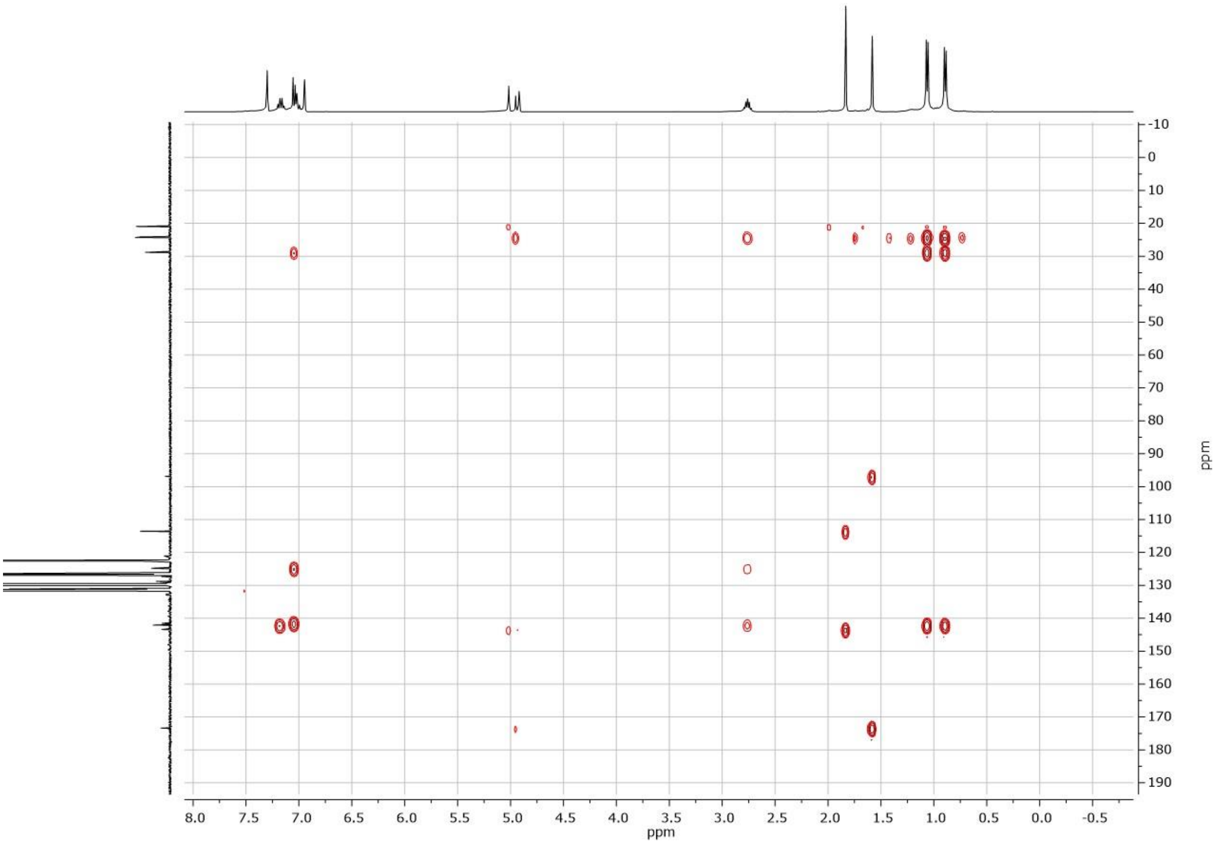

Figure S40: 2-dimensional HMBC spectrum of [(^{Me}BDI)Mg^{+}(*dmbd*)][B(C_6F_5)$_4^-$] in C_6D_5Br.

1.1.8 Spectra of [(^{Me}BDI)Mg$^+$(*eb*)][B(C_6F_5)$_4^-$] (7)

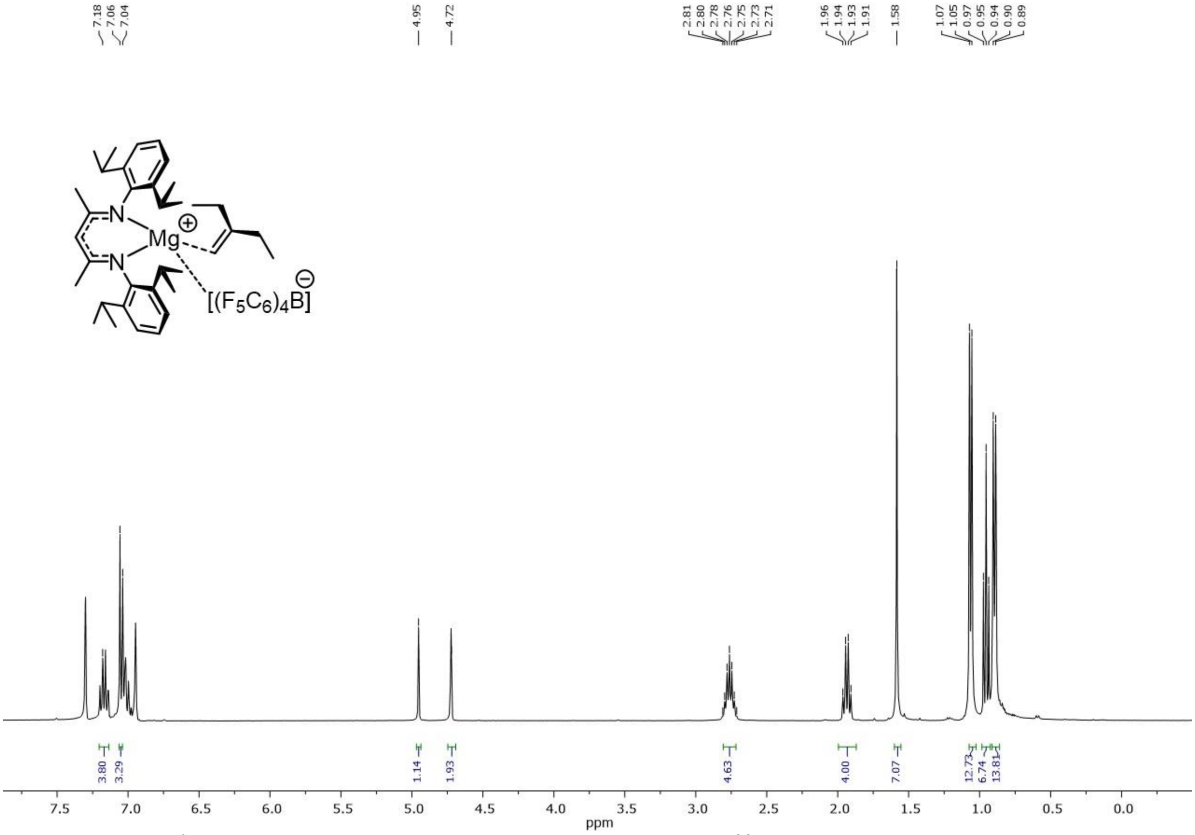

Figure S41: ^{1}H NMR spectrum (400 MHz, 298 K) of [(^{Me}BDI)Mg$^+$(*eb*)][B(C_6F_5)$_4^-$] in C_6D_5Br. Alkene chemical shifts correspond to shifts for free alkene. Signals are sharp → no coordination in solution.

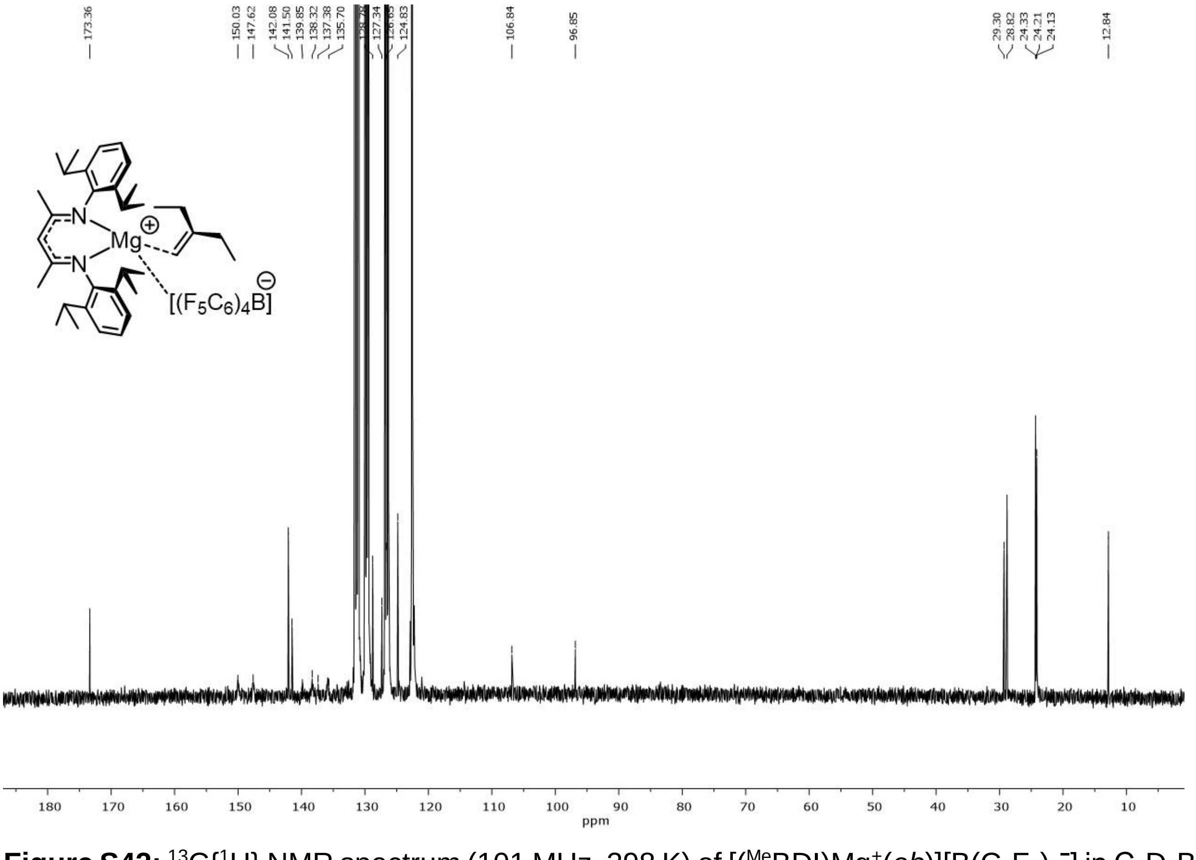

Figure S42: ^{13}C{^{1}H} NMR spectrum (101 MHz, 298 K) of [(^{Me}BDI)Mg$^+$(*eb*)][B(C_6F_5)$_4^-$] in C_6D_5Br.

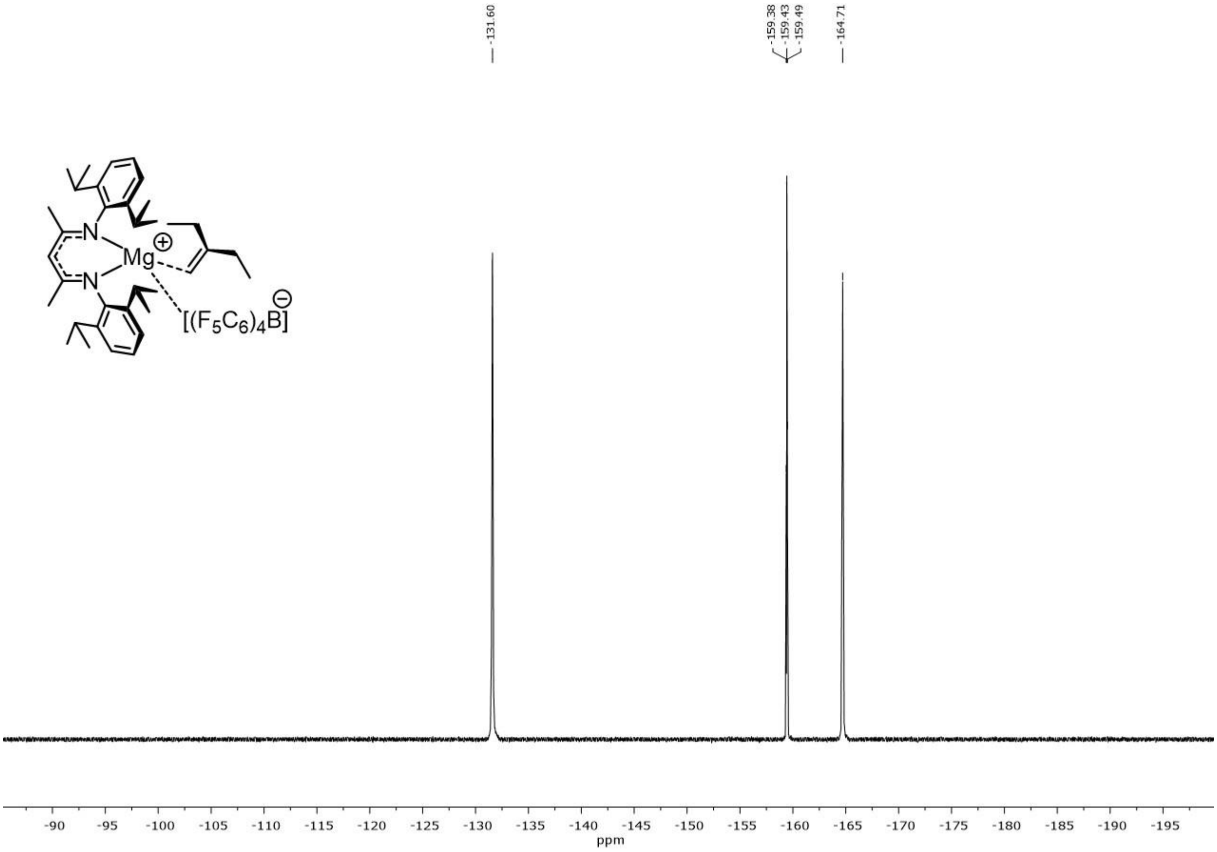

Figure S43: $^{19}F\{^1H\}$ NMR spectrum (376 MHz, 298 K) of $[(^{Me}BDI)Mg^+(eb)][B(C_6F_5)_4^-]$ in C_6D_5Br.

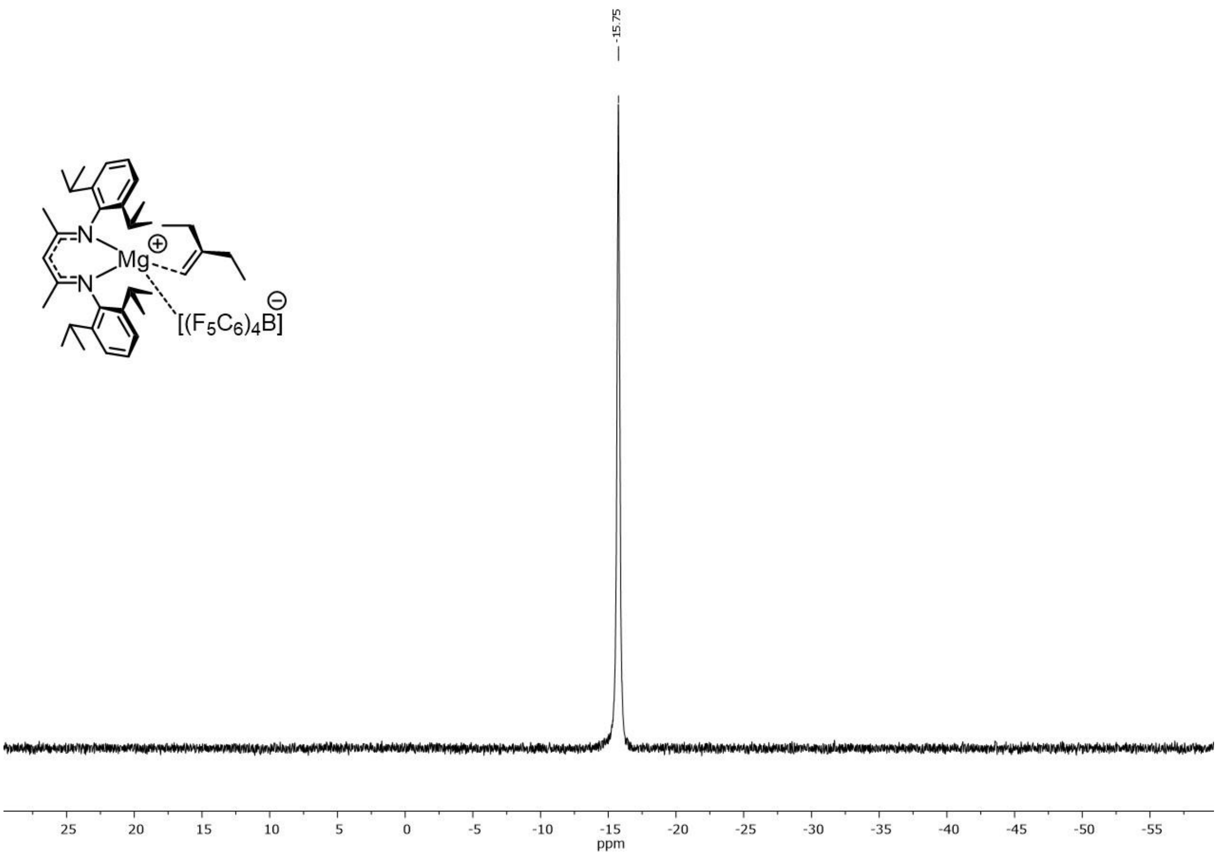

Figure S44: $^{11}B\{^1H\}$ NMR spectrum (128 MHz, 298 K) of $[(^{Me}BDI)Mg^+(eb)][B(C_6F_5)_4^-]$ in C_6D_5Br.

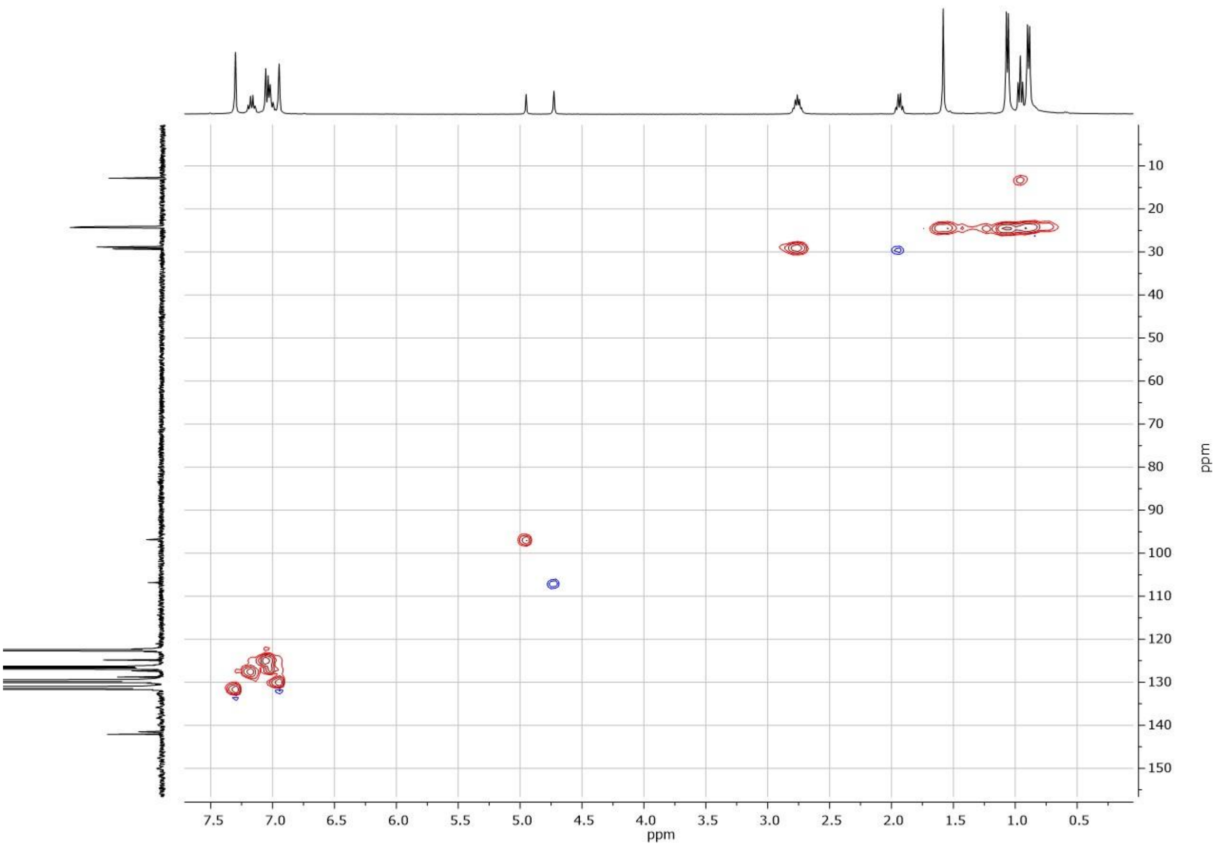

Figure S45: 2-dimensional HSQC spectrum of [(^{Me}BDI)Mg^{+}(*eb*)][B(C_6F_5)$_4$$^{-}$] in C_6D_5Br.

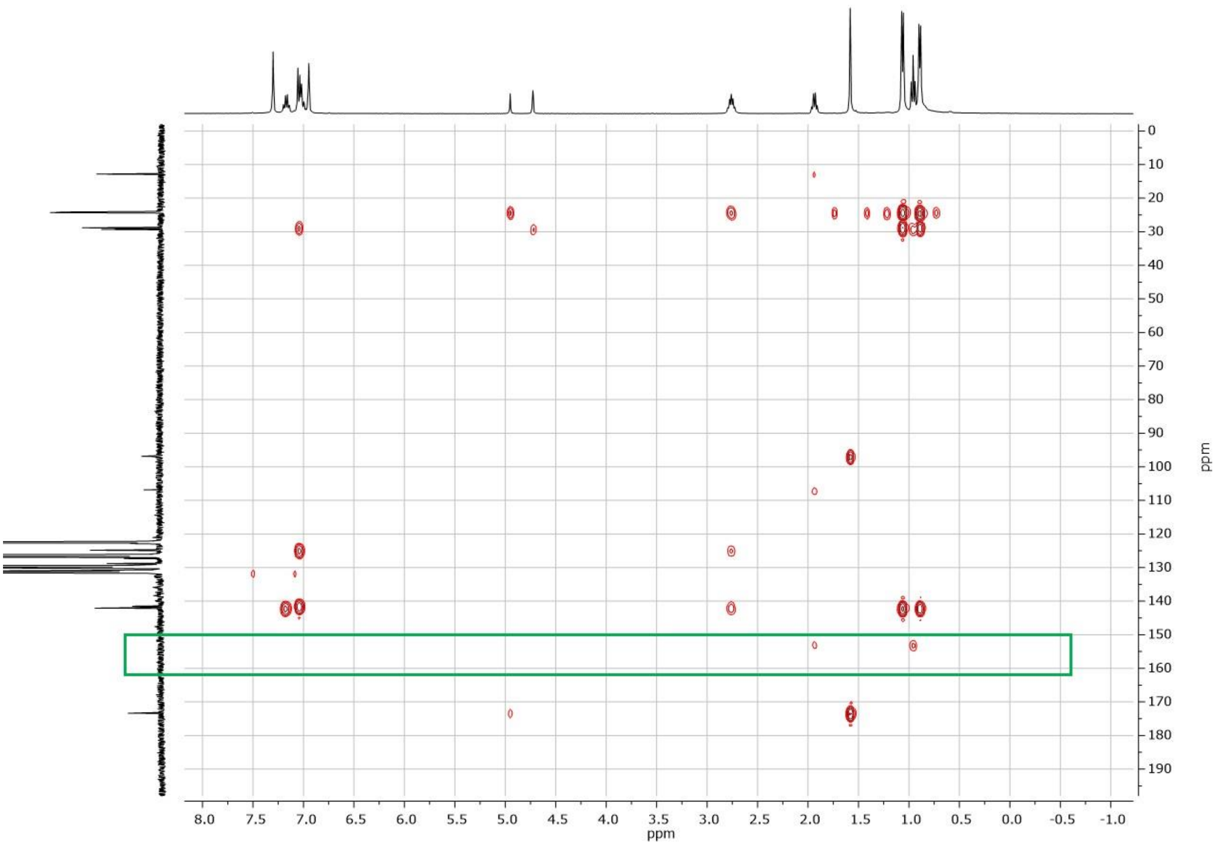

Figure S46: 2-dimensional HMBC spectrum of [(^{Me}BDI)Mg^{+}(*eb*)][B(C_6F_5)$_4$$^{-}$] in C_6D_5Br.

1.2 Single Crystal X-Ray Diffraction

1.2.1. Structure determination of [(^{tBu}BDI)Mg^{+}(*divinylsiloxane*)][B(C_6F_5)$_4^{-}$] (1)

A colorless crystal of compound [($^{t\mathrm{Bu}}$BDI)Mg^{+}(*divinylsiloxane*)][B(C_6F_5)$_4^{-}$] was embedded in inert perfluoropolyalkylether (viscosity 1800 cSt; ABCR GmbH) and mounted using a Hampton Research CryoLoop. The crystal was then flash cooled to 153.0(1) K in a nitrogen gas stream and kept at this temperature during the experiment. The crystal structure was measured on a SuperNova diffractometer with Atlas detector using a CuKα microfocus source. The measured data was processed with the CrysAlisPro (v39.46) software package.[S1] Using Olex2,[S3] the structure was solved with the ShelXT[S4] structure solution program using Intrinsic Phasing and refined with the ShelXL[S5] refinement package using Least Squares minimization. All non-hydrogen atoms were refined anisotropically. Most hydrogen atoms were placed in ideal positions and refined as riding atoms with relative isotropic displacement parameters. The positions of the hydrogen atoms at C1 and C2 were observed from difference Fourier maps and refined. Crystallographic and refinement data are summarized in Table S1 (see below).

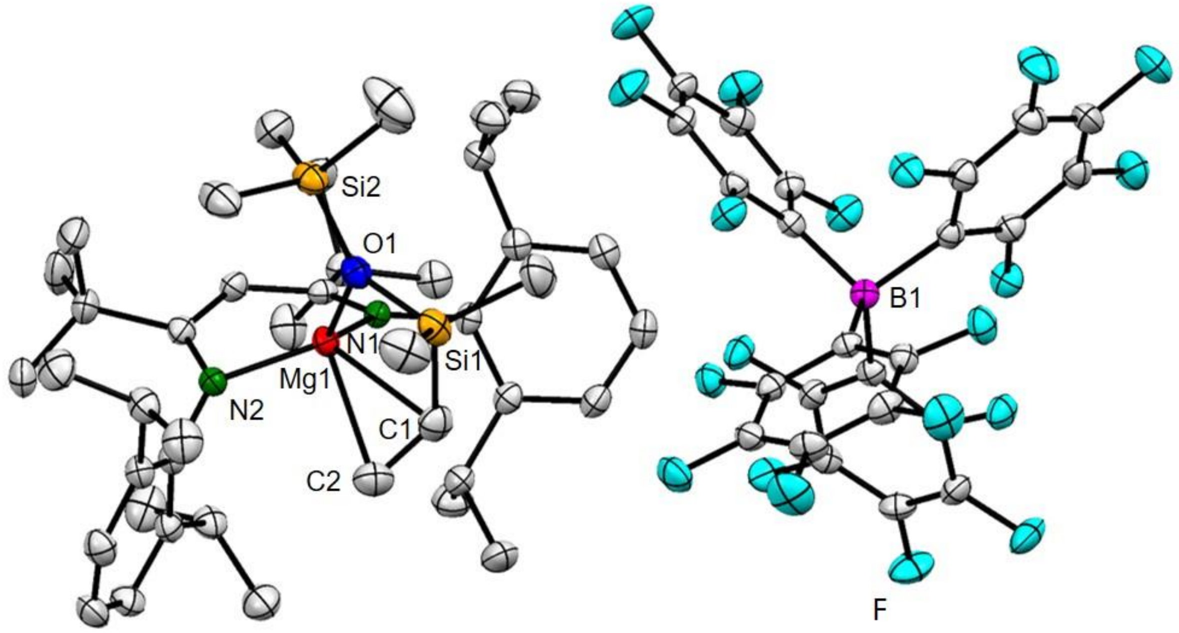

Figure S47: ORTEP representation of [($^{t\mathrm{Bu}}$BDI)Mg^{+}(*divinylsiloxane*)][B(C_6F_5)$_4^{-}$] (**1**) with non-coordinated counter anion (probability level 50%). Hydrogen atoms were omitted for clarity.

1.2.2. Structure determination of [(^{tBu}BDI)Mg^{+}(*cod*)][B(C_6F_5)$_4^{-}$] (2)

A colorless crystal of the composition [($^{t\mathrm{Bu}}$BDI)Mg^{+}(*cod*)][B(C_6F_5)$_4^{-}$] was embedded in inert perfluoropolyalkylether (viscosity 1800 cSt; ABCR GmbH) and mounted using a Hampton Research CryoLoop. The crystal was then flash cooled to 100.0(2) K in a nitrogen gas stream and kept at this temperature during the experiment. The crystal structure was measured on a SuperNova diffractometer with Atlas S2 detector using a CuKα microfocus source. The measured data was processed with the CrysAlisPro (v40.67a) software package.[S15] Using

Olex2,[S3] the structure was solved with the ShelXT[S4] structure solution program using Intrinsic Phasing and refined with the ShelXL[S5] refinement package using Least Squares minimization. All non-hydrogen atoms were refined anisotropically. Most hydrogen atoms were placed in ideal positions and refined as riding atoms with relative isotropic displacement parameters. The positions of the hydrogen atoms at C1 and C2 were observed from difference Fourier maps and refined.

A residual electron density peak close to C2 most likely stems from a substitutional disorder of the *cod* ligand and bromobenzene. Bromobenzene (coordinated *via* the Br atom) is present only in approximately 1.5% of the molecules, therefore only electron density related to the position of the Br atom is observed. Since it was not possible to build a suitable disorder model, this disorder was ignored. Nevertheless, the residual electron density related to the bromine position slightly affects the position of C2 as well as its thermal ellipsoid, which becomes elongated. In order to counter this effect, an ISOR restraint was placed on C2. Crystallographic and refinement data are summarized in Table S1 (see below).

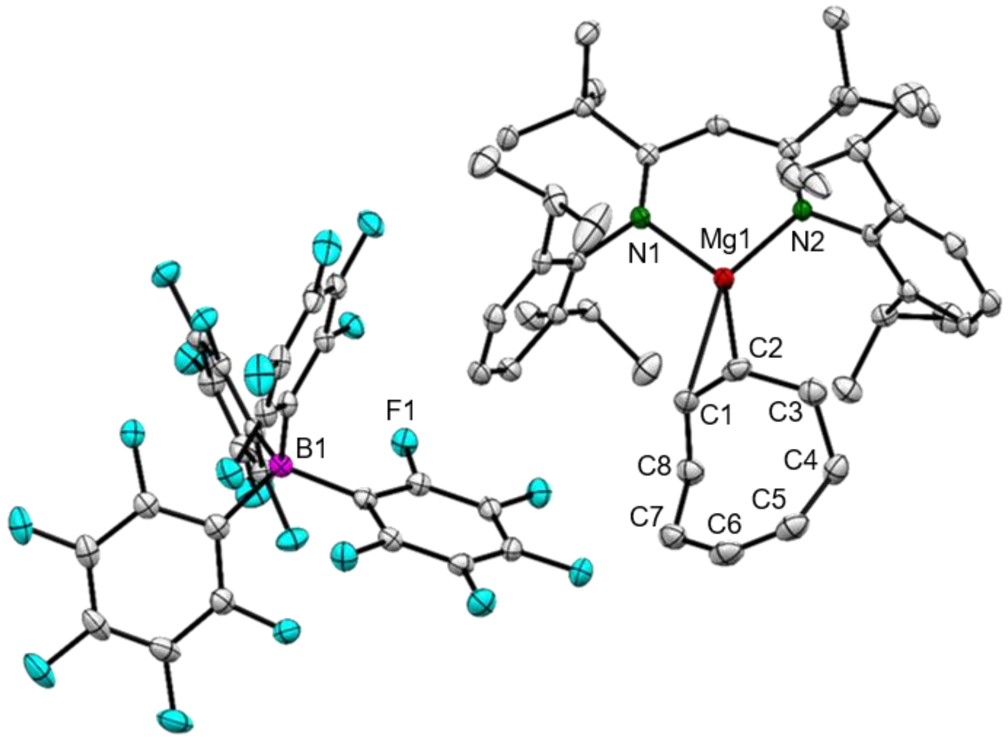

Figure S48: ORTEP representation of [(^{tBu}BDI)Mg$^+$(*cod*)][B(C_6F_5)$_4$$^-$] (**2**) with non-coordinated counter anion (probability level 50%). Hydrogen atoms were omitted for clarity.

1.2.3. Structure determination of [(^{tBu}BDI)Mg$^+$(*coe*)][B(C_6F_5)$_4^-$] (3)

A colorless crystal of compound [^{tBu}BDIMg$^+$(*coe*)][B(C_6F_5)$_4^-$] was embedded in inert perfluoropolyalkylether (viscosity 1800 cSt; ABCR GmbH) and mounted using a Hampton Research CryoLoop. The crystal was then flash cooled to 100.0(2) K in a nitrogen gas stream and kept at this temperature during the experiment. The crystal structure was measured on a SuperNova diffractometer with Atlas S2 detector using a CuKα microfocus source. The measured data was processed with the CrysAlisPro (v40.67a) software package.[S15] Using Olex2,[S3] the structure was solved with the ShelXT[S4] structure solution program using Intrinsic Phasing and refined with the ShelXL[S5] refinement package using Least Squares Minimization. All non-hydrogen atoms were refined anisotropically. Most hydrogen atoms were placed in ideal positions and refined as riding atoms with relative isotropic displacement parameters. The positions of the hydrogen atoms connected to C1 and C2 were observed from difference Fourier maps and refined.

Disorder of two *i*Pr groups and of the *coe* ligand was observed. The disorder was modeled with the help of similarity restraints (SIMU) and rigid bond restraints (RIGU).[S6] The relative occupancies of the two alternative orientations of the *i*Pr groups were refined to 0.745(6)/0.255(6) (*i*Pr 1) and 0.67(2)/0.33(2) (*i*Pr 2), respectively.

The disorder of the *coe* ligand is more complicated. What looked like a simple overlap of 2 different conformers at first glance, turned out to involve at least 3 conformers, which overlap in different parts of the eight-membered ring. C4 und C7 belong to all 3 conformers. Conformer 1 and 2 additionally overlap in C1, C2, C3 and C4, while conformer 2 and 3 additionally overlap in C5A and C6A. C5 and C6 belong exclusively to conformer 1, and C1A, C2A, C3A and C8A to conformer 3. The site occupancy factors of the three conformers are 0.580(3) (conformer 1), 0.330(3) (conformer 2) and 0.090(2) (conformer 3). Crystallographic and refinement data are summarized in Table S1 (see below).

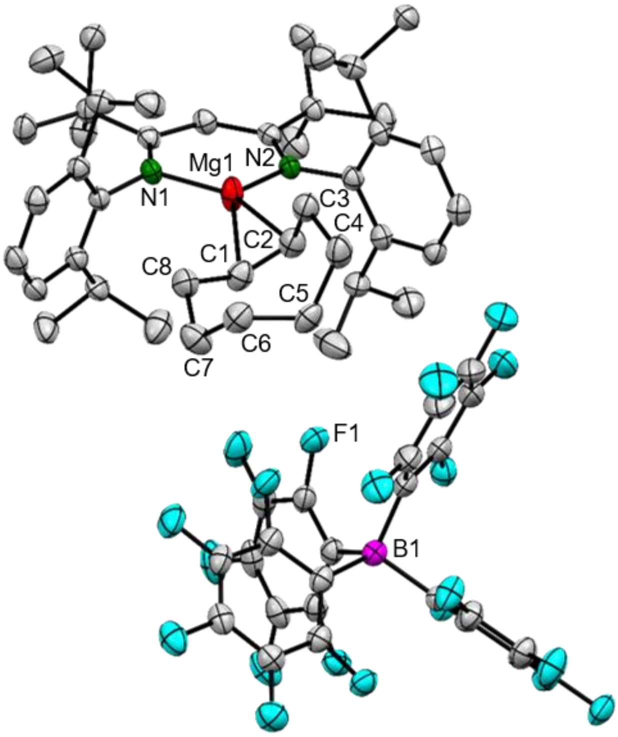

Figure S49: ORTEP representation of [(^{tBu}BDI)Mg^{+}(*coe*)][B(C_6F_5)$_4^{-}$] (**3**) with non-coordinated counter anion (probability level 50%). Hydrogen atoms were omitted for clarity.

1.2.4 Structure determination of [(^{Me}BDI)Mg^{+}(*cod*)][B(C_6F_5)$_4^{-}$] (4)

A colorless crystal of compound [(^{Me}BDI)Mg^{+}(*cod*)][B(C_6F_5)$^{-}$] was embedded in inert perfluoropolyalkylether (viscosity 1800 cSt; ABCR GmbH) and mounted using a Hampton Research CryoLoop. The crystal was then flash cooled to 100.0(1) K in a nitrogen gas stream and kept at this temperature during the experiment. The crystal structure was measured on a SuperNova diffractometer with Atlas S2 detector using a CuKα microfocus source. The measured data was processed with the CrysAlisPro (v39.46) software package.[S1] Using Olex2,[S3] the structure was solved with the ShelXT[S4] structure solution program using Intrinsic Phasing and refined with the ShelXL[S5] refinement package using Least Squares minimization. The asymmetric unit contained five crystallographically independent molecules. All non-hydrogen atoms were refined anisotropically. All hydrogen atoms were placed in ideal positions and refined as riding atoms with relative isotropic displacement parameters.

Disorder of one DIPP group and one C_6F_5 group moiety was observed and was modeled with the help of similarity restraints (SIMU, SADI) and rigid bond restraints (RIGU). [S6] The relative occupancies of the two alternative orientations were refined to 0.533(12)/ 0.467(12) (dipp), and 0.56(3)/0.44(3) (C_6F_5), respectively. Crystallographic and refinement data are summarized in Table S1 (see below).

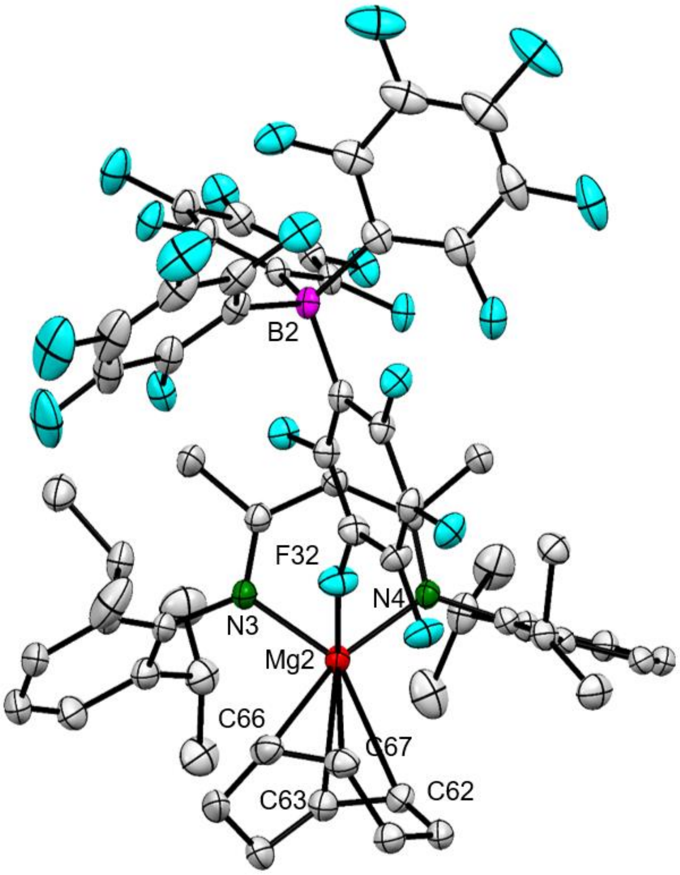

Figure S50: ORTEP representation of [(^{Me}BDI)Mg^{+}(*cod*)][B(C_6F_5)$_4^{-}$] (**4a**) with coordinated counter anion (probability level 50%). Hydrogen atoms were omitted for clarity. One of three symmetry independent molecules with coordinated counter anion is shown. Selected bond lengths (Å): Mg2-N3 2.0235(19), Mg2-N4 2.0021(19), Mg2-C62 2.618(2), Mg2-C63 2.648(2), Mg2-C66 2.514(2), Mg2-C67 2.695(2), Mg2-F32 2.4543(15), C62-C63 1.336(3), C66-C67 1.341(4).

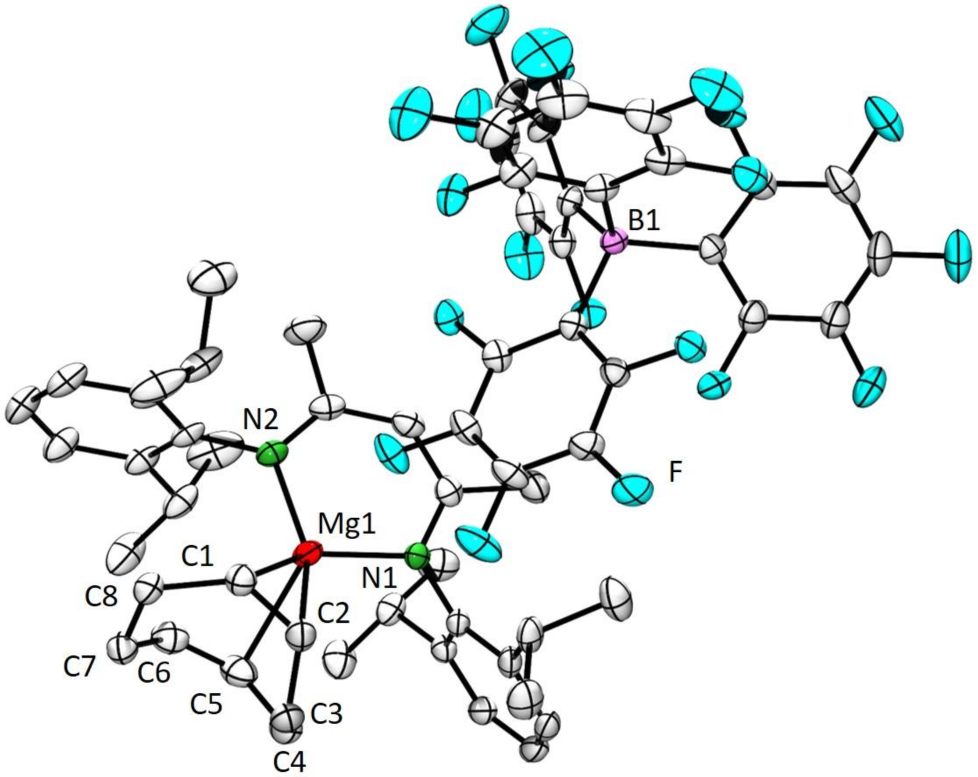

Figure S51: ORTEP representation of [(^{Me}BDI)Mg^{+}(*cod*)][B(C_6F_5)$_4$$^{-}$] (**4b**) with non-coordinated counter anion (probability level 50%). Hydrogen atoms were omitted for clarity. One of two symmetry independent molecules with non-coordinated counter anion is shown. Selected bond lengths (Å): Mg1-N1 1.9967(19), Mg1-N2 1.9905(19), Mg1-C1 2.502(2), Mg1-C2 2.508(2), Mg1-C5 2.494(3), Mg1-C6 2.746(3), C1-C2 1.354(3), C5-C6 1.331(4).

1.2.5 Structure determination of [(^{Me}BDI)Mg^{+}(*cht*)][B(C_6F_5)$_4$$^{-}$] (5)

A colorless crystal of compound [(^{Me}BDI)Mg^{+}(*cht*)][B(C_6F_5)$_4$$^{-}$] was embedded in inert perfluoropolyalkylether (viscosity 1800 cSt; ABCR GmbH) and mounted using a Hampton Research CryoLoop. The crystal was then flash cooled to 100.0(1) K in a nitrogen gas stream and kept at this temperature during the experiment. The crystal structure was measured on a SuperNova diffractometer with Atlas S2 detector using a CuKα microfocus source. The measured data was processed with the CrysAlisPro (v40.18b) software package.[S2] Using Olex2,[S3] the structure was solved with the ShelXT[S4] structure solution program using Intrinsic Phasing and refined with the ShelXL[S5] refinement package using Least Squares minimization. All non-hydrogen atoms were refined anisotropically. Most hydrogen atoms were placed in ideal positions and refined as riding atoms with relative isotropic displacement parameters. The positions of the hydrogen atoms of the *cht* ligand were observed from difference Fourier maps and refined with the help of appropriate SADI restraints.

Additionally, voids with heavily disordered solvent (mixture of *cht*/*n*-pentane) were found within the crystal. A suitable disorder model for these solvent molecules could not be built. Therefore, their contribution to the structure factors was secured by back-Fourier transformation using the

solvent mask routine[S7,S8] of the program Olex2.[S3] The solvent accessible voids treated this way had a size of 922.4 Å^3 (15.2% of the unit cell) and contained 208.7 electrons/unit cell. Crystallographic and refinement data are summarized in Table S1 (see below).

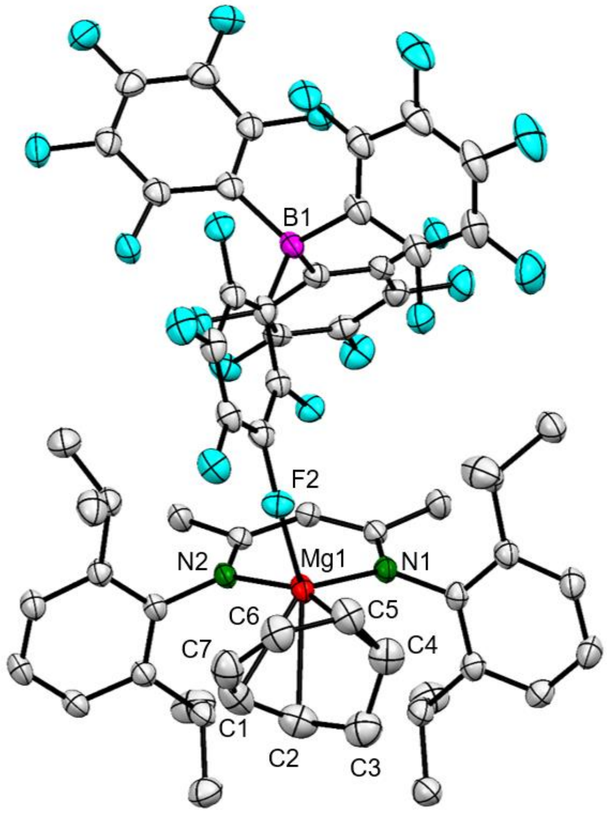

Figure S52: ORTEP representation of [(^{Me}BDI)Mg$^+$(*cht*)][B(C_6F_5)$_4^-$] (**5**) with coordinated counter anion (probability level 50%). Hydrogen atoms were omitted for clarity.

1.2.6 Structure determination of [(^{Me}BDI)Mg$^+$(*dmbd*)][B(C_6F_5)$_4^-$] (6)

A colorless crystal of compound [(^{Me}BDI)Mg$^+$(*dmbd*)][B(C_6F_5)$_4^-$] was embedded in inert perfluoropolyalkylether (viscosity 1800 cSt; ABCR GmbH) and mounted using a Hampton Research CryoLoop. The crystal was then flash cooled to 100.0(1) K in a nitrogen gas stream and kept at this temperature during the experiment. The crystal structure was measured on a SuperNova diffractometer with Atlas S2 detector using a CuKα microfocus source. The measured data was processed with the CrysAlisPro (v40.18b) software package.[S2] Using Olex2,[S3] the structure was solved with the ShelXT[S4] structure solution program using Intrinsic Phasing and refined with the ShelXL[S5] refinement package using Least Squares minimization. All non-hydrogen atoms were refined anisotropically. Most hydrogen atoms were placed in ideal positions and refined as riding atoms with relative isotropic displacement parameters. The positions of the hydrogen atoms at C1 and C9 were observed from difference Fourier maps and refined.

Additionally, voids with heavily disordered solvent (mixture of chlorobenzene/*n*-hexane) were found within the crystal. A suitable disorder model for these solvent molecules could not be built. Therefore, their contribution to the structure factors was secured by back-Fourier transformation using the solvent mask routine[S7,S8] of the program Olex2.[S3] The solvent accessible voids treated this way had a size of 953.2 Å^3 (15.5% of the unit cell) and contained 235.1 electrons/unit cell. Crystallographic and refinement data are summarized in Table S1 (see below).

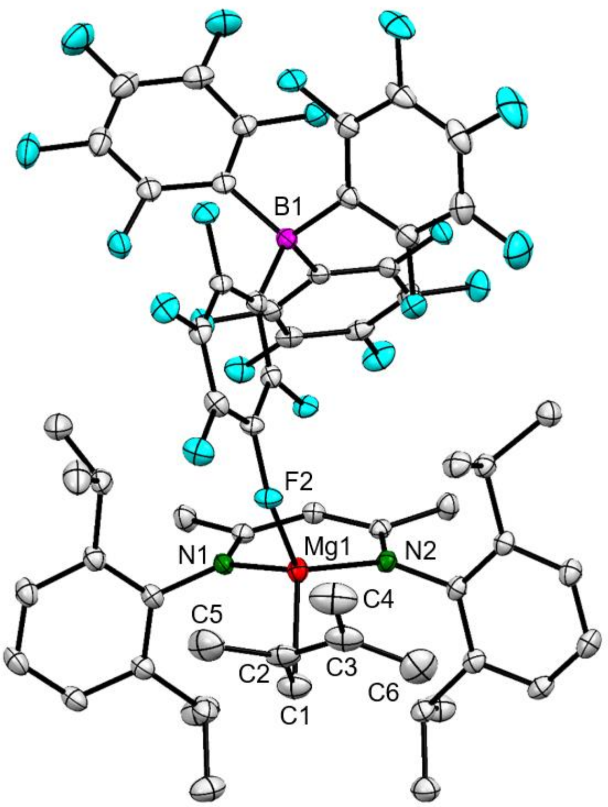

Figure S53: ORTEP representation of [(^{Me}BDI)Mg$^+$(*dmbd*)][B(C_6F_5)$_4^-$] (**6**) with coordinated counter anion (probability level 50%). Hydrogen atoms were omitted for clarity.

1.2.7 Structure determination of [(^{Me}BDI)Mg$^+$(*eb*)][B(C_6F_5)$_4^-$] (7)

A colorless crystal of compound [(^{Me}BDI)Mg$^+$(*eb*)][B(C_6F_5)$_4^-$] was embedded in inert perfluoropolyalkylether (viscosity 1800 cSt; ABCR GmbH) and mounted using a Hampton Research CryoLoop. The crystal was then flash cooled to 100.0(1) K in a nitrogen gas stream and kept at this temperature during the experiment. The crystal structure was measured on a SuperNova diffractometer with Atlas S2 detector using a CuKα microfocus source. The measured data was processed with the CrysAlisPro (v40.18b) software package.[S2] Using Olex2,[S3] the structure was solved with the ShelXT[S4] structure solution program using Intrinsic Phasing and refined with the ShelXL[S5] refinement package using Least Squares minimization.

All non-hydrogen atoms were refined anisotropically. Most hydrogen atoms were placed in ideal positions and refined as riding atoms with relative isotropic displacement parameters. The position of the hydrogen atom at C9 was observed from difference Fourier maps and refined.

Disorder of the *eb* ligand was observed, and was modeled with the help of similarity restraints (SIMU) and rigid bond restraints (RIGU).[S6] The relative occupancies of the two alternative orientations were refined to 0.531(4) and 0.469(4), respectively.

Additionally, voids with heavily disordered solvent (mixture of chlorobenzene/methylcyclohexane/*n*-hexane) were found within the crystal. A suitable disorder model for these solvent molecules could not be built. Therefore, their contribution to the structure factors was secured by back-Fourier transformation using the solvent mask routine[S7, S8] of the program Olex2.[S3] The solvent accessible voids treated this way had a size of 784.6 Å^3 (12.8% of the unit cell) and contained 240.2 electrons/unit cell. Crystallographic and refinement data are summarized in Table S1 (see below).

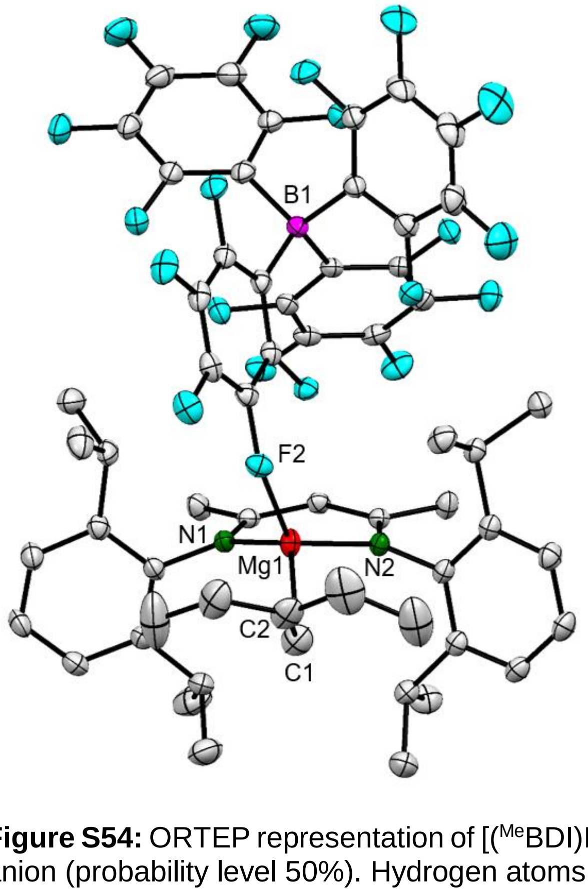

Figure S54: ORTEP representation of [(^{Me}BDI)Mg^+(*eb*)][$B(C_6F_5)_4^-$] (**7**) with coordinated counter anion (probability level 50%). Hydrogen atoms were omitted for clarity.

Table S1. Crystal data and structure refinement.

**Identification code**	**[(^{tBu}BDI)Mg$^+$ (*divinylsiloxane*)][B(C_6F_5)$_4^-$] (1)**	**[(^{tBu}BDI)Mg$^+$ (*cod*)] [B(C_6F_5)$_4^-$] (2)**	**[(^{tBu}BDI)Mg$^+$ (*coe*)] [B(C_6F_5)$_4^-$] (3)**	**[(^{Me}BDI)Mg$^+$ (*cod*)] [B(C_6F_5)$_4^-$] (4)**
**Empirical formula**	$C_{67}H_{71}BF_{20}MgN_2OSi_2$	$C_{67}H_{65}BF_{20}MgN_2$	$C_{67}H_{67}BF_{20}MgN_2$	$C_{61}H_{53}BF_{20}MgN_2$
**Formula weight**	1391.55	1313.33	1315.34	1229.17
**Temperature/K**	153.0(1)	100.0(2)	100.0(2)	100.0(1)
**Crystal system**	monoclinic	orthorhombic	orthorhombic	triclinic
**Space group**	$P2_1/c$	Pbca	Pbca	P-1
**a/Å**	19.32298(12)	17.91200(13)	18.1036(4)	12.38540(10)
**b/Å**	15.97451(10)	18.52420(16)	18.6226(3)	32.0109(4)
**c/Å**	21.60650(14)	37.0515(3)	36.7413(7)	37.9120(5)
**α/°**	90	90	90	108.2360(10)
**β/°**	93.8808(6)	90	90	94.8140(10)
**γ/°**	90	90	90	95.6630(10)
**Volume/Å^3**	6654.09(7)	12293.90(17)	12386.8(4)	14101.1(3)
**Z**	4	8	8	10
**ρ_{calc}g/cm^3**	1.389	1.419	1.411	1.447
**μ/mm^{-1}**	1.459	1.173	1.164	1.239
**F(000)**	2880.0	5424.0	5440.0	6300.0
**Crystal size/mm^3**	0.273 × 0.235 × 0.184	0.436 × 0.211 × 0.145	0.516 × 0.128 × 0.09	0.373 × 0.329 × 0.277
**Crystal color**	colorless	colorless	colorless	colorless
**Radiation**	CuKα (λ = 1.54184)	Cu Kα (λ = 1.54184)	Cu Kα (λ = 1.54184)	CuKα (λ = 1.54184)
**2Θ range for data collection/°**	6.888 to 129.232	6.864 to 144.91	6.856 to 145.456	6.516 to 147.662
**Index ranges**	-17 ≤ h ≤ 22, -18 ≤ k ≤ 18, -18 ≤ l ≤ 25	-21 ≤ h ≤ 21, -22 ≤ k ≤ 22, -38 ≤ l ≤ 45	-9 ≤ h ≤ 22, -22 ≤ k ≤ 14, -45 ≤ l ≤ 41	-15 ≤ h ≤ 15, -39 ≤ k ≤ 35, -46 ≤ l ≤ 47
**Reflections collected**	37482	43671	28222	207204
**Independent reflections**	11035 [R_{int} = 0.0297, R_{sigma} = 0.0273]	11907 [Rint = 0.0292, Rsigma = 0.0261]	11976 [R_{int} = 0.0300, R_{sigma} = 0.0351]	55818 [R_{int} = 0.0345, R_{sigma} = 0.0280]
**Data/restraints/parameters**	11035/0/877	11907/13/863	11976/176/962	55818/1926/4098
**Goodness-of-fit on F^2**	1.033	1.041	1.041	1.034
**Final R indexes [I>=2σ (I)]**	R_1 = 0.0332, wR_2 = 0.0818	R1 = 0.0405, wR2 = 0.0944	R_1 = 0.0441, wR_2 = 0.1038	R_1 = 0.0631, wR_2 = 0.1692
**Final R indexes [all data]**	R_1 = 0.0432, wR_2 = 0.0883	R1 = 0.0467, wR2 = 0.0979	R_1 = 0.0602, wR_2 = 0.1122	R_1 = 0.0730, wR_2 = 0.1794
**Largest diff. peak/hole / e Å^{-3}**	0.23/-0.31	0.86/-0.24	0.30/-0.26	0.89/-0.49

Table S1. Crystal data and structure refinement (continued).

Identification code	[(^{Me}BDI)Mg$^+$(*cht*)] [B(C_6F_5)$_4^-$] (5)	[(^{Me}BDI)Mg$^+$(*dmbd*)] [B(C_6F_5)$_4^-$] (6)	[(^{Me}BDI)Mg$^+$(*eb*)] [B(C_6F_5)$_4^-$] (7)
**Empirical formula**	$C_{60}H_{49}BF_{20}MgN_2$	$C_{59}H_{51}BF_{20}MgN_2$ [+ solvent]	$C_{59}H_{53}BF_{20}MgN_2$
**Formula weight**	1213.13	1203.14	1205.15
**Temperature/K**	100.0(1)	100.0(1)	100.0(1)
**Crystal system**	monoclinic	monoclinic	monoclinic
**Space group**	$P2_1/n$	$P2_1/n$	$P2_1/n$
**a/Å**	19.4088(4)	19.54490(15)	19.3650(2)
**b/Å**	14.2087(3)	14.04613(11)	14.14906(12)
**c/Å**	22.7836(8)	23.28419(18)	23.2776(2)
**α/°**	90	90	90
**β/°**	104.775(3)	106.3373(8)	105.8893(11)
**γ/°**	90	90	90
**Volume/Å^3**	6075.3(3)	6134.11(9)	6134.28(11)
**Z**	4	4	4
**ρ_{calc}g/cm^3**	1.326	1.303	1.305
**μ/mm^{-1}**	1.145	1.128	1.128
**F(000)**	2480.0	2464.0	2472.0
**Crystal size/mm^3**	0.23 × 0.186 × 0.154	0.268 × 0.229 × 0.161	0.309 × 0.233 × 0.175
**Crystal color**	colorless	colorless	colorless
**Radiation**	CuKα (λ = 1.54184)	CuKα (λ = 1.54184)	CuKα (λ = 1.54184)
**2Θ range for data collection/°**	6.922 to 145.984	6.954 to 145.394	6.956 to 145.284
**Index ranges**	$-23 \le h \le 19$, $-12 \le k \le 17$, $-21 \le l \le 27$	$-24 \le h \le 24$, $-17 \le k \le 12$, $-28 \le l \le 28$	$-16 \le h \le 23$, $-17 \le k \le 11$, $-28 \le l \le 27$
**Reflections collected**	23204	46754	21358
**Independent reflections**	11675 [R_{int} = 0.0246, R_{sigma} = 0.0337]	11995 [R_{int} = 0.0216, R_{sigma} = 0.0159]	11828 [R_{int} = 0.0230, R_{sigma} = 0.0336]
**Data/restraints/parameters**	11675/16/799	11995/0/772	11828/246/813
**Goodness-of-fit on F^2**	1.050	1.032	1.030
**Final R indexes [I>=2σ (I)]**	R_1 = 0.0406, wR_2 = 0.1068	R_1 = 0.0313, wR_2 = 0.0806	R_1 = 0.0371, wR_2 = 0.0925
**Final R indexes [all data]**	R_1 = 0.0513, wR_2 = 0.1136	R_1 = 0.0338, wR_2 = 0.0826	R_1 = 0.0452, wR_2 = 0.0980
**Largest diff. peak/hole / e Å^{-3}**	0.73/-0.28	0.26/-0.23	0.25/-0.31

2. Computational Details

General

All calculations were carried out using Gaussian 16A. [S9] All methods were used as implemented. All structures were fully optimized on a ωB97XD/6-31+G** level of theory. [S10-S12] Frequencies were calculated at ωB97XD/6-31G* level of theory as every larger basis set lead to convergency problems. Energies were obtained on a ωB97XD/6-311+G**//6-31+G** level of theory. Topological analyses were carried out using AIMAll V17 with the wavefunctions obtained from the ωB97XD/6-311+G**//6-31+G** calculations. [S13, S14]

Table S2. Coordination Energies.

$[(^{Me}BDI)Mg^+][B(C_6F_5)_4^-]$ + alkene → $(^{Me}BDI)Mg^+(\textit{alkene})][B(C_6F_5)_4^-]$			
Alkene	ΔE [kcal/mol]	ΔH [kcal/mol]	ΔG (298 K, 1 atm) [kcal/mol]
C_6H_6	-6.80	-5.93	+5.55
cod	-10.78	-5.94	+7.41
cht	-12.70	-7.84	-0.24
dmbd	-11.22	-6.96	+4.23
eb	-14.43	-10.23	-0.21
$[(^{tBu}BDI)Mg^+][B(C_6F_5)_4^-]$ + alkene → $[(^{tBu}BDI)Mg^+(\textit{alkene})][B(C_6F_5)_4^-]$			
Alkene	ΔE [kcal/mol]	ΔH [kcal/mol]	ΔG (298 K, 1 atm) [kcal/mol]
divinylsiloxane	-39.81	-37.12	-18.03
cod	-22.37	-22.12	-7.06
coe	-24.04	-24.31	-11.37

Table S3. NPA Charges of Mg alkene complexes as their $B(C_6F_5)_4^-$ salts.

Alkene	(^{Me}BDI) System	(^{tBu}BDI) System
cod<br>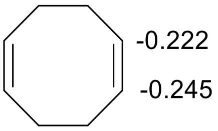	[(^{Me}BDI)Mg^+(*cod*)] (**4**)<br>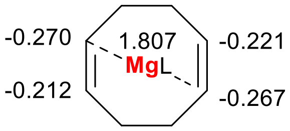<br>*cod* = 0.054<br>L = -0.923<br>$B(C_6F_5)_4$ = -0.921	[(^{tBu}BDI)Mg^+(*cod*)] (**2**)<br>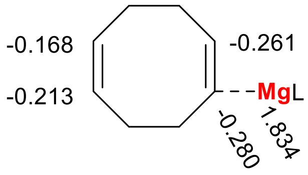<br>*cod* = 0.026<br>L = -0.909<br>$B(C_6F_5)_4$ = -0.926
cht<br>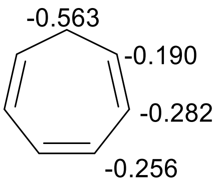	[(^{Me}BDI)Mg^+(*cht*)] (**5**)<br>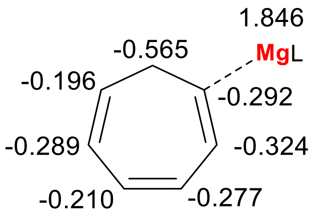<br>*cht* = 0.034<br>L = -0.939<br>$B(C_6F_5)_4$ = -0.941	
dmbd<br>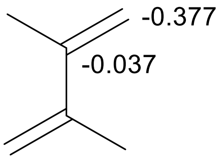	[(^{Me}BDI)Mg^+(*dmbd*)] (**6**)<br>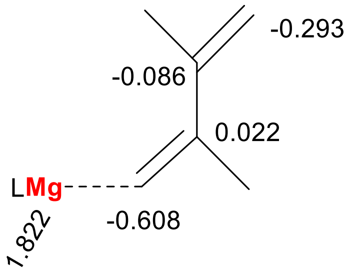<br>*dmbd* = 0.016<br>L = -0.917<br>$B(C_6F_5)_4$ = -0.895	

eb<br>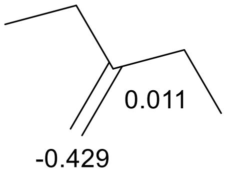 	[(^{Me}BDI)Mg$^+$(*eb*)] (**7**)<br>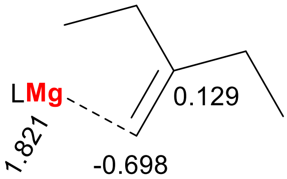<br><br>*eb* = 0.023<br>L = -0.928<br>$B(C_6F_5)_4$ = -0.890	
Divinylsiloxane<br>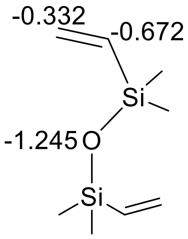 		[(^{tBu}BDI)Mg$^+$ (*divinylsiloxane*)] (**1**)<br>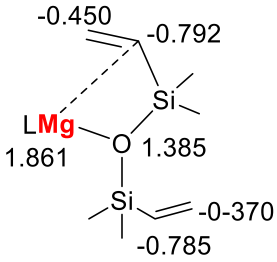<br><br>*divinylsiloxane* = 0.032<br>L = -0.928<br>$B(C_6F_5)_4$ = -0.964
coe<br>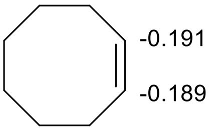 		[(^{tBu}BDI)Mg$^+$(*coe*)] (**3**)<br>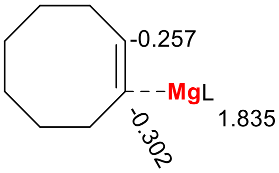<br><br>*coe* = 0.016<br>L = -0.888<br>$B(C_6F_5)_4$ = -0.950

Table S4. QTAIM analysis of free alkenes and of Mg alkene complexes as their $B(C_6F_5)_4^-$ salts.

Bond distance [Å]	Electron density [a.u.]	Bond ellipticity [a.u.]	Delocalization index [a.u.]
cod 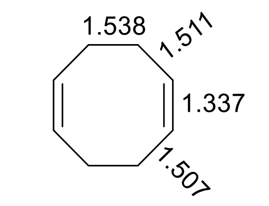	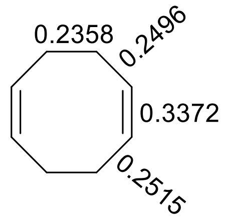	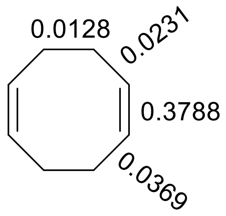	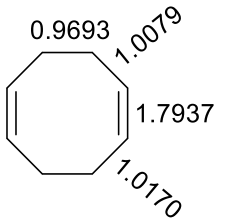
[(^{Me}BDI)Mg$^+$(*cod*)] (**4**) 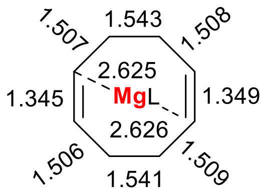	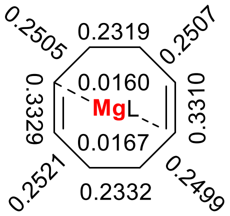	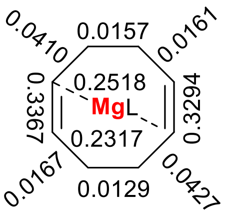	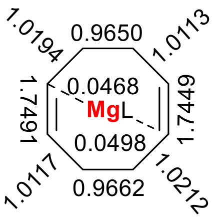
cht 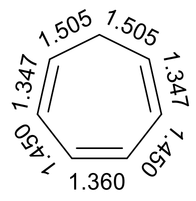	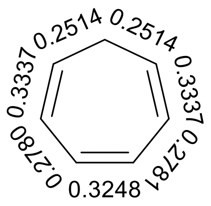	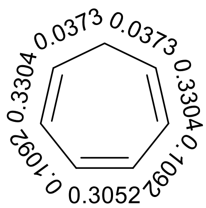	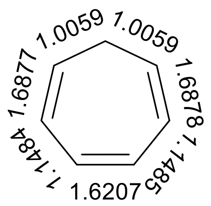
[(^{Me}BDI)Mg$^+$(*cht*)] (**5**) 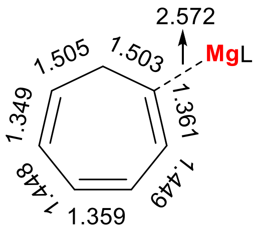	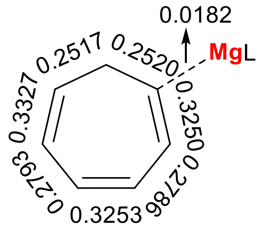	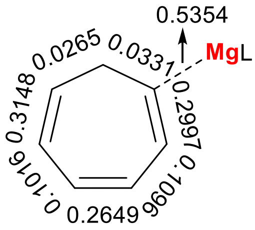	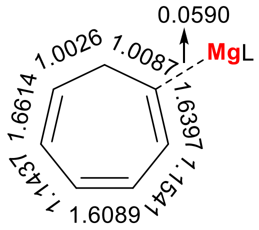
dmbd 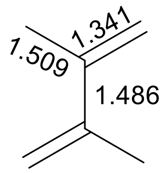	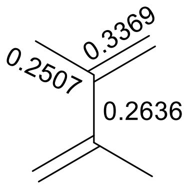	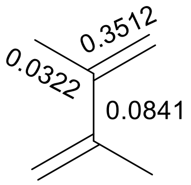	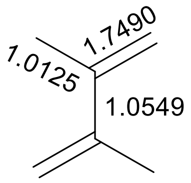

[(^{Me}BDI)Mg^{+}(*dmbd*)] (**6**) 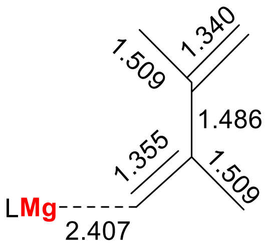	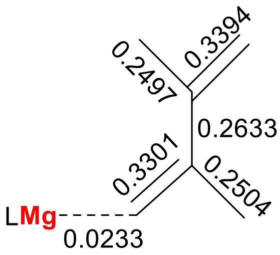	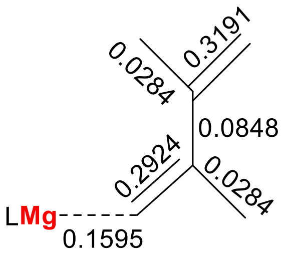	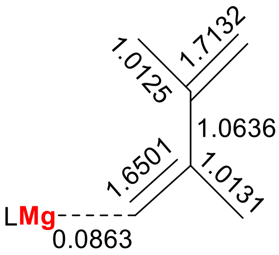
eb 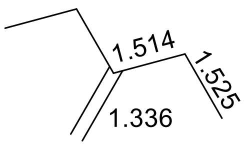	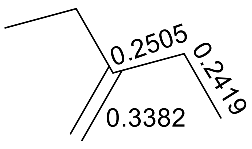	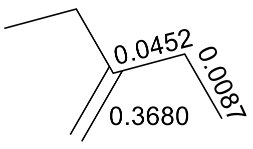	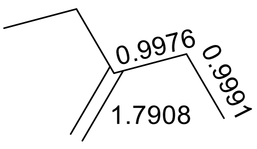
[(^{Me}BDI)Mg^{+}(*eb*)] (**7**) 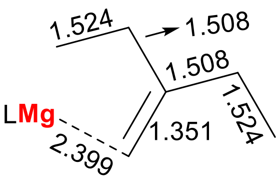	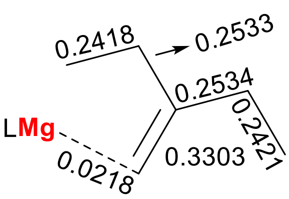	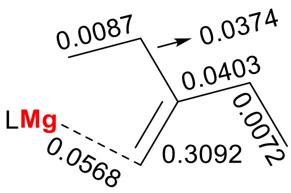	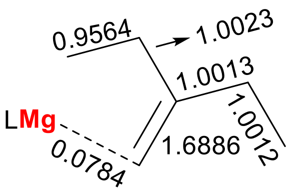
divinylsiloxane 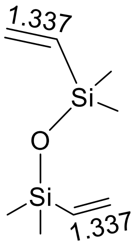	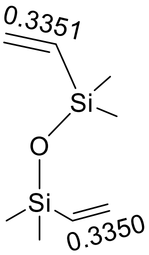	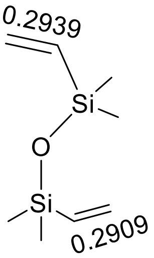	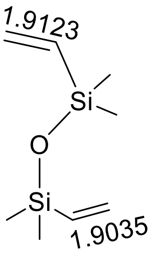
[(^{tBu}BDI)Mg^{+} (*divinylsiloxane*)] (**1**) 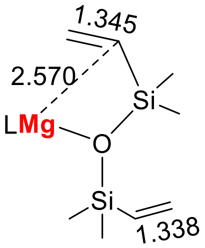	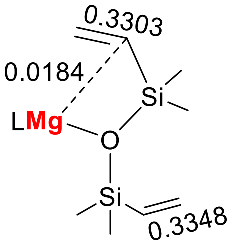	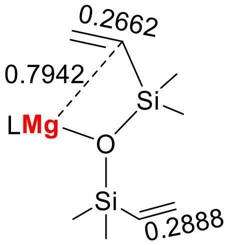	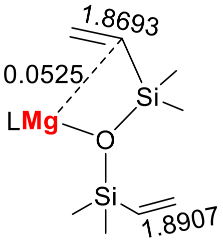

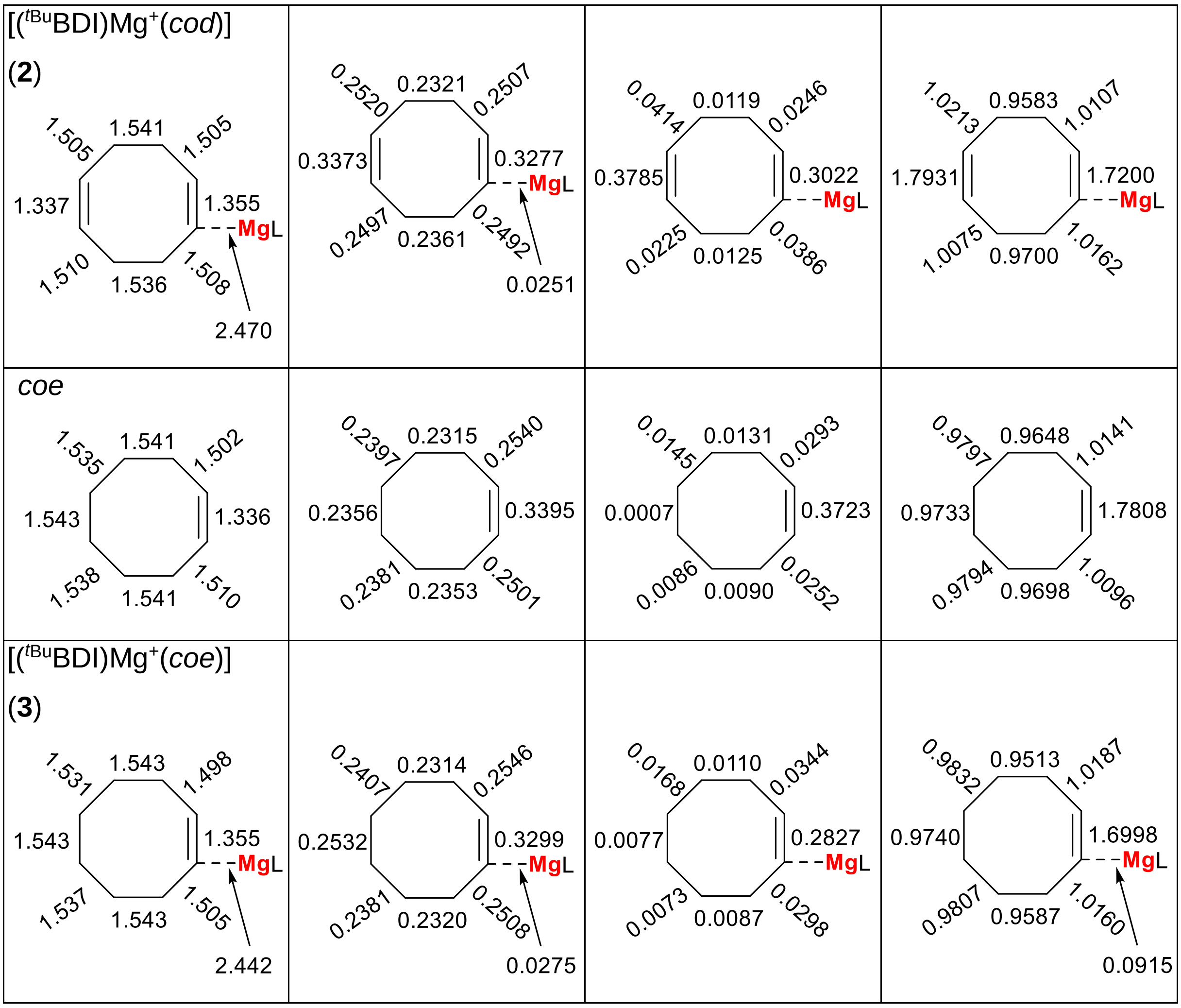

[(^{Me}BDI)Mg$^+$(*cod*)][B(C_6F_5)$_4^-$] (4)

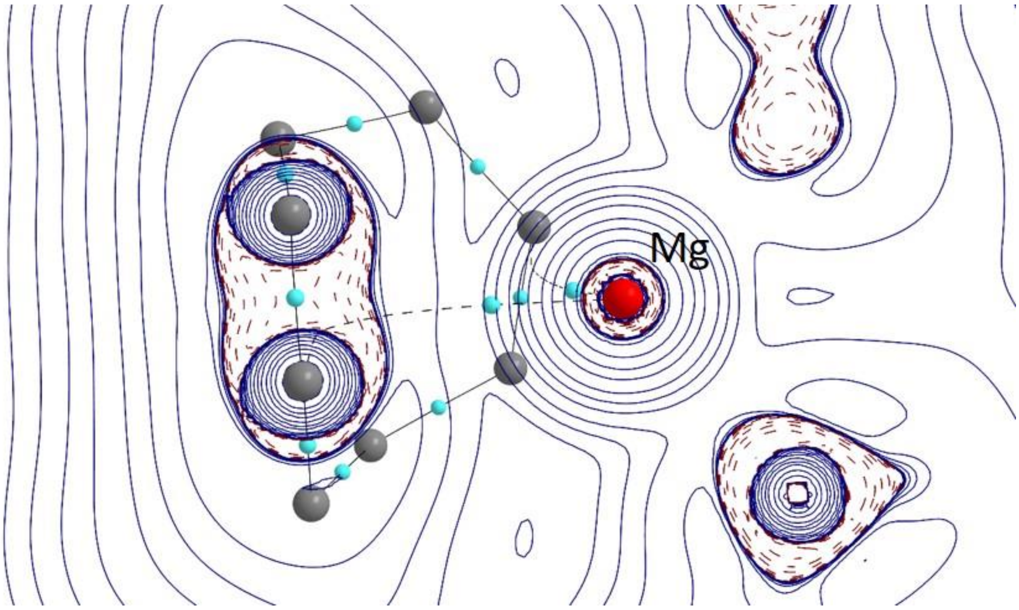

Figure S55: Laplacian of the electron density in the plane of one double bond and Mg.

[(^{Me}BDI)Mg$^+$(*cht*)][B(C_6F_5)$_4^-$] (5)

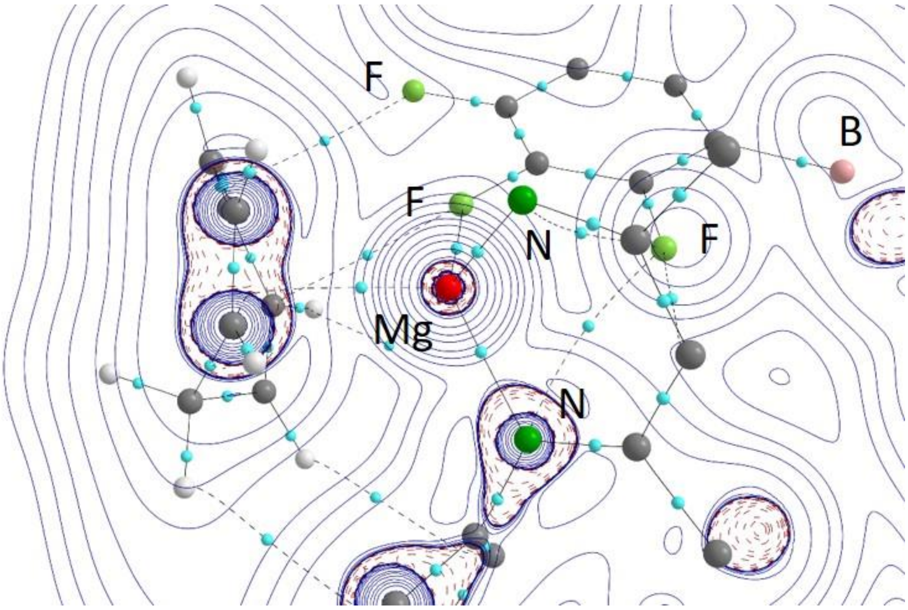

Figure S56: Laplacian of the electron density in the plane of the coordinated double bond and Mg.

[(^{Me}BDI)Mg$^+$(*dmbd*)][B(C_6F_5)$_4^-$] (6)

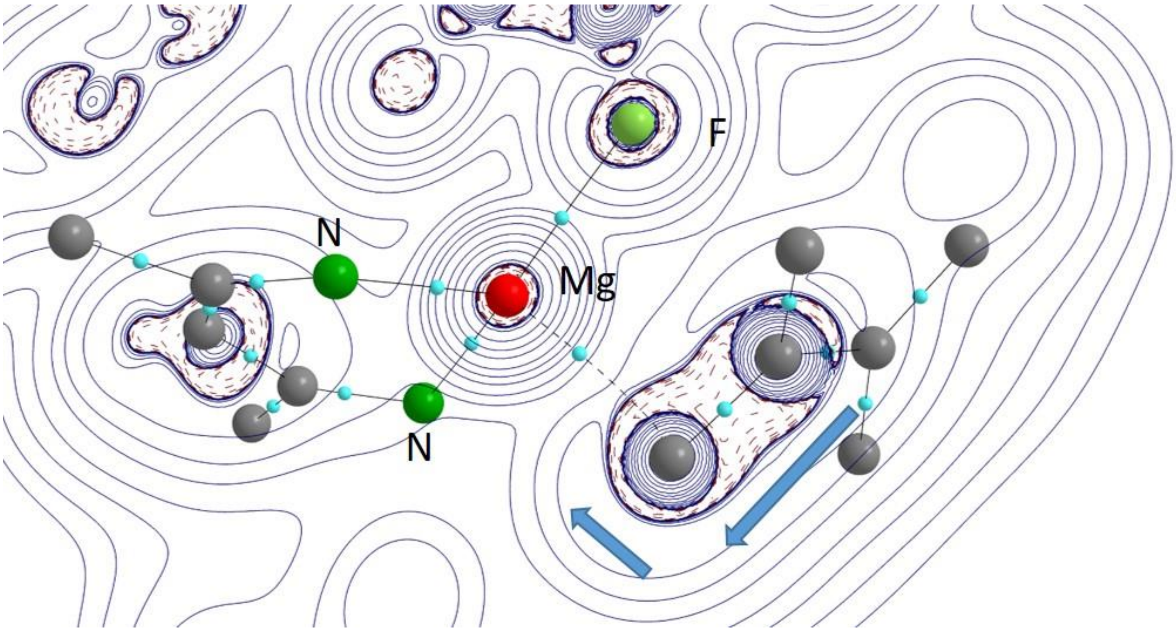

Figure S57: Laplacian of the electron density in the plane of the double bond coordinated to Mg, blue arrows show horizontal and vertical polarization of the electron density in the C=C bond.

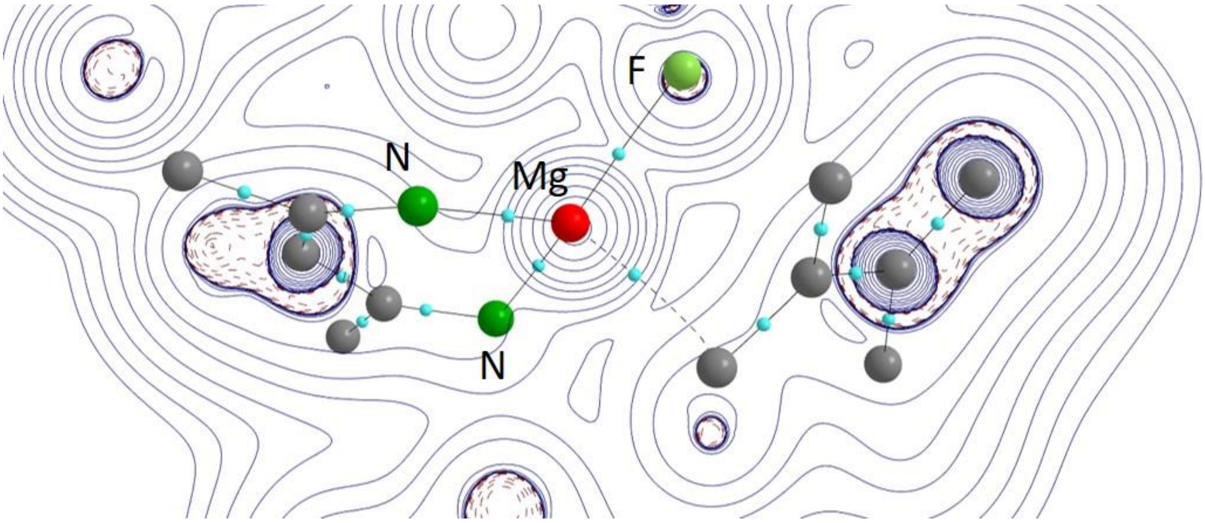

Figure S58: Laplacian of the electron density in the plane of the double bond <u>not</u> coordinated to Mg.

[(^{tBu}BDI)Mg^{+}(*divinylsiloxane*)][B(C_6F_5)$_4^{-}$] (1)

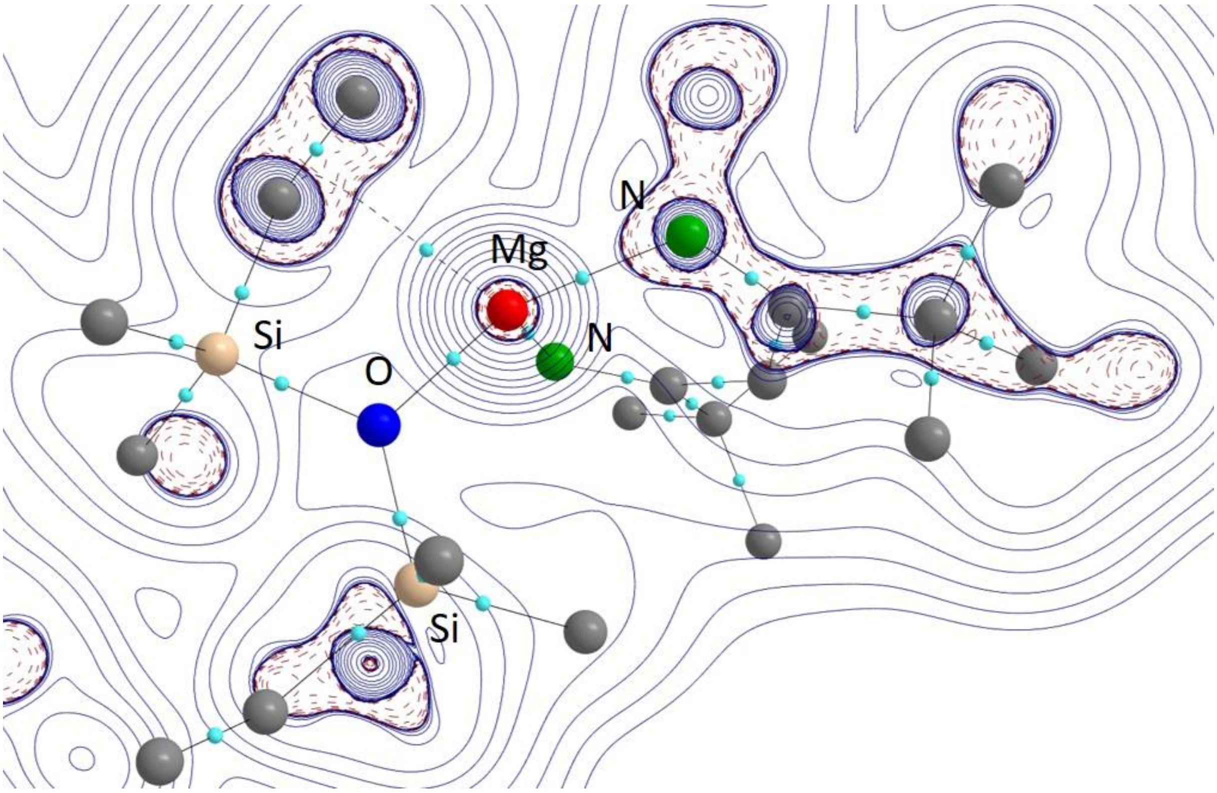

Figure S59: Laplacian of the electron density in the plane of Mg and the coordinating double bond.

[(^{tBu}BDI)Mg^{+}(*cod*)][B(C_6F_5)$_4^{-}$] (2)

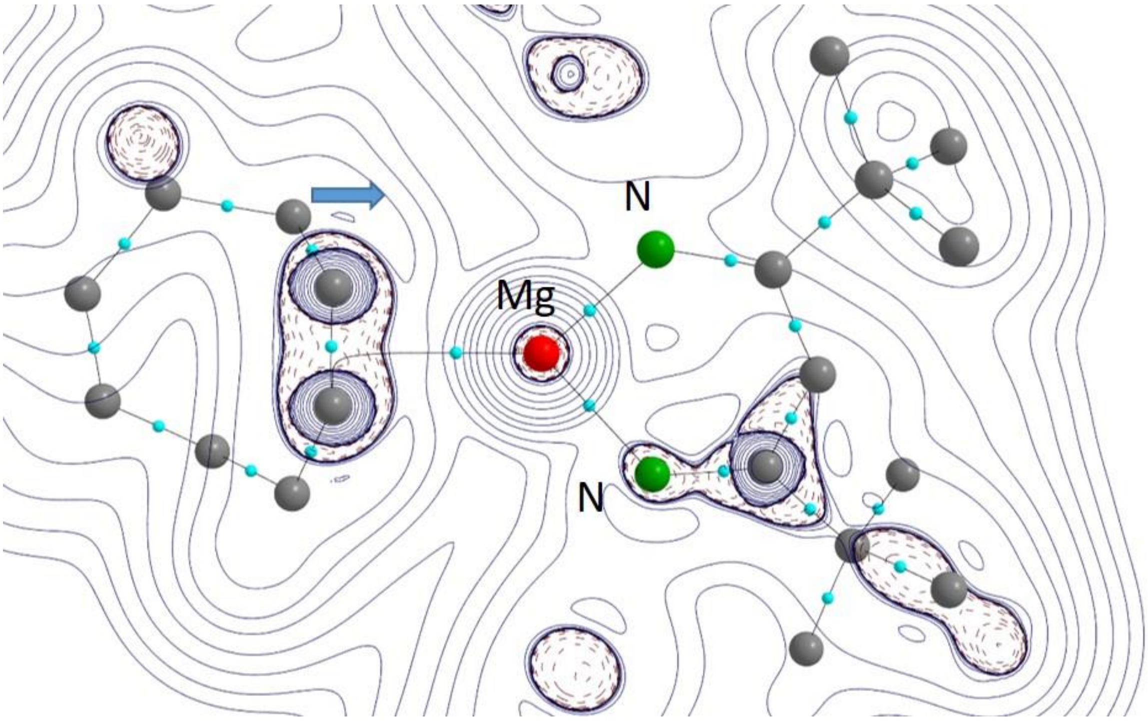

Figure S60: Laplacian of the electron density in the plane of Mg and the coordinating double bond; blue arrow shows vertical polarization of the electron density in the C=C bond.

[(^{tBu}BDI)Mg^{+}(*coe*)][B(C_6F_5)$_4^{-}$] (3)

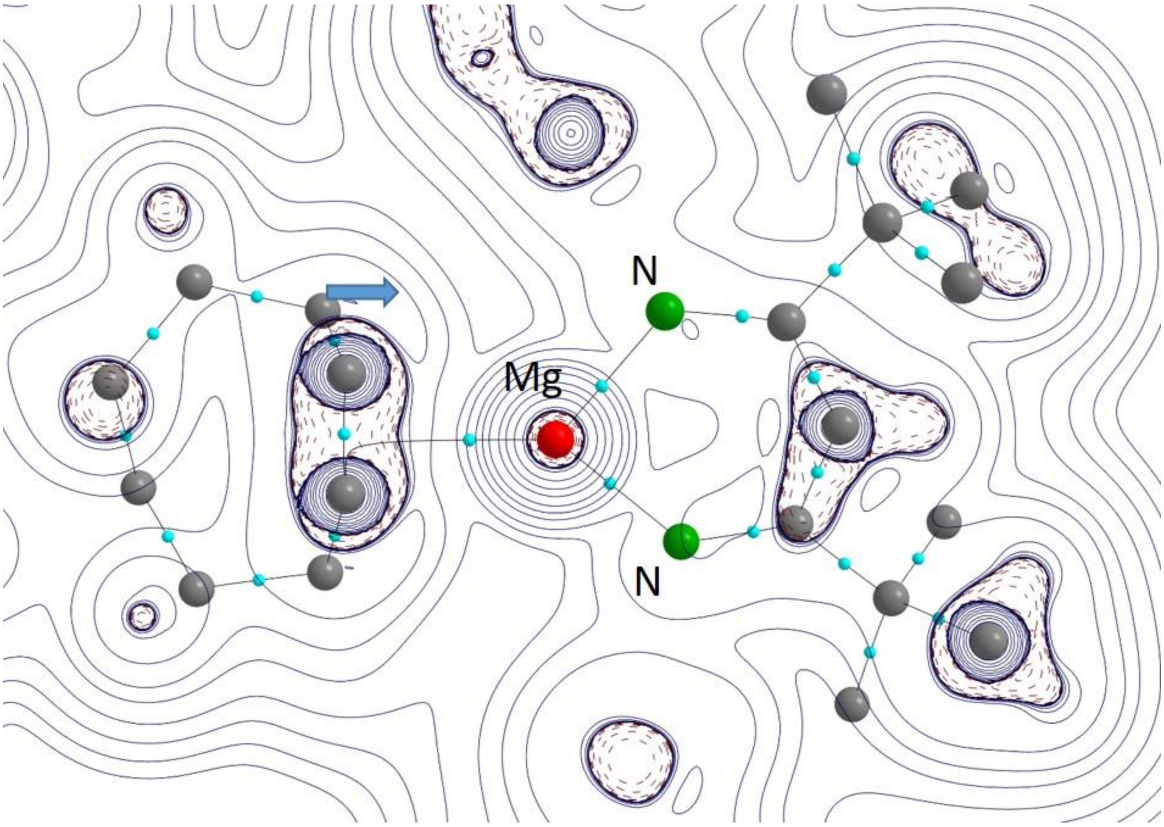

Figure S61: Laplacian of the electron density in the plane of Mg and the coordinating double bond; blue arrow shows vertical polarization of the electron density in the C=C bond.

XYZ Coordinates

136
[(^{tBu}BDI)Mg$^+$][B(C_6F_5)$_4^-$]

```
C    5.014780   3.169153   0.494555
C    5.660219  -1.520493   1.479188
C    6.105231   3.149246  -0.606639
C    7.092179  -0.940604   1.543864
C    4.154803   1.869788   0.319866
C    4.676902  -0.688044   0.585959
N    2.892922   1.809556  -0.115161
N    3.635796  -1.211165  -0.072263
Mg   2.090311   0.010314  -0.513430
C    4.889028   0.705135   0.660917
C    1.938573   2.887110  -0.117695
C    3.558181  -2.538280  -0.613495
H    5.830017   0.944885   1.116505
C    5.762548   3.160024   1.856924
C    5.062199  -1.409466   2.903255
C    4.274580   4.510746   0.366665
C    5.809130  -3.012066   1.132843
C    1.298308   3.254567   1.088354
C    2.672478  -3.510568  -0.093051
C    0.390124   4.320081   1.061307
C    2.580451  -4.746400  -0.747210
C    0.085551   4.997049  -0.104306
C    3.326120  -5.045308  -1.871396
C    1.535769   3.485270  -1.338156
C    4.265673  -2.800719  -1.818822
C    0.638671   4.552188  -1.297207
C    4.153275  -4.064655  -2.399310
C    1.463717   2.616986   2.470999
C    1.802343  -3.438203   1.170105
H   -0.096569   4.609076   1.989232
H    1.900532  -5.494315  -0.346379
H   -0.612302   5.827628  -0.096848
H    3.238292  -6.013701  -2.352363
C    1.961879   3.032148  -2.732102
C    5.127365  -1.804210  -2.606864
H    0.348025   5.029733  -2.228116
H    4.707598  -4.273403  -3.310190
H    0.468740   2.706608   2.916325
H    0.880168  -3.968287   0.899116
C    2.402572   3.414524   3.383294
C    2.425008  -4.235880   2.329841
C    1.772285   1.122625   2.517182
C    1.355373  -2.070235   1.667015
H    1.608084   0.748366   3.531748
H    0.686553  -2.187487   2.524003
H    2.798917   0.873383   2.240413
H    2.187581  -1.444224   1.983331
H    1.066977   0.580161   1.877136
H    0.752404  -1.545251   0.916747
H    2.369403   3.010828   4.400560
H    1.707320  -4.323718   3.151745
H    2.107476   4.467282   3.426500
H    2.704253  -5.244021   2.012278
H    3.434771   3.372007   3.037765
H    3.322025  -3.749239   2.720521
H    1.419497   3.691264  -3.417829
H    5.285197  -2.301631  -3.570363
C    1.484996   1.613534  -3.053574
C    4.446762  -0.475359  -2.946131
C    3.448077   3.178631  -3.060740
C    6.525983  -1.547053  -2.037611
H    1.521374   1.421698  -4.130447
H    5.034095   0.060231  -3.699336
H    0.461447   1.435982  -2.718232
H    3.452662  -0.642233  -3.368711
H    2.173689   0.879753  -2.622125
H    4.378502   0.182109  -2.074375
H    3.618914   2.962617  -4.120779
H    7.128129  -1.006329  -2.775620
H    4.046842   2.476807  -2.478020
H    6.478338  -0.927158  -1.141818
H    3.805168   4.193079  -2.862104
H    7.045517  -2.479102  -1.798149
H    4.873684  -3.562954   1.184034
H    6.234194  -3.166466   0.140303
H    6.491192  -3.462600   1.860997
H    7.719815  -1.633654   2.111631
H    7.544018  -0.823731   0.556069
H    7.144163   0.018918   2.063769
H    4.041073  -1.794395   2.945358
H    5.669340  -1.992544   3.603961
H    5.044419  -0.369888   3.242614
H    3.548285   4.676736   1.162803
H    5.019891   5.310715   0.425681
H    3.758637   4.616048  -0.586909
H    6.806984   3.969451  -0.420632
H    6.668031   2.212144  -0.603520
H    5.684414   3.294151  -1.602284
H    5.167838   2.736206   2.667443
H    6.696627   2.592925   1.807183
H    6.028001   4.185089   2.131186
B   -2.952554  -0.288937   0.025105
C   -4.574918  -0.576429  -0.081732
C   -2.843932   1.304736  -0.419968
C   -2.335717  -0.634467   1.523585
C   -2.057910  -1.278669  -0.969090
C   -5.200852  -0.468689  -1.323880
C   -6.565540  -0.593071  -1.528240
C   -7.392770  -0.827299  -0.438377
C   -5.451367  -0.793770   0.977523
C   -6.828625  -0.925886   0.822658
F   -4.454728  -0.241329  -2.423941
F   -7.088208  -0.491487  -2.753931
F   -8.709985  -0.950825  -0.603810
F   -5.010849  -0.878625   2.244879
F   -7.611321  -1.138171   1.884326
C   -2.441654  -1.938212   2.013131
C   -2.010123  -2.343870   3.266763
C   -1.427992  -1.413684   4.116636
C   -1.694535   0.239997   2.394188
C   -1.272928  -0.111546   3.673473
F   -3.025727  -2.885472   1.259324
F   -2.148236  -3.612237   3.663099
F   -1.002888  -1.773580   5.328454
F   -1.437666   1.512171   2.040321
F   -0.708178   0.799096   4.476437
C   -2.493417  -2.433025  -1.628978
C   -1.682379  -3.230152  -2.437849
C   -0.345467  -2.909831  -2.629256
C   -0.712654  -1.051932  -1.167102
C    0.125028  -1.793471  -1.968578
F   -3.751350  -2.856422  -1.517847
F   -2.183557  -4.307662  -3.034661
F    0.443614  -3.636105  -3.410081
F   -0.059106   0.026535  -0.557106
F    1.420937  -1.384806  -2.093322
C   -2.338319   1.808608  -1.613708
C   -2.384080   3.148791  -1.979509
C   -2.976167   4.068043  -1.133757
C   -3.459946   2.273193   0.376610
C   -3.523225   3.621759   0.060792
F   -1.768201   0.998507  -2.535235
F   -1.858899   3.549764  -3.144395
F   -3.015902   5.362316  -1.458213
F   -4.045268   1.910124   1.529399
F   -4.101383   4.493580   0.891508
```

165
[(^{tBu}BDI)Mg$^+$(di*vinylsiloxane*)][B(C_6F_5)$_4^-$] (**1**)

```
Si  -0.946501   1.366131  -0.862553
Si  -2.823656   1.201648  -3.346699
Mg  -3.644668   0.217166  -0.208250
O   -2.465199   1.044875  -1.654938
N   -3.836908  -1.799745   0.049319
N   -5.553229   0.717937   0.140547
C   -1.495848   1.057232   0.923922
H   -0.947705   0.273931   1.451044
C   -2.471791   1.685695   1.604058
H   -3.058241   2.503610   1.184641
H   -2.711221   1.429694   2.634541
C    0.286116   0.070201  -1.299490
H   -0.038584  -0.925361  -0.982227
H    1.220305   0.287959  -0.777460
H    0.494936   0.056136  -2.371090
C   -0.426469   3.108796  -1.227347
H   -0.125103   3.194967  -2.276666
H    0.449239   3.364009  -0.623608
H   -1.214472   3.838699  -1.026594
C   -1.276601   0.844522  -4.325706
H   -0.905183  -0.174714  -4.414668
C   -0.577036   1.799946  -4.949592
H   -0.866644   2.848689  -4.921110
H    0.324322   1.571439  -5.513056
C   -3.373902   2.942201  -3.728224
H   -4.354342   3.158677  -3.299571
H   -3.455623   3.053526  -4.814969
H   -2.663447   3.690991  -3.366262
C   -4.214721  -0.017462  -3.587586
H   -3.891543  -1.054441  -3.467908
H   -4.651556   0.087348  -4.586162
H   -5.017640   0.169859  -2.865394
C   -5.024729  -2.331403  -0.161638
C   -6.137787  -1.510786  -0.560583
H   -6.940132  -2.089266  -0.977705
C   -6.459751  -0.173056  -0.344118
C   -7.871688   0.276115  -0.860418
C   -8.579596   1.378100  -0.045013
H   -8.768043   1.077537   0.987042
H   -9.548350   1.575483  -0.515335
H   -8.034077   2.317683  -0.020558
C   -7.627724   0.814998  -2.288107
H   -8.558696   1.220183  -2.699097
H   -7.282498   0.017464  -2.953390
H   -6.881664   1.614083  -2.291053
C   -8.880573  -0.886739  -0.968125
H   -8.612021  -1.619869  -1.733451
H   -9.855621  -0.482158  -1.254622
H   -9.007876  -1.410518  -0.015721
C   -5.413536  -3.835678  -0.013407
C   -4.334521  -4.773685   0.552737
H   -3.480818  -4.890546  -0.109238
H   -4.784973  -5.762660   0.683617
H   -3.957835  -4.454244   1.522794
C   -5.833618  -4.400608  -1.389460
H   -6.686124  -3.874051  -1.824730
H   -6.122926  -5.449197  -1.266850
H   -5.010022  -4.367556  -2.107046
C   -6.624999  -3.915465   0.947740
H   -6.400181  -3.448601   1.911242
H   -6.870843  -4.966008   1.131060
H   -7.514732  -3.427140   0.546374
C   -2.598486  -2.452880   0.378376
C   -2.065487  -2.291876   1.677423
C   -0.776373  -2.764689   1.930686
H   -0.342944  -2.637686   2.916110
C   -0.023997  -3.386807   0.945207
H    0.979846  -3.734809   1.160620
C   -0.565091  -3.550386  -0.320783
H    0.027026  -4.038283  -1.088680
C   -1.844073  -3.086414  -0.632720
C   -2.344733  -3.262859  -2.060162
H   -3.396608  -2.965125  -2.093754
C   -2.245320  -4.707735  -2.570692
H   -1.202570  -5.026591  -2.658790
H   -2.693842  -4.779560  -3.567077
H   -2.758293  -5.419966  -1.920071
C   -1.573985  -2.336043  -3.002845
H   -1.644668  -1.304955  -2.657889
H   -1.969544  -2.395144  -4.022801
H   -0.512535  -2.598885  -3.036503
C   -2.872237  -1.691666   2.823974
H   -3.560386  -0.945547   2.407679
C   -3.725824  -2.774646   3.506457
H   -4.597846  -3.043310   2.907300
H   -4.081925  -2.425763   4.478559
H   -3.131118  -3.677995   3.676775
C   -2.014223  -0.995395   3.886865
H   -1.467136  -1.717920   4.500035
H   -2.659259  -0.430992   4.566851
H   -1.276882  -0.311007   3.463178
C   -5.911799   1.784007   1.018478
C   -6.316108   1.466968   2.337806
C   -6.717303   2.501609   3.183874
H   -7.070225   2.268777   4.183176
C   -6.660028   3.830161   2.786179
H   -6.976040   4.619714   3.460115
C   -6.167252   4.135927   1.527052
H   -6.098232   5.177109   1.225124
C   -5.788424   3.135238   0.629840
C   -5.315128   3.557257  -0.751924
H   -5.050909   2.645471  -1.299660
C   -4.073617   4.456344  -0.670214
H   -4.272330   5.351200  -0.073317
H   -3.765076   4.788305  -1.664713
H   -3.226050   3.940570  -0.212076
C   -6.419589   4.281650  -1.538229
H   -7.337431   3.692434  -1.595029
H   -6.089657   4.497334  -2.560439
H   -6.667608   5.237507  -1.065597
C   -6.302703   0.038966   2.874872
H   -5.639704  -0.548477   2.230826
C   -7.680225  -0.636350   2.841296
H   -8.425225  -0.033996   3.373264
H   -7.631222  -1.618368   3.323692
H   -8.026980  -0.790410   1.818434
C   -5.739684  -0.014511   4.300674
H   -4.763779   0.476150   4.366689
H   -5.628038  -1.053078   4.618442
H   -6.405689   0.467457   5.022474
F    3.847861  -2.480114   0.875852
F    2.616440  -4.236178  -0.700517
F    1.853941  -3.533202  -3.222285
F    2.418993  -1.023322  -4.140331
F    3.701980   0.714393  -2.633635
F    2.614334   2.015788  -0.289292
F    2.738834   4.554513  -1.045193
F    5.160446   5.754002  -1.484786
F    7.446958   4.309750  -1.122613
F    7.356780   1.790759  -0.360831
F    6.480636  -0.276900  -2.052968
F    8.670413  -1.785966  -2.213738
F    9.608936  -3.096003  -0.007562
F    8.274576  -2.855899   2.358929
F    6.094302  -1.381829   2.545528
F    6.152371   1.462582   2.395763
F    5.217721   2.136597   4.793829
F    2.643609   1.531524   5.493901
F    1.044781   0.233897   3.701471
F    1.938298  -0.446414   1.326853
C    3.841327  -0.770382  -0.767838
C    3.519335  -2.067079  -0.359603
C    2.875747  -3.001272  -1.155138
C    2.501668  -2.654536  -2.444582
C    2.802082  -1.387272  -2.906538
C    3.465239  -0.485921  -2.077368
C    4.972804   1.725680  -0.330339
C    3.850185   2.531799  -0.500942
C    3.878287   3.860602  -0.887263
C    5.101594   4.473458  -1.111442
C    6.257476   3.732653  -0.930736
C    6.175498   2.397374  -0.540625
C    6.186011  -0.698503   0.260624
C    6.897217  -0.885136  -0.925496
C    8.035648  -1.666669  -1.042312
C    8.515617  -2.334310   0.075758
C    7.836780  -2.204624   1.275496
C    6.696429  -1.408775   1.343277
C    4.127612   0.433962   1.727211
C    4.885855   1.120797   2.678816
C    4.425674   1.489809   3.934289
C    3.120840   1.184790   4.295516
C    2.320017   0.521804   3.383643
C    2.827724   0.168756   2.138563
B    4.781299   0.174476   0.225847
```

29
divinylsiloxane

```
Si  -1.463358  -0.506260  -0.017706
Si   1.419584   0.638398  -0.041064
O   -0.232927   0.594869  -0.216059
C   -3.043695   0.485980   0.079504
H   -3.991500  -0.042380   0.204261
C   -3.081122   1.820396   0.001939
H   -2.169810   2.401121  -0.124994
H   -4.012588   2.380356   0.059126
C   -1.504243  -1.677199  -1.481764
H   -1.594561  -1.123292  -2.421490
H   -2.353916  -2.365670  -1.411691
H   -0.587770  -2.276031  -1.520996
C   -1.197166  -1.486915   1.561681
H   -0.270356  -2.069184   1.503005
H   -2.021435  -2.188961   1.730192
H   -1.135320  -0.824930   2.431416
C    2.139837  -0.926046  -0.785201
H    1.995867  -1.104734  -1.853382
C    2.814917  -1.852058  -0.095002
H    3.000333  -1.749888   0.973038
H    3.214673  -2.750568  -0.561305
C    1.857749   0.734865   1.779483
H    1.385291   1.613054   2.231634
H    2.939717   0.820336   1.927235
H    1.507260  -0.148461   2.323524
C    2.030803   2.142676  -0.968938
H    1.768596   2.078349  -2.029876
H    3.119347   2.236079  -0.892246
H    1.579940   3.055742  -0.566406
```

156
[(^{tBu}BDI)Mg$^+$(*cod*)][B(C_6F_5)$_4^-$] (**2**)

```
Mg   4.245962   0.293663  -0.300580
```

F -3.288031 0.235043 2.940621
F -1.297878 1.848142 3.584075
F -0.291419 3.628103 1.778185
F -1.344609 3.705398 -0.746364
F -3.281148 2.041357 -1.447404
F -5.381631 3.034686 0.054572
F -7.452361 4.047958 -1.225290
F -9.129607 2.453614 -2.670504
F -8.652497 -0.234134 -2.784901
F -6.571727 -1.288099 -1.500904
F -5.852032 1.423263 2.380420
F -7.406967 0.425057 4.298053
F -7.974440 -2.247159 4.364999
F -6.933317 -3.882504 2.446102
F -5.389688 -2.924468 0.535315
F -4.306092 -0.167660 -2.897941
F -2.706287 -1.841595 -4.167616
F -1.215806 -3.670052 -2.800994
F -1.361358 -3.739319 -0.067545
F -2.874854 -1.994651 1.238631
N 3.323166 -1.415440 -0.710882
N 5.857408 -0.497963 0.567706
C 3.763269 -2.601247 -0.302643
C 5.008926 -2.733019 0.357754
H 5.228870 -3.748345 0.634881
C 5.976443 -1.812597 0.798462
C 2.998331 -3.943877 -0.482444
C 3.900017 -4.904200 -1.291676
H 4.147689 -4.473077 -2.267570
H 3.361023 -5.840571 -1.465620
H 4.835437 -5.148743 -0.783599
C 2.699057 -4.546963 0.907125
H 3.601964 -4.738687 1.491835
H 2.173133 -5.498143 0.780107
H 2.047927 -3.887400 1.489429
C 1.662862 -3.859784 -1.232442
H 0.920872 -3.252937 -0.716957
H 1.247485 -4.868725 -1.306890
H 1.776888 -3.484710 -2.249707
C 7.107813 -2.463940 1.659721
C 8.308908 -1.579136 2.040383
H 8.031358 -0.695659 2.613893
H 8.890408 -1.257017 1.174420
H 8.973352 -2.178595 2.670525
C 7.699521 -3.695107 0.938087
H 8.095683 -3.422970 -0.045753
H 6.982765 -4.505983 0.798262
H 8.526441 -4.095574 1.532420
C 6.439365 -2.903216 2.983358
H 7.186189 -3.360485 3.640693
H 5.638546 -3.628129 2.820813
H 6.012012 -2.040761 3.506112
C 2.007036 -1.033731 -1.127832
C 1.728262 -0.832351 -2.492045
C 0.538222 -0.188303 -2.833979
H 0.299072 -0.019301 -3.879448
C -0.364397 0.225528 -1.860477
H -1.285756 0.720902 -2.146260
C -0.098700 -0.034568 -0.522465
H -0.828389 0.242779 0.229373
C 1.086927 -0.654317 -0.130509
C 2.695267 -1.299615 -3.568926
H 3.379509 -2.018647 -3.106019
C 3.543591 -0.136220 -4.098372
H 2.911183 0.650443 -4.524017
H 4.236997 -0.476511 -4.873889
H 4.147015 0.312572 -3.301824
C 1.983505 -2.011776 -4.726544
H 1.312256 -2.795282 -4.364777
H 2.719705 -2.469459 -5.394610
H 1.386869 -1.316039 -5.324846
C 1.385357 -0.879025 1.345059
H 2.201808 -1.601546 1.421649
C 0.197187 -1.459737 2.116695
H -0.646010 -0.766322 2.164174
H 0.492884 -1.684810 3.146029
H -0.160766 -2.381480 1.652619
C 1.867657 0.419592 2.001488
H 2.742852 0.855441 1.490363
H 2.164706 0.253936 3.041245
H 1.092958 1.191522 1.981642
C 6.837125 0.512462 0.794591
C 7.858121 0.704495 -0.163984
C 8.713584 1.793435 -0.007650
H 9.511040 1.959321 -0.723723
C 8.580047 2.670570 1.066034
H 9.263083 3.507055 1.172425
C 7.575239 2.466197 1.999790
H 7.477827 3.150620 2.837823
C 6.682797 1.397146 1.877054
C 8.036866 -0.283321 -1.312491
H 7.813081 -1.280925 -0.920777
C 5.573251 1.223016 2.902330
H 5.012159 0.319538 2.637983
C 6.119098 1.016957 4.321183
H 6.669058 1.897366 4.669212
H 6.796515 0.159548 4.367471
H 5.297364 0.838831 5.021658
C 4.607501 2.415169 2.858481
H 3.759810 2.264495 3.533103
H 4.208455 2.567779 1.848623
H 5.110429 3.344104 3.146095
C 3.111837 2.307777 -1.254197
H 2.635075 1.597387 -1.937155
C 4.445728 2.491656 -1.409147
H 4.916044 1.898612 -2.199583
C 5.374300 3.501631 -0.783303
H 6.076343 3.792426 -1.573835
H 5.996499 3.017708 -0.021902
C 4.734020 4.770057 -0.199316
H 4.141198 4.533083 0.685407
H 5.553055 5.401123 0.159514
C 3.927191 5.546331 -1.212668
H 4.462120 6.354387 -1.708698
C 2.658273 5.350599 -1.583833
H 2.265148 6.034369 -2.334742
C 1.643507 4.340048 -1.119835
H 1.060369 4.021831 -1.991546
H 0.919915 4.834276 -0.462340
C 2.147006 3.077784 -0.392613
H 2.571594 3.335079 0.577738
H 1.280321 2.440966 -0.197623
C -3.453732 1.078629 0.714566
C -2.871826 1.073303 1.981202
C -1.822706 1.913322 2.352993
C -1.307002 2.817034 1.439947
C -1.841561 2.853363 0.162162
C -2.867519 1.983864 -0.169897
C -5.827236 0.807808 -0.685294
C -6.136199 2.164948 -0.643287
C -7.224376 2.731231 -1.300571
C -8.080571 1.926928 -2.033408
C -7.833374 0.562853 -2.089873
C -6.736975 0.044346 -1.417812
C -5.491780 -0.689680 1.370760
C -6.077219 0.095988 2.363885
C -6.896255 -0.392582 3.370260
C -7.187994 -1.748381 3.407547
C -6.654253 -2.573794 2.432306
C -5.836165 -2.037443 1.442130
C -3.729960 -1.015542 -0.744211
C -2.905099 -1.940545 -0.105251
C -2.088875 -2.847981 -0.760399
C -2.021428 -2.826529 -2.143920
C -2.787535 -1.902396 -2.831899
C -3.616955 -1.028572 -2.132430
B -4.630311 0.042874 0.163094
C 7.043141 -0.027529 -2.454068
C 9.466737 -0.331076 -1.859009
H 7.246766 -0.688768 -3.301661
H 6.015681 -0.240736 -2.140337
H 7.104987 1.007670 -2.807896
H 9.568342 -1.171080 -2.552170
H 9.725777 0.578018 -2.412240
H 10.199535 -0.462901 -1.057176

158
$[(^{tBu}BDI)Mg^{+}(\textit{coe})][B(C_6F_5)_4^{-}]$ **(3)**
Mg -3.643455 -0.002455 -0.058826
F 5.371591 1.614600 2.447033
F 4.746364 -1.084007 3.026335
F 7.278475 1.210720 0.118628
F 2.847299 -2.198989 1.019104
F 6.587475 -2.841757 3.721951
F 6.490298 -1.043213 -1.386159
F 1.302809 0.156414 0.482249
F 6.206067 5.092053 -2.245767
F 8.018944 3.479784 -1.000033
F 8.413321 -3.753305 1.912094
F 3.575143 4.332324 -2.329143
F 2.804990 2.047479 -1.220363
F 8.329961 -2.828886 -0.657004
F 4.159034 0.154939 -2.889753
F 3.986938 2.908849 4.316446
F -0.036261 1.457471 2.337123
F 1.248189 2.849167 4.293523
F 1.800315 -4.108110 -0.507091
F 1.811007 -3.866780 -3.227387
N -4.366352 -1.796981 0.427976
F 3.009595 -1.695594 -4.369730
N -5.293688 1.038197 0.387494
C -5.547897 -1.976888 1.012558
C -6.355890 0.488731 0.981311
C -5.081856 2.415325 0.062104
C -3.472032 -2.763867 -0.129841
C -5.300487 2.862065 -1.254332
C 5.620950 -1.445015 2.068913
C -6.093226 -3.351697 1.506074
C 3.435585 0.769317 1.381791
C -3.686556 -3.199487 -1.453861
C -2.294826 -3.094122 0.564190
C 5.545506 -0.918845 0.782857
C -6.407746 -0.889781 1.280448
H -7.314544 -1.183336 1.778096
C -4.509120 3.264773 1.033373
C -2.660198 -3.893234 -2.095494
H -2.795519 -4.229285 -3.119004
C -1.294336 -3.786068 -0.118918
H -0.366861 -4.029843 0.386599
C 4.035270 1.525549 2.391832
C 6.571131 -2.381813 2.465320
C 6.487438 -1.446034 -0.101630
C -4.918038 4.164448 -1.586057
H -5.084964 4.529149 -2.595732
C -7.667694 1.249774 1.360864
C 4.997661 1.461383 -0.532962
C -5.014813 -2.928116 -2.148128
H -5.766720 -2.784306 -1.367863
C 7.451658 -2.379122 0.245735
C 6.315047 1.912507 -0.496115
C 7.497197 -2.852150 1.549761
C 2.048396 0.791691 1.416230
C -1.458752 -4.161499 -1.446905
H -0.659400 -4.680246 -1.965079
C 3.556517 -0.895495 -0.833691
C 6.735626 3.112366 -1.066072
C -5.981628 1.991641 -2.301134
H -6.188418 1.018059 -1.843890
C -4.141012 4.554035 0.653673
H -3.701319 5.224816 1.384204
C 5.819527 3.938494 -1.695809
C 3.341097 2.216689 3.374789
C -4.334481 5.004154 -0.648957
H -4.039093 6.010855 -0.926051
C -7.494108 -3.611914 0.911612
C -7.460854 -3.633580 -0.182623
H -8.233465 -2.866414 1.211087
H -7.854304 -4.587574 1.252818
C 1.953901 2.189894 3.369151
C 4.488726 3.549681 -1.735866
C 1.312265 1.473622 2.376305
C -7.668676 2.780602 1.200526
H -7.470906 3.113217 0.182918
H -6.955617 3.272602 1.863683
H -8.665640 3.140782 1.473441
C 4.121212 2.348208 -1.154459
C -5.234878 -4.582085 1.171750
H -5.722779 -5.459654 1.607582
H -4.232339 -4.524715 1.596296
H -5.143450 -4.759106 0.098777
C 2.922083 -2.025368 -0.313844
C -2.107407 -2.704259 2.022222
H -3.092582 -2.436877 2.419283
C -4.318345 2.786365 2.466558
H -5.094838 2.043528 2.673480
C -6.185710 -3.279168 3.047662
H -6.858972 -2.491022 3.392203
H -5.199497 -3.097435 3.487507
H -6.555738 -4.233550 3.436061
C -8.788910 0.697820 0.451383
H -9.728296 1.216960 0.668071
H -8.951702 -0.372563 0.595989
H -8.548490 0.860655 -0.604266
C 3.568010 -0.849921 -2.224665
C -8.015701 0.984107 2.843569
H -7.197320 1.297313 3.500101
H -8.237891 -0.062465 3.059213
H -8.902232 1.566303 3.113432
C 2.352747 -3.027857 -1.082224
C -1.528172 1.039665 -0.694316
C -0.259914 -0.342877 -3.467730
H -0.412907 -0.336019 -4.553124
H 0.230349 -1.292983 -3.228952
C -1.521068 -0.157143 -1.329587
C -1.564816 -3.859894 2.872389
H -2.169340 -4.764022 2.752322
H -1.572860 -3.580907 3.930439
H -0.532200 -4.107202 2.608281
C -5.083596 1.761446 -3.522985
H -4.857308 2.702104 -4.035226
H -5.571568 1.096074 -4.241915
H -4.131157 1.303107 -3.242436
C -1.656712 -0.328626 -2.811104
H -2.243780 0.486914 -3.243271
H -2.162459 -1.269459 -3.037106
C 2.986852 -1.821496 -3.037043
C 2.374729 -2.922145 -2.464793
C -0.396530 2.920076 -1.937282
H -0.557642 3.981529 -2.155975
H 0.388540 2.876660 -1.174163
C -4.474596 3.902014 3.505740
H -3.636652 4.605964 3.481091
H -4.502060 3.470061 4.510360
H -5.398505 4.468049 3.352265
C -7.325894 2.588565 -2.744184
H -7.995361 2.756668 -1.896599
H -7.828492 1.913042 -3.443960
H -7.183139 3.548852 -3.250555
B 4.386608 0.106485 0.199465
C -1.717753 2.381332 -1.349450
H -2.095213 3.089264 -0.605928
H -2.469076 2.338112 -2.144784
C -4.982631 -1.635501 -2.969310
C -5.490204 -4.101123 -3.011807
C 0.109967 2.233988 -3.216048
C 0.668930 0.804407 -3.059689
H -0.681456 2.253202 -3.979952
H 0.911897 2.869732 -3.603205
H 1.008352 0.655603 -2.030109
H 1.570249 0.713745 -3.672574
H -1.193269 1.057852 0.345427
H -1.211031 -1.039987 -0.765670
C -2.964107 2.093316 2.640879
H -2.800848 1.787218 3.678112
H -2.892213 1.177883 2.039776
H -2.141108 2.749390 2.345974
C -1.201831 -1.475718 2.155289
H -1.078649 -1.182870 3.201824
H -0.209987 -1.660557 1.731485
H -1.614781 -0.604021 1.634494
H -5.973718 -1.402025 -3.371291
H -4.677729 -0.775866 -2.360494
H -4.281874 -1.710066 -3.806880
H -6.510394 -3.917226 -3.363037
H -4.861772 -4.241167 -3.897093
H -5.487647 -5.037150 -2.445472

22
coe
C 0.762344 -1.557356 -0.446627
C -1.866586 0.489085 0.052997
H -2.343251 1.071135 0.852372
H -2.649925 0.279749 -0.686660
C -0.563914 -1.649999 -0.313285
C -1.370949 -0.837777 0.659149
H -0.768780 -0.617618 1.548009
H -2.237101 -1.412158 1.005998
C 1.712274 0.801534 -0.047873
H 2.560235 1.268871 0.468405
H 1.939063 0.854222 -1.121083
C 1.662844 -0.676066 0.385802
H 2.680817 -1.078133 0.341291
H 1.365320 -0.719540 1.441565
C 0.462081 1.650202 0.237985
C -0.783773 1.343703 -0.619388
H 0.210426 1.578386 1.305646
H 0.747314 2.695756 0.073881
H -0.466011 0.860071 -1.550378
H -1.255981 2.287371 -0.916098
H 1.245550 -2.144928 -1.225832
H -1.113592 -2.303140 -0.989671

20
cod

C	1.189509	1.241770	-0.503635
H	1.779041	1.844538	-1.194417
C	1.917540	0.018807	-0.008605
H	2.710143	0.341491	0.681566

H	2.442178	-0.419116	-0.868235
C	-0.039220	1.691928	-0.230243
H	-0.341583	2.605722	-0.740665
C	1.092387	-1.088711	0.668837
H	1.788299	-1.882241	0.962124
H	0.658276	-0.720902	1.600115
C	-1.189645	-1.241714	-0.503532
H	-1.779384	-1.844588	-1.194042
C	-1.092174	1.088541	0.669130
H	-1.787844	1.882032	0.963098
H	-0.657684	0.720175	1.599995
C	0.039089	-1.691948	-0.230279
H	0.341137	-2.605972	-0.740491
C	-1.917541	-0.018582	-0.008647
H	-2.710435	-0.341244	0.681199
H	-2.441805	0.419562	-0.868404

138

[(^{Me}BDI)Mg$^+$(*cod*)][B(C$_6$F$_5$)$_4$$^-$] (**4**)

Mg	3.367976	-0.063202	-0.755642
N	2.955768	1.578318	0.332847
N	2.910621	-1.430068	0.663588
C	3.691092	2.799333	0.253292
C	4.715696	3.027685	1.200484
C	3.437790	-2.759203	0.625237
C	1.522434	0.232788	1.753571
H	0.712449	0.318798	2.465080
C	3.458404	3.714391	-0.791762
C	1.992620	1.454505	1.262333
C	1.995781	-1.081044	1.566954
C	5.460868	4.203978	1.104877
H	6.241807	4.403904	1.832514
C	4.995792	2.042124	2.330268
H	4.504939	1.097322	2.075328
C	5.216717	5.131302	0.099364
H	5.799086	6.045682	0.047013
C	2.769463	-3.775565	-0.086610
C	4.226556	4.880162	-0.841477
H	4.048421	5.605692	-1.628790
C	2.392532	3.460838	-1.845635
H	2.207411	2.380762	-1.862415
C	4.683332	-3.002380	1.249377
C	3.397168	-5.018498	-0.211931
H	2.895442	-5.813727	-0.756177
C	5.358288	-1.928284	2.099495
H	5.157710	-0.958835	1.625727
C	1.316773	2.677983	1.843680
H	1.872902	3.597782	1.662602
H	1.148846	2.559696	2.915605
H	0.327596	2.771872	1.383016
C	5.264980	-4.261865	1.097505
H	6.221750	-4.473950	1.562562
C	1.370161	-3.587539	-0.656624
H	1.058963	-2.561222	-0.458186
C	4.636637	-5.261803	0.361428
H	5.106431	-6.234432	0.254380
C	1.357966	-2.117530	2.461517
H	0.371775	-2.362549	2.052518
H	1.203511	-1.717691	3.465250
H	1.940747	-3.036673	2.524149
C	2.835855	3.885189	-3.251085
H	2.855033	4.974546	-3.354923
H	2.133972	3.503934	-3.997327
H	3.837422	3.514937	-3.491818
C	6.489950	1.752898	2.527622
H	6.979724	1.417354	1.607907
H	6.614242	0.968033	3.280596
H	7.031694	2.633118	2.888013
C	1.062685	4.139795	-1.487565
H	0.640637	3.743412	-0.562997
H	0.327752	3.991489	-2.284795
H	1.207289	5.217855	-1.360093
C	4.390337	2.529111	3.655459
H	4.857004	3.469807	3.968353
H	4.553726	1.787789	4.444367
H	3.316202	2.700300	3.571157
C	6.878819	-2.094689	2.211322
H	7.143265	-2.946209	2.846814
H	7.315403	-1.203065	2.669630
H	7.360852	-2.242499	1.239256
C	0.360541	-4.519479	0.034400
H	0.545451	-5.565433	-0.233828
H	-0.657512	-4.266692	-0.275148
H	0.415651	-4.443790	1.122780
C	1.306955	-3.806724	-2.173722
H	1.999265	-3.154948	-2.712745
H	0.295775	-3.603734	-2.539270
H	1.555637	-4.839683	-2.439773
C	4.749742	-1.851845	3.508306
H	3.692449	-1.584779	3.484217
H	5.269935	-1.092060	4.101605
H	4.848622	-2.813474	4.022831
F	-0.595377	-0.947236	0.041006
F	-3.628297	1.926706	-2.187470
F	-1.670471	1.001207	2.029834
F	-2.690918	3.029629	0.204762
F	-6.251845	0.679594	-1.220857
F	-2.640161	-2.400586	-1.229804
F	-1.730014	2.537500	-3.955910
F	1.296677	-0.274788	-1.681421
F	-5.969567	-0.163416	1.456116
F	-4.346873	-2.771546	0.974727
F	0.753787	1.405423	-3.748891
F	-7.459486	-0.757033	-3.073873
F	-0.902530	-0.192088	4.280246
F	-7.302143	1.743031	2.763464
F	-4.035457	4.896155	1.496672
F	-6.312420	-3.024702	-4.062794
F	-3.873045	-3.815686	-3.111392
F	-3.521503	-3.954486	3.182385
F	-1.789142	-2.705132	4.882698
F	-6.360136	4.306092	2.799171
C	-3.025413	-0.822405	1.347866
C	-2.423497	1.378646	-2.001757
C	-4.291590	1.328191	0.707511
C	-2.213938	0.515289	-0.924058
C	-4.394073	-0.808144	-1.069547
C	-0.935556	-0.023808	-0.868761
C	-2.166595	-0.229776	2.273027
C	-3.472845	-2.085414	1.725248
C	-1.459357	1.708275	-2.945581
C	-5.621651	-0.438050	-1.613320
C	0.058365	0.305240	-1.777442
C	-3.847018	2.643817	0.784904
C	-0.195454	1.151787	-2.836496
C	-3.850226	-1.959589	-1.639268
C	-5.465871	1.082797	1.419929
C	-1.747865	-0.825421	3.450938
C	-6.278397	-1.167191	-2.602078
C	-6.174946	2.050569	2.114661
C	-5.699443	3.354628	2.136120
C	-4.462616	-2.715994	-2.624690
C	-3.068034	-2.731279	2.892433
C	-5.699170	-2.317207	-3.111752
C	-4.522707	3.649568	1.468463
C	-2.194807	-2.101034	3.762668
B	-3.497422	0.053941	0.022744
C	3.727744	-0.969973	-3.193959
H	2.685138	-1.281027	-3.231525
C	4.707877	-2.097672	-2.985687
H	4.840807	-2.622608	-3.940279
H	4.241397	-2.829547	-2.313939
C	3.961129	0.340891	-3.408995
H	3.086877	0.974118	-3.558667
C	6.088084	-1.721403	-2.412044
H	6.594384	-2.652227	-2.141964
H	6.708986	-1.237629	-3.166913
C	5.915114	0.472139	-1.098293
H	5.894949	0.906740	-0.099239
C	5.303182	1.013261	-3.555228
H	5.174841	1.894570	-4.189294
H	5.990234	0.356070	-4.089311
C	5.983698	-0.869349	-1.175234
H	5.981562	-1.418644	-0.240265
C	5.922120	1.478719	-2.220067
H	6.956646	1.795963	-2.401150
H	5.398808	2.374351	-1.869058

15

cht

C	-0.346745	1.526942	-0.275478
H	-0.636198	2.470433	-0.733791
C	0.962974	1.220381	-0.201219
H	1.693481	1.875262	-0.670276
C	1.450780	0.001619	0.535485
H	2.539632	0.002819	0.617569
H	1.035101	0.001155	1.553314
C	0.965739	-1.218278	-0.201089
H	1.697732	-1.871546	-0.670094
C	-0.343301	-1.527694	-0.275509
H	-0.630619	-2.471795	-0.733884
C	-1.425573	-0.681471	0.188515
H	-2.358679	-1.181132	0.440495
C	-1.427070	0.678326	0.188612
H	-2.361277	1.175854	0.440763

133

[(^{Me}BDI)Mg$^+$(*cht*)][B(C$_6$F$_5$)$_4$$^-$] (**5**)

Mg	-2.915727	-0.025254	-0.576784
F	-0.026176	-0.136331	0.023048
F	-1.538103	-0.168513	-2.105101
F	-0.468887	-0.032003	-4.604347
F	2.249978	0.244173	-4.888303
F	3.808916	0.347379	-2.743850
F	1.878740	-2.580835	-0.197575
F	2.927511	-4.878504	-1.037573
F	5.570752	-5.006209	-1.749909
F	7.098771	-2.753206	-1.611091
F	6.070067	-0.458173	-0.815505
F	2.971237	2.717857	-1.383018
F	4.545354	4.774707	-0.876988
F	6.531892	4.597096	0.987443
F	6.888064	2.255441	2.345809
F	5.318262	0.156330	1.847269
F	3.256407	-2.260597	2.173556
F	2.199076	-2.146134	4.576549
F	0.846152	0.075106	5.412241
F	0.564308	2.181925	3.712162
F	1.439919	2.020972	1.201636
N	-2.875664	-1.205681	1.032424
N	-2.835875	1.722843	0.415043
C	-4.724682	0.634248	-2.410464
H	-4.694519	1.654178	-2.024209
C	-5.246678	-0.340356	-1.617505
H	-5.579009	-0.083331	-0.612215
C	-5.541750	-1.720790	-2.133373
H	-6.067731	-2.310739	-1.383094
H	-6.175581	-1.638984	-3.027571
C	-4.233843	-2.374122	-2.489813
H	-3.934182	-3.260160	-1.935761
C	-3.467607	-1.919720	-3.503409
H	-2.593361	-2.498622	-3.792719
C	-3.700878	-0.702847	-4.253331
H	-3.267649	-0.659153	-5.248839
C	-4.251850	0.439985	-3.765817
H	-4.227133	1.326009	-4.393824
C	-1.852967	-1.743005	3.207743
H	-2.503816	-2.618050	3.254198
H	-1.800953	-1.276022	4.192186
H	-0.846960	-2.096736	2.950801
C	-2.288366	-0.758960	2.147619
C	-2.006391	0.593114	2.396420
H	-1.534131	0.791720	3.349345
C	-2.324953	1.744616	1.648282
C	-2.017997	3.067958	2.307447
H	-1.003114	3.365890	2.026687
H	-2.044833	2.981268	3.393647
H	-2.697805	3.858152	1.986735
C	-3.445114	-2.514622	0.964610
C	-4.818743	-2.648769	1.273823
C	-5.427442	-3.887481	1.072681
H	-6.480800	-4.014685	1.301618
C	-4.703775	-4.971677	0.585354
H	-5.193835	-5.927815	0.430881
C	-3.349050	-4.831119	0.318452
H	-2.785628	-5.687364	-0.041151
C	-2.693827	-3.610243	0.506532
C	-1.204430	-3.507847	0.218190
H	-0.863139	-2.516345	0.527267
C	-0.917844	-3.642834	-1.283974
H	-1.291053	-4.596160	-1.675014
H	0.156575	-3.595127	-1.472926
H	-1.400362	-2.838697	-1.848774
C	-0.408340	-4.541793	1.026799
H	-0.640765	-4.471178	2.093825
H	0.663121	-4.379083	0.894798
H	-0.632477	-5.563643	0.703062
C	-5.598130	-1.484735	1.872528
H	-5.122181	-0.561155	1.526063
C	-5.485999	-1.484017	3.405563
H	-5.901592	-2.408816	3.820444
H	-6.039349	-0.638662	3.827570
H	-4.448637	-1.400765	3.735381
C	-7.074292	-1.441737	1.464555
H	-7.211739	-1.475081	0.378999
H	-7.530625	-0.518317	1.834088
H	-7.641564	-2.271866	1.897447
C	-3.299950	2.940382	-0.179277
C	-4.662045	3.282048	-0.022088
C	-5.134746	4.441253	-0.637173
H	-6.175812	4.725473	-0.521198
C	-4.292916	5.241359	-1.402317
H	-4.675650	6.141668	-1.872411
C	-2.962695	4.881815	-1.562619
H	-2.310324	5.510318	-2.161953
C	-2.440220	3.733159	-0.960282
C	-0.973048	3.390401	-1.164270
H	-0.756952	2.470782	-0.611733
C	-0.046204	4.482800	-0.611775
H	-0.162663	5.417059	-1.171777
H	0.996571	4.167799	-0.695272
H	-0.256880	4.695976	0.438956
C	-0.667504	3.143254	-2.648408
H	-1.333054	2.386469	-3.078656
H	0.368815	2.814595	-2.772093
H	-0.795619	4.059655	-3.233868
C	-5.598236	2.413809	0.807172
H	-5.164130	1.409101	0.829514
C	-5.670459	2.882141	2.267633
H	-4.692587	2.842375	2.752901
H	-6.353646	2.243500	2.837914
H	-6.037968	3.912520	2.324287
C	-7.006706	2.308190	0.210399
H	-7.563303	3.244940	0.314858
H	-7.578297	1.536295	0.734730
H	-6.982371	2.050318	-0.853098
C	2.018113	0.120007	-1.218900
C	0.641534	-0.020076	-1.144146
C	-0.168439	-0.071593	-2.263938
C	0.337039	0.004456	-3.542719
C	1.709497	0.153252	-3.674103
C	2.497611	0.210048	-2.530595
C	3.916688	-1.398984	-0.411532
C	3.189412	-2.579910	-0.530996
C	3.703826	-3.788807	-0.965188
C	5.041818	-3.857262	-1.326683
C	5.814159	-2.709782	-1.248092
C	5.245614	-1.515751	-0.808986
C	4.086040	1.298984	0.180295
C	3.942328	2.521204	-0.465250
C	4.746456	3.628487	-0.216354
C	5.751486	3.545640	0.732720
C	5.928708	2.353017	1.421306
C	5.100630	1.277945	1.137897
C	2.393792	-0.129695	1.520845
C	2.553496	-1.152584	2.454104
C	2.031829	-1.112727	3.745184
C	1.350851	0.011397	4.176915
C	1.195315	1.073651	3.300339
C	1.684337	0.967893	2.009229
B	3.110642	-0.021391	0.027727

16

dmbd

C	1.026575	-1.529916	-0.000722
H	0.280027	-2.316402	-0.001295
H	2.064378	-1.849209	-0.000819
C	0.707437	-0.227269	0.000006
C	-0.707476	0.227285	0.000020
C	-1.026579	1.529929	-0.000734
H	-0.280011	2.316397	-0.001352
H	-2.064362	1.849315	-0.000753
C	1.793800	0.820099	0.000671
H	1.722146	1.464376	0.883530
H	1.723517	1.464164	-0.882475
H	2.781126	0.353209	0.001391
C	-1.793772	-0.820128	0.000659
H	-1.723758	-1.463787	-0.882803
H	-2.781137	-0.353280	0.001982
H	-1.721832	-1.464791	0.883202

134

[(^{Me}BDI)Mg$^+$(*dmbd*)][B(C$_6$F$_5$)$_4$$^-$] (**6**)

Mg	2.791777	-0.035512	-0.515839
F	0.109935	-0.156027	-0.041313
F	1.554134	-0.177996	-2.185920
F	0.433546	0.004305	-4.672169
F	-2.291223	0.297480	-4.887324
F	-3.797333	0.389677	-2.706951
F	-1.888725	-2.602008	-0.362077
F	-3.042150	-4.840260	-1.223396
F	-5.723766	-4.877507	-1.787894
F	-7.181276	-2.594911	-1.480233
F	-6.051514	-0.358728	-0.662013
F	-5.153817	0.173677	1.968019
F	-6.650092	2.299232	2.569317
F	-6.308290	4.651181	1.225149
F	-4.409413	4.811827	-0.730510

F	-2.905609	2.729656	-1.335560
F	-3.094182	-2.368695	2.090228
F	-1.986545	-2.337813	4.472349
F	-0.665936	-0.125204	5.379533
F	-0.454057	2.055605	3.764444
F	-1.348410	1.972661	1.257687
N	2.815098	1.736693	0.407213
N	2.936415	-1.173025	1.107088
C	2.062828	3.132136	2.291849
H	2.614994	3.946667	1.821676
H	2.268928	3.118789	3.363150
H	0.993832	3.329937	2.169119
C	2.366583	1.795014	1.660646
C	2.108591	0.658814	2.458798
H	1.684196	0.881386	3.428766
C	2.414717	-0.695743	2.242607
C	2.096064	-1.656617	3.363756
H	1.186684	-2.211074	3.102547
H	1.918367	-1.133901	4.303776
H	2.888343	-2.395243	3.504548
C	3.290692	2.900819	-0.276011
C	4.663841	3.224522	-0.171489
C	5.152215	4.298906	-0.915803
H	6.201544	4.566549	-0.847266
C	4.317077	5.039931	-1.744427
H	4.714790	5.873927	-2.313907
C	2.972281	4.712285	-1.832927
H	2.322024	5.302578	-2.471763
C	2.433719	3.644653	-1.108736
C	0.945799	3.352222	-1.217789
H	0.738046	2.428362	-0.668712
C	0.507783	3.157786	-2.675595
H	0.563339	4.096737	-3.235516
H	-0.530737	2.816492	-2.714321
H	1.140875	2.431829	-3.196659
C	0.108425	4.464548	-0.568564
H	0.395129	4.628793	0.472399
H	-0.952098	4.202897	-0.596366
H	0.240876	5.410432	-1.105118
C	5.591218	2.454808	0.761175
H	5.170808	1.451319	0.885241
C	7.017503	2.308770	0.216922
H	7.037515	1.925157	-0.808342
H	7.585725	1.619817	0.848274
H	7.553403	3.263335	0.224420
C	5.635625	3.088897	2.159994
H	5.996727	4.121539	2.102004
H	6.313221	2.525349	2.809919
H	4.652139	3.099896	2.632790
C	3.496338	-2.486453	1.051179
C	4.881011	-2.632033	1.293722
C	5.448397	-3.899747	1.158941
H	6.508613	-4.037409	1.344088
C	4.679109	-4.995889	0.784065
H	5.138294	-5.974315	0.683997
C	3.324349	-4.832317	0.533171
H	2.731619	-5.689652	0.229115
C	2.710130	-3.583486	0.658745
C	1.240100	-3.427304	0.309074
H	0.903632	-2.451422	0.669559
C	1.068556	-3.443606	-1.218759
H	1.681744	-2.673044	-1.703471
H	0.025014	-3.277483	-1.495291
H	1.385678	-4.406652	-1.633984
C	0.359764	-4.487993	0.980878
H	0.561812	-5.491008	0.591070
H	-0.694593	-4.271662	0.800695
H	0.528093	-4.508115	2.062139
C	5.735249	-1.438482	1.700236
H	5.264472	-0.548128	1.270383
C	7.170559	-1.513162	1.167991
H	7.750771	-2.294866	1.668931
H	7.685135	-0.566121	1.353443
H	7.202005	-1.711370	0.091429
C	5.748765	-1.236644	3.222682
H	4.748792	-1.038507	3.614251
H	6.383088	-0.383663	3.486349
H	6.144344	-2.125638	3.726005
C	4.923876	-0.250944	-1.611180
H	5.243944	-1.115800	-1.037006
H	5.218875	0.728487	-1.243395
C	4.342675	-0.371620	-2.829675
C	4.046574	-1.713262	-3.396878
C	3.382584	-1.830877	-4.554974
H	3.025228	-0.976704	-5.118047
H	3.172053	-2.809073	-4.975420
C	4.040069	0.870470	-3.631318
H	3.006864	0.890861	-3.985687
H	4.685858	0.900320	-4.514559
H	4.219993	1.774108	-3.041966
C	4.526720	-2.929051	-2.643319
H	5.616809	-2.923018	-2.537523
H	4.240637	-3.841400	-3.169255
H	4.106477	-2.991332	-1.633173
C	-1.975643	0.122667	-1.225601
C	-0.600217	-0.027482	-1.190198
C	0.183070	-0.077112	-2.327393
C	-0.349115	0.019761	-3.592652
C	-1.723447	0.182226	-3.688527
C	-2.483813	0.233418	-2.525966
C	-3.903813	-1.365658	-0.409498
C	-3.216085	-2.557904	-0.616934
C	-3.784474	-3.736870	-1.066266
C	-5.142000	-3.759293	-1.352698
C	-5.878073	-2.597195	-1.188139
C	-5.255340	-1.435257	-0.736944
C	-3.975871	1.313565	0.260336
C	-4.944251	1.300771	1.264851
C	-5.733889	2.389300	1.601521
C	-5.564165	3.587141	0.920008
C	-4.604475	3.661384	-0.075571
C	-3.837797	2.540627	-0.376397
C	-2.279890	-0.198054	1.510196
C	-2.401753	-1.262333	2.401735
C	-1.854289	-1.267375	3.682834
C	-1.188135	-0.148740	4.150246
C	-1.066222	0.951035	3.315813
C	-1.571857	0.887223	2.028590
B	-3.040561	-0.027009	0.044066

18

eb

C	-0.000072	1.421574	0.000231
H	-0.921650	1.994113	0.000172
H	0.921470	1.994168	0.000281
C	-0.000013	0.085925	0.000127
C	1.266419	-0.743274	0.000264
H	1.233475	-1.411153	-0.872013
H	1.233793	-1.410236	0.873268
C	2.586307	0.021046	-0.000354
H	2.678734	0.657271	-0.886223
H	3.428813	-0.676474	-0.000521
H	2.679390	0.657644	0.885173
C	-1.266389	-0.743328	0.000075
H	-1.233710	-1.410541	0.872885
H	-1.233440	-1.410941	-0.872404
C	-2.586269	0.021019	-0.000265
H	-2.678712	0.657496	-0.885950
H	-2.679264	0.657353	0.885462
H	-3.428793	-0.676481	-0.000598

136

[(^{Me}BDI)Mg$^+$(*eb*)][B(C_6F_5)$_4^-$] (**7**)

Mg	-2.669075	0.030278	-0.507217
F	-0.135680	0.156743	-0.143006
F	-1.535269	-0.078749	-2.291751
F	-0.372158	-0.510906	-4.731747
F	2.360573	-0.800034	-4.867806
F	3.828534	-0.634319	-2.667419
F	1.812875	2.557736	-0.642933
F	2.921794	4.734757	-1.697792
F	5.606979	4.784356	-2.244194
F	7.113910	2.574947	-1.717840
F	6.029464	0.397834	-0.704745
F	2.976362	-2.825568	-1.081299
F	4.528058	-4.793415	-0.253782
F	6.401641	-4.380546	1.689409
F	6.667446	-1.894261	2.785941
F	5.121743	0.116178	1.957964
F	1.367720	-1.865967	1.374062
F	0.482759	-1.773465	3.881726
F	0.642713	0.535426	5.318343
F	1.888042	2.706432	4.225194
F	2.985558	2.577303	1.839694
N	-2.731157	-1.688674	0.535809
N	-2.864396	1.277049	1.041675
C	-4.717795	0.269050	-1.731987
H	-5.040040	1.057641	-1.054900
H	-4.937754	-0.757347	-1.445904
C	-2.119204	-2.939755	2.570303
H	-2.719709	-3.762280	2.180672
H	-2.328854	-2.805623	3.632297
H	-1.064581	-3.215449	2.477912
C	-2.340725	-1.653956	1.809441
C	-2.078556	-0.466681	2.528966
H	-1.688250	-0.627403	3.524939
C	-2.365624	0.870535	2.216479
C	-2.067439	1.899104	3.283418
H	-1.190429	2.482440	2.978579
H	-1.850143	1.430366	4.243130
H	-2.888081	2.608754	3.408146
C	-3.180047	-2.917856	-0.048287
C	-4.563761	-3.203158	-0.017190
C	-5.017383	-4.370548	-0.631313
H	-6.075244	-4.611340	-0.613848
C	-4.136800	-5.236931	-1.268595
H	-4.507140	-6.142694	-1.738275
C	-2.783512	-4.936557	-1.301158
H	-2.099631	-5.616025	-1.801322
C	-2.279058	-3.779234	-0.701202
C	-0.786924	-3.499968	-0.784197
H	-0.594549	-2.526264	-0.323443
C	-0.313168	-3.447458	-2.244368
H	-0.337107	-4.441630	-2.702256
H	0.718499	-3.088005	-2.294483
H	-0.947256	-2.796469	-2.854664
C	0.034872	-4.542901	-0.012861
H	-0.272259	-4.607480	1.033253
H	1.095769	-4.283221	-0.044326
H	-0.084196	-5.536826	-0.457741
C	-5.545924	-2.278553	0.689920
H	-5.114112	-1.273200	0.674098
C	-5.717510	-2.650362	2.169876
H	-6.092776	-3.674668	2.269611
H	-6.434530	-1.974729	2.648765
H	-4.775038	-2.578006	2.717041
C	-6.913723	-2.213522	0.000147
H	-6.824213	-2.033129	-1.076199
H	-7.510599	-1.404395	0.429399
H	-7.481955	-3.139197	0.137085
C	-3.446998	2.578402	0.943833
C	-4.821838	2.719000	1.229355
C	-5.401328	3.982322	1.096253
H	-6.455281	4.116517	1.317942
C	-4.650984	5.076976	0.685165
H	-5.117572	6.052455	0.590773
C	-3.303010	4.917966	0.390327
H	-2.727027	5.776077	0.059774
C	-2.680332	3.674162	0.508841
C	-1.229605	3.495857	0.095064
H	-0.832639	2.613737	0.606079
C	-1.173527	3.218997	-1.415792
H	-1.800023	2.358120	-1.686496
H	-0.151932	3.018146	-1.745315
H	-1.562620	4.074146	-1.979491
C	-0.337080	4.681993	0.472546
H	-0.576426	5.577611	-0.110175
H	0.708904	4.439056	0.280103
H	-0.443755	4.928775	1.533253
C	-5.655342	1.532594	1.695273
H	-5.145221	0.626830	1.352518
C	-5.720781	1.451893	3.227765
H	-4.730917	1.313881	3.668241
H	-6.342181	0.605067	3.537458
H	-6.156909	2.366374	3.644800
C	-7.070444	1.524758	1.106724
H	-7.680097	2.346701	1.495353
H	-7.578964	0.595295	1.379824
H	-7.061320	1.600871	0.014380
C	1.978966	-0.238683	-1.251415
C	0.602744	-0.099278	-1.259103
C	-0.163128	-0.183132	-2.405996
C	0.391780	-0.410617	-3.644206
C	1.769640	-0.566605	-3.698285
C	2.510085	-0.482090	-2.524876
C	3.856456	1.371942	-0.559694
C	3.143708	2.523116	-0.881183
C	3.688525	3.669012	-1.433497
C	5.047556	3.697531	-1.711651
C	5.808626	2.573114	-1.436085
C	5.208970	1.443054	-0.883969
C	3.992178	-1.224937	0.367470
C	3.893369	-2.514295	-0.139960
C	4.686006	-3.576977	0.280484
C	5.633005	-3.374463	1.270334
C	5.764006	-2.108557	1.825774
C	4.949417	-1.082477	1.372706
C	2.238837	0.341894	1.446702
C	1.560988	-0.716184	2.053804
C	1.062947	-0.691831	3.344989
C	1.158604	0.474255	4.087670
C	1.787073	1.571476	3.526245
C	2.329040	1.479705	2.246104
B	3.021733	0.060165	0.010320
C	-4.295449	0.549899	-2.983803
C	-4.005282	-0.524131	-4.002448
H	-3.045148	-0.291406	-4.478119
H	-4.752953	-0.396694	-4.797438
C	-4.017292	-1.969248	-3.518570
H	-3.284987	-2.150603	-2.725932
H	-3.769204	-2.640361	-4.343993
H	-4.995813	-2.268729	-3.131879
C	-4.116229	1.959967	-3.489489
H	-4.570278	2.002433	-4.487677
H	-3.042148	2.113486	-3.666689
C	-4.672143	3.079660	-2.617129
H	-4.191371	3.123473	-1.636668
H	-5.748607	2.962533	-2.459311
H	-4.506552	4.045527	-3.10

3. References

[S1] Rigaku Oxford Diffraction **2018**, CrysAlisPro Software system, version 1.171.39.46, Rigaku Corporation, Oxford, UK.

[S2] Rigaku Oxford Diffraction **2018**, CrysAlisPro Software system, version 1.171.40.18b, Rigaku Corporation, Oxford, UK.

[S3] O. V. Dolomanov, L. J. Bourhis, R.J. Gildea, J. A. K. Howard and H. Puschmann, *J. Appl. Cryst.* **2009**, *42*, 339–341.

[S4] G. M. Sheldrick, *Acta Cryst. A* **2015**, *71*, 3–8.

[S5] G. M. Sheldrick, *Acta Cryst. C* **2015**, *71*, 3–8.

[S6] A. Thorn, B. Dittrich and G. M. Sheldrick, *Acta Cryst. A* **2012**, *68*, 448–451.

[S7] P. van der Sluis, A. L. Spek, *Acta Crystallogr. Sect. A* **1990**, *46*,194-201.

[S8] J.-S. Jiang, A. T. Brünger, *J. Molec. Biol.* **1994**, *243*, 100-115.

[S9] M. J. Frisch, G. W. Trucks, H. B. Schlegel, G. E. Scuseria, M. A. Robb, J. R. Cheeseman, G. Scalmani, V. Barone, G. A. Petersson, H. Nakatsuji, X. Li, M. Caricato, A. V. Marenich, J. Bloino, B. G. Janesko, R. Gomperts, B. Mennucci, H. P. Hratchian, J. V. Ortiz, A. F. Izmaylov, J. L. Sonnenberg, D. Williams-Young, F. Ding, F. Lipparini, F. Egidi, J. Goings, B. Peng, A. Petrone, T. Henderson, D. Ranasinghe, V. G. Zakrzewski, J. Gao, N. Rega, G. Zheng, W. Liang, M. Hada, M. Ehara, K. Toyota, R. Fukuda, J. Hasegawa, M. Ishida, T. Nakajima, Y. Honda, O. Kitao, H. Nakai, T. Vreven, K. Throssell, J. A. Montgomery, J. E. Peralta, F. Ogliaro, M. J. Bearpark, J. J. Heyd, E. N. Brothers, K. N. Kudin, V. N. Staroverov, T. A. Keith, R. Kobayashi, J. Normand, K. Raghavachari, A. P. Rendell, J. C. Burant, S. S. Iyengar, J. Tomasi, M. Cossi, J. M. Millam, M. Klene, C. Adamo, R. Cammi, J. W. Ochterski, R. L. Martin, K. Morokuma, O. Farkas, J. B. Foresman, D. J. Fox, *Gaussian 16 Rev. A.03*, Wallingford CT, **2016**.

[S10] J.-D. Chai, M. Head-Gordon, *Phys. Chem. Chem. Phys*. **2008**, *10*, 6615-6620.

[S11] W. J. Hehre, L. Radom, P. v. R. Schleyer, J. A. Pople, *Ab Initio Molecular Orbital Theory*, John Wiley, New York, **1986**.

[S12] T. Clark, J. Chandrasekhar, G. W. Spitznagel, P. v. R. Schleyer, *J. Comp. Chem*. **1983**, *4*, 294-301.

[S13] R. F. W. Bader, *Chem. Rev*. **1991**, *91*, 893-928.

[S14] T. A. Keith, *AIMAll (Version 17.01.25)*, TK Gristmill Software, Overland Park KS USA, **2017**.

[S15] Rigaku Oxford Diffraction, **2019**, CrysAlisPro Software system, version 1.171.40.67a, Rigaku Corporation, Oxford, UK.
